# Design of New Potent, Selective, and Long-Acting NOP Receptor Agonists through Multiple Sequential d‑Amino Acid Substitutions of [Arg^14^ Lys^15^]N/OFQ(1–15)-NH_2_

**DOI:** 10.1021/acs.jmedchem.6c01281

**Published:** 2026-07-23

**Authors:** Chiara Sturaro, Alessandra Rizzo, Erika Morrone, Erika Marzola, Pietro Pola, Alessia Frezza, Michela Argentieri, Federica Agosta, Antonella Ciancetta, Delia Preti, Salvatore Pacifico, Valentina Albanese, Giulio Meneguzzo, Davide Malfacini, Chiara Ruzza, Girolamo Calò, Remo Guerrini

**Affiliations:** † Department of Neuroscience and Rehabilitation, University of Ferrara, Via Luigi Borsari 46, Ferrara 44121, Italy; ‡ Department of Chemical, Pharmaceutical and Agricultural Sciences, University of Ferrara, Via Luigi Borsari 46, Ferrara 44121, Italy; § Department of Pharmaceutical and Pharmacological Sciences, Section of Pharmacology, University of Padova, Largo Meneghetti,2, Padova 35131, Italy; ∥ Technopole of Ferrara, Laboratory for Advanced Therapies (LTTA), Via Fossato di Mortara 70, Ferrara 44121, Italy

## Abstract

The nociceptin/orphanin FQ (N/OFQ) receptor (NOP) ligands are drug candidates for different diseases, but peptide ligands are often limited by rapid enzymatic degradation and short *in vivo* duration of action. Here, we applied a multiple D-amino acid substitution strategy to the peptide template [Arg^14^ Lys^15^]­N/OFQ­(1-15)-NH_2_ to improve metabolic stability while preserving receptor activity. Progressive substitutions revealed marked positional tolerance within the C-terminal address domain and identified [d-Lys^13,15^ d-Arg^14^]­N/OFQ­(1–15)-NH_2_ (compound **1c**) as the most promising compound. This led to the Cha^1^-containing analogue **3a**, which retained full agonist efficacy and high selectivity at the NOP receptor *in vitro* and *ex vivo*. *In vivo*, compound **3a** induced a long-lasting loss of the righting reflex in mice, closely overlapping the pharmacological profile of the potent and long-acting agonist UFP-112. These findings define the stereochemical tolerance of the NOP receptor toward multiple d-amino acid substitutions within the N/OFQ peptide and demonstrate that this information can be exploited as a rational design strategy to generate NOP receptor agonists with prolonged in vivo activity.

## Graphical abstract

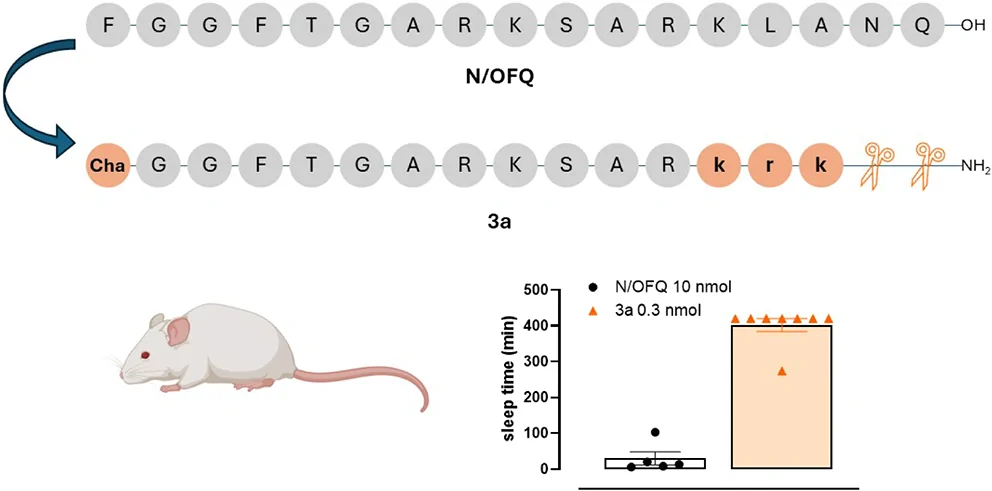

## Introduction

The endogenous neuropeptide nociceptin/orphanin FQ (N/OFQ) selectively activates the N/OFQ receptor (NOP), a member of the class A G protein–coupled receptor family.^1,2^ Although structurally related to classical opioid receptors, NOP displays a distinct pharmacological profile and ligand selectivity.^3^ N/OFQ is a 17–amino acid peptide (FGGFTGARKSARKLANQ) composed of two functionally distinct regions: an N-terminal “message” domain (Phe^1^–Phe^4^), which is required for receptor activation, and a C-terminal “address” domain (Arg^7^–Gln^1^ ^7^), which mainly contributes to binding affinity and receptor selectivity.^4^ NOP receptor activation leads to Gi/Go-mediated inhibition of adenylyl cyclase, increase in potassium conductance, and suppression of calcium channel activity.^3^ The N/OFQ–NOP system is broadly expressed throughout the central nervous system and is involved in the regulation of pain transmission, stress responses, emotional behavior, reward processing, learning and memory, neuroendocrine function, feeding behavior, and motor control.^3^ More recently, NOP receptor agonism has also been explored for the treatment of sleep disorders.^5,6^ Because of its broad physiological relevance, the NOP receptor has become an attractive target for medicinal chemistry programs focused on the identification of selective agonists and antagonists.

Since the discovery of the N/OFQ–NOP system, medicinal chemistry research has been directed toward the identification of peptide-based agonists displaying enhanced metabolic stability and improved bioavailability while retaining the high affinity and selectivity of the endogenous ligand. Early structure–activity relationship (SAR) studies demonstrated that N/OFQ(1–13)-NH_2_ is the shortest peptide fragment that preserves the binding affinity and full agonist activity of the native peptide.^7–9^ This truncated sequence was subsequently adopted as a template for extensive SAR investigations,^10–12^ which demonstrated that modifications at the N-terminal “message” domain critically modulate efficacy and potency. In particular, replacement of Phe^1^ with Nphe^1^ converted the peptide into a low-potency NOP antagonist,^13^ whereas substitution at position 4 with (pF)­Phe yielded highly potent agonists.^14,15^ In parallel, the introduction of the basic residues Arg and Lys at positions 14 and 15 of N/OFQ generated a highly potent NOP receptor agonist, [Arg^14^ Lys^15^]­N/OFQ.^16^ Combination of this C-terminal modification with N-terminal efficacy-reducing substitutions resulted in the development of [Nphe^1^ Arg^14^ Lys^15^N/OFQ-NH_2_ (UFP-101), a potent and selective NOP receptor antagonist that has since become a widely used pharmacological tool.^17,18^ Integration of multiple potency- and stability-enhancing modifications, including [(pF)­Phe^4^], [Aib^7^][Bibr ref19] , and the Arg^14^ Lys^15^ motif, yielded the long-acting NOP receptor agonist UFP-112 ([(pF)­Phe^4^ Aib^7^ Arg^14^ Lys^15^]­N/OFQ-NH_2_),^20^ now widely used as a reference ligand.^21^

In the present study, we selected [Arg^14^ Lys^15^N/OFQ­(1-15)-NH_2_ as the peptide template. To improve metabolic stability, multiple sequential d-amino acid substitutions, a strategy commonly employed to reduce enzymatic degradation of peptide ligands,^22,23^ were introduced in the address domain, either from the C-terminus toward position 7 (series I) or from the central peptide region toward the C-terminus (positions 7–14, series II) (Table 1). This sequential stereochemical substitution strategy was designed to define the degree and positional tolerance of the NOP receptor toward multiple d-amino acid substitutions within the N/OFQ peptide while preserving receptor activation. We reasoned that the N/OFQ address domain, unlike the N-terminal message domain, would better tolerate stereochemical modifications. The objective of this work was to define the stereochemical structure–activity relationships associated with sequential l-to-d substitutions in this region and establish design principles for the development of metabolically stable NOP peptide agonists.

**Table 1:**
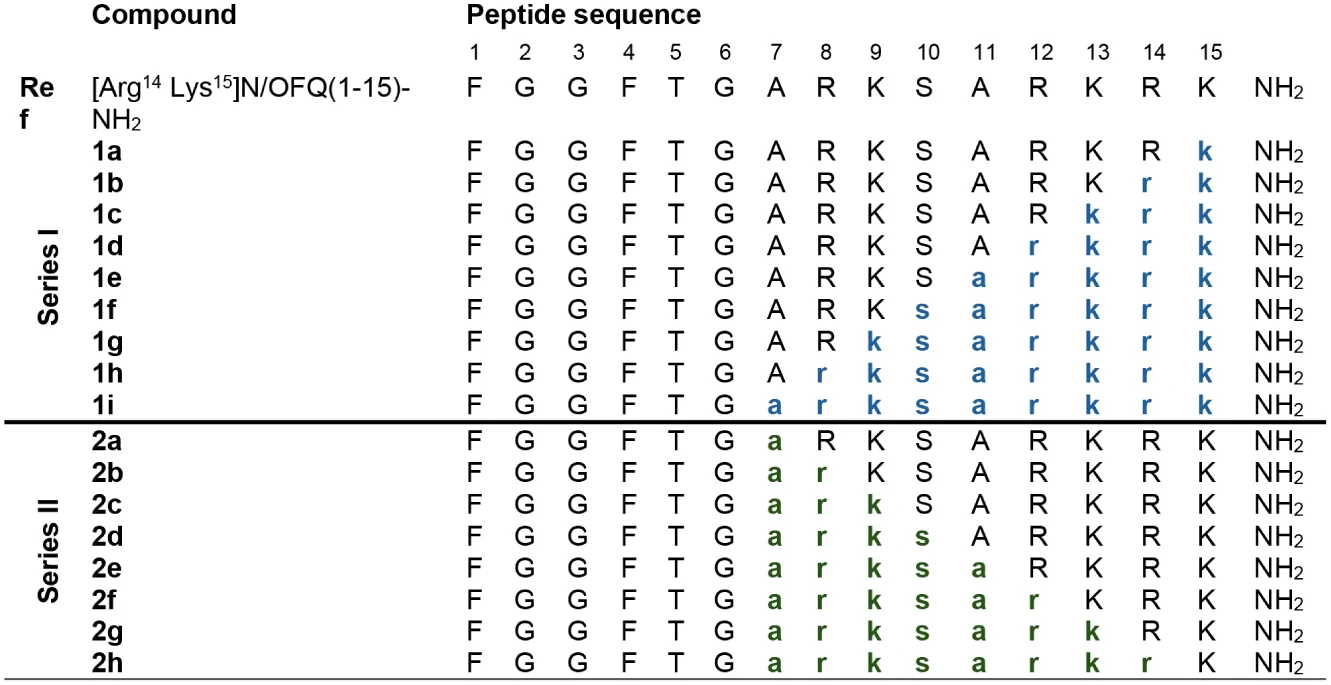
Sequential d-Amino Acid Substitution in the Address Domain of [Arg^14^ Lys^15^]­N/OFQ­(1-15)-NH_2_ (Series I and II)^a^

a d-Amino acids are indicated by lowercase letters.

The pharmacological activity of all compounds was initially assessed *in vitro* using recombinant cells expressing the NOP receptor and two complementary functional assays. Receptor activation was evaluated by measuring intracellular calcium mobilization in cells co-expressing the NOP receptor and chimeric G proteins,^24^ providing an amplified downstream functional readout, and by a bioluminescence resonance energy transfer (BRET) assay, which directly monitors NOP–G protein interaction.^25^ The analogue displaying the most favorable pharmacological profile was subsequently characterized *ex vivo* in the electrically stimulated mouse vas deferens (mVD).^8^ Finally, in view of the well-established hypnotic effects of NOP receptor agonists,^5,6,26–28^ this compound was also investigated *in vivo* for its ability to induce loss of the righting reflex (RR) in wild-type and NOP knockout mice.

## Results

### Synthesis of N/OFQ-Related Peptides

Three series of novel N/OFQ derivatives were synthesized, and their structures are schematically depicted in Table 1 (series I–II) and Figure 1A (series III). The first two series of analogues were obtained by the sequential replacement of natural amino acids in the address domain with the respective d-amino acids, starting from the C-terminus toward residue 7 (series I, compounds **1a–1i**) or from position 7 up to position 14 (series II, compounds **2a–2h**). The third series (compounds **3a–d**) originated from key modifications of the message fragment of compound **1c** from series I (see below). All peptides were synthesized via solid-phase peptide synthesis (SPPS) using standard Fmoc/tBu chemistry and purified by semi-preparative HPLC. The degree of purity, greater than 95%, for all compounds was confirmed by analytical HPLC. Compound **3a**, which was assayed in the *in vivo* experiment, was characterized by HRMS (Supplementary Information, S14).

**Figure 1:**
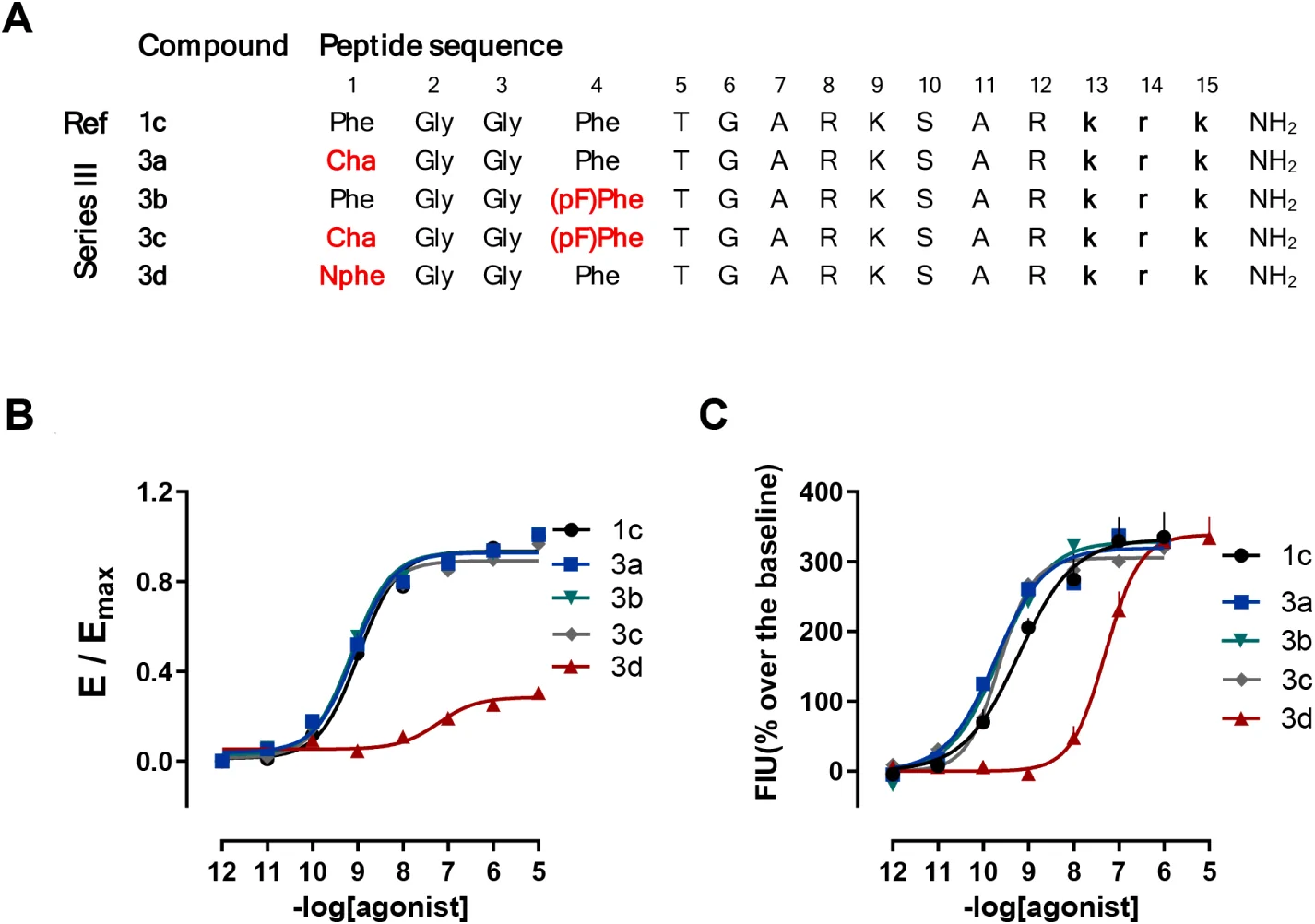
Chemical structure and pharmacological activity of **1c** analogues. Panel A: sequences of compounds **3a–d** in comparison with **1c**. Panel B: concentration–response curves to compounds **1c** and **3a–d** in the NOP–G protein interaction assay. Panel C: concentration–response curves to compounds **1c** and **3a–d** in the NOP calcium mobilization assays. Data are the mean ± SEM of 5 experiments conducted in duplicate.

### ***In Vitro*** **Pharmacological Activity**

The *in vitro* pharmacological activity of N/OFQ-NH_2_ and its analogues was evaluated using complementary BRET^25^ and calcium mobilization^24^ assays. N/OFQ-NH_2_ produced concentration-dependent NOP-G-protein coupling and intracellular calcium mobilization (Table 2 and Supplementary Figures 1 and 2). As recently confirmed,^28^ the pharmacological profile of N/OFQ-NH_2_ is comparable to that of the endogenous ligand. In the NOP–G protein interaction assay, N/OFQ­(1-15)-NH_2_ displayed potency and efficacy values comparable to those of N/OFQ-NH_2_, whereas the [Arg^14^ Lys^15^] derivatives were more potent than both N/OFQ-NH_2_ and N/OFQ­(1-15)-NH_2_. In the calcium mobilization assay, N/OFQ-NH_2_, N/OFQ­(1-15)-NH_2_, and the two [Arg^14^ Lys^15^] derivatives exhibited similar potency and efficacy values. [Arg^14^ Lys^15^]­N/OFQ­(1-15)-NH_2_ was selected as the reference scaffold to investigate the effects of multiple sequential substitutions with d-amino acids.

**Table 2:** *In Vitro* Pharmacological Activity of N/OFQ-NH_2_, N/OFQ­(1-15)-NH_2_, [Arg^14^ Lys^15^] Analogues, and the Reference Agonist UFP-112^a^

	NOP-G protein interaction		Ca^2+^ mobilization	
	pEC_50_ (CL_95**%**_)	*E*_max_± SEM	pEC_50_ (CL_95**%**_)	*E*_max_± SEM
N/OFQ-NH_2_	7.99 (7.40–8.58)	1.00	9.00 (8.68–9.31)	322 ± 17
N/OFQ(1-15)-NH_2_	7.64 (7.14–8.14)	0.99 ± 0.09	8.87 (8.48–9.26)	347 ± 21
[Arg^14^ Lys^15^]N/OFQ-NH_2_	8.37 (7.67–9.08)	0.98 ± 0.07	8.78 (7.88–9.68)	316 ± 12
[Arg^14^ Lys^15^]N/OFQ(1-15)-NH_2_	8.40 (7.86–8.95)	1.10 ± 0.13	8.64 (7.59–9.69)	313 ± 13
UFP-112	9.39 (8.93–9.86)	0.93 ± 0.09	9.05 (8.49–9.52)	300 ± 23

a pEC_50_ values are expressed as mean (CL_95%_); *E*_max_ values are expressed as mean ± S.E.M; *N* = 5 experiments performed in duplicate.

Two series of analogues were generated: the first incorporating multiple sequential d-amino acid substitutions from position 15 to position 7 (Table 3, Supplementary Figures 3 and 4), and the second from position 7 to position 14 (Table 4, Supplementary Figures 5 and 6). The pharmacological activity of these analogues was evaluated in both functional assays and compared with that of [Arg^14^ Lys^15^]­N/OFQ­(1-15)-NH_2_.

**Table 3:** *In Vitro* Pharmacological Activity of [Arg^14^ Lys^15^]­N/OFQ­(1-15)-NH_2_ Analogues with Multiple Sequential d-Amino Acid Substitutions from Position 15 to 7^a^

	NOP-G protein interaction		Ca^2+^ mobilization	
	pEC_50_ (CL_95**%**_)	*E*_max_ ± SEM	pEC_50_ (CL_95%_)	*E*_max_ ± SEM
**[Arg** ^**14**^ **Lys** ^**15**^ **]N/OFQ(1-15)-NH**_**2**_	8.52 (7.84–9.19)	1.00	9.69 (9.02–10.36)	304 ± 15
**1a**	9.52 (8.53–10.51)	0.89 ± 0.05	9.68 (9.20–10.17)	275 ± 39
**1b**	9.51 (8.24–10.78)	0.87 ± 0.03	9.31 (8.37–10.25)	287 ± 26
**1c**	9.49 (8.68–10.29)	0.88 ± 0.04	9.65 (9.28–10.03)	255 ± 33
**1d**	8.21 (7.24–9.19)	0.87 ± 0.02	9.26 (8.75–9.77)	258 ± 29
**1e**	7.67 (6.87–8.47)	0.83 ± 0.02	8.65 (8.43–8.86)	233 ± 52
**1f**	7.38 (6.70–8.07)	0.85 ± 0.04	9.18 (7.81–10.54)	295 ± 29
**1g**	7.78 (7.24–8.33)	0.82 ± 0.04	8.96 (7.36–10.55)	304 ±27
**1h**	6.99 (6.51–7.46)	0.84 ± 0.03	7.78 (6.36–9.20)	300 ± 44
**1i**	6.76 (6.15–7.37)	∼0.70	7.29 (6.77–7.81)	287 ± 24

a pEC_50_ values are expressed as mean (CL_95%_); *E*_max_ values are expressed as mean ± S.E.M; *N* = 5 experiments performed in duplicate.

**Table 4:** *In Vitro* Pharmacological Activity of [Arg^14^ Lys^15^]­N/OFQ­(1-15)-NH_2_ Analogues with Progressive d-Amino Acid Substitutions from Position 7 to 14^a^

	NOP-G protein interaction		Ca^2+^ mobilization	
	pEC_50_ (CL_95**%**_)	*E*_max_± SEM	pEC_50_ (CL_95**%**_)	*E*_max_± SEM
**[Arg** ^**14**^ **Lys** ^**15**^ **]N/OFQ(1-15)-NH**_**2**_	8.70 (8.46–8.94)	1.00	8.74 (8.24–9.25)	331 ± 21
**2a**	7.88 (7.60–8.16)	0.84 ± 0.07	8.57 (7.61–9.52)	328 ± 28
**2b**	7.42 (7.04–7.81)	0.66 ± 0.08*	7.51 (6.76–8.26)	328 ± 30
**2c**	7.30 (7.08–7.52)	0.63 ± 0.07*	7.50 (7.08–7.91)	304 ± 47
**2d**	7.54 (6.47–8.62)	0.70 ± 0.11	7.02 (6.55–7.49)	266 ± 42
**2e**	7.39 (6.41–8.38)	0.65 ± 0.12*	6.68 (6.12–7.24)	276 ± 40
**2f**	7.26 (6.52–7.99)	0.80 ± 0.13	7.36 (6.70–8.02)	283 ± 35
**2g**	7.15 (6.58–7.73)	0.76 ± 0.10	7.42 (6.83–8.01)	299 ± 30
**2h**	7.14 (6.65–7.64)	0.72 ± 0.07	7.19 (6.57–7.81)	256 ± 49

a pEC_50_ values are expressed as mean (CL_95%_); *E*_max_ values are expressed as mean ± S.E.M; *N* = 5 experiments performed in duplicate. *E_max_ is significantly different from [Arg^14^ Lys^15^]­N/OFQ­(1–15)-NH_2_ (non-overlapping CL_95%_).

All synthesized [Arg^14^ Lys^15^]­N/OFQ­(1-15)-NH_2_ analogues containing progressive d-amino acid substitutions retained *in vitro* activity at the NOP receptor. In the first series, where d-amino acid substitutions were progressively introduced from the C-terminus toward the central region of the peptide (positions 15 to 7, Table 3 and Supplementary Figures 3 and 4), no changes in potency and efficacy were observed up to position 13. Of note, compounds **1a**, **1b**, and **1c** displayed potencies similar to or slightly higher than that of the parent [Arg^14^ Lys^15^]­N/OFQ­(1-15)-NH_2_, maintaining full agonist efficacy in both assays. A modest reduction in potency (∼10-fold) became evident only when d-amino acid substitutions extended beyond position 12, with a more pronounced decrease observed for analogues **1h** and **1i** (∼100-fold reduction in potency in the calcium mobilization assay and ∼30-fold reduction in potency in the BRET assay).

In the second series, characterized by progressive d-amino acid substitutions from position 7 toward the C-terminus (positions 7–14, Table 4 and Supplementary Figures 5 and 6), a reduction in potency was already detectable upon introduction of a single d-amino acid at position 7. In the NOP–G protein interaction BRET assay, compound **2a** displayed an approximately 10-fold decrease in potency relative to [Arg^14^ Lys^15^]­N/OFQ­(1-15)-NH_2_, an effect that was not observed in the calcium mobilization assay, where comparable potency values were maintained. Further extension of d-amino acid substitutions resulted in a progressive loss of potency in both assays, becoming evident from compound **2b** and increasing with additional substitutions, reaching an approximately 30-fold reduction for compound **2h**. Among these analogues, compound **2e** exhibited the lowest potency, showing an approximately 100-fold decrease relative to the reference peptide in the calcium mobilization assay.

With respect to efficacy, all compounds displayed full agonist activity in the calcium mobilization assay. In contrast, in the NOP–G protein interaction BRET assay, compounds **2b**, **2c**, and **2e** displayed reduced maximal responses, with intrinsic activity values (α) of approximately 0.6, indicative of partial agonist behavior.

Based on its optimal balance of potency, efficacy, and structural modification, compound **1c** was used as the scaffold for subsequent chemical modifications (Figure 1A). Specifically, substitutions at key positions of **1c** were introduced to modulate efficacy and improve *in vivo* stability, including replacement of Phe^1^ with Cha^1^ (**3a**),^29^ introduction of (pF)­Phe^4^ to enhance agonist potency (**3b**),^14,15^ their combination (**3c**), and substitution of Phe^1^ with Nphe^1^ to reduce efficacy (**3d**).^13,17,18^

In the NOP–G protein interaction BRET assay, compounds **3a**, **3b**, and **3c** displayed potency and maximal effects comparable to those of the parent compound **1c** (Figure 1B). The calcium mobilization assay yielded similar results, with all three analogues displaying similar potency and full agonist efficacy relative to compound **1c** (Figure 1C). In contrast, in the NOP–G protein interaction BRET assay, compound **3d** exhibited partial agonist behavior, with reduced potency (pEC_50_ = 7.22) and intrinsic activity (α ≈ 0.3) (Figure 1B). In the calcium mobilization assay, compound **3d** behaved as a full agonist with markedly reduced potency (pEC_50_ = 7.29), corresponding to an approximately 100-fold decrease compared with compound **1c** (Figure 1C). In parallel experiments, in the NOP–G protein interaction BRET assay, UFP-101 displayed low-efficacy partial agonism (α = 0.25), whereas it was inactive in the calcium mobilization assay up to 10 μM (Supplementary Figure 7). Given its low efficacy in the BRET assay, compound **3d** was subsequently evaluated for antagonist activity by assessing its ability to shift the N/OFQ concentration–response curve to the right, in comparison with the reference NOP antagonist UFP-101. As expected, UFP-101 acted as a competitive NOP antagonist, displaying a pA_2_ value of 8.97 (8.13–9.80). Under the same experimental conditions, compound **3d** also acted as a NOP antagonist, with a pA_2_ value of 8.51 (8.41–8.61) (Supplementary Figure 7).

Based on its preserved high potency and full agonist profile in both *in vitro* functional assays, together with the incorporation of the non-natural Cha residue expected to improve resistance to enzymatic degradation, compound **3a** was chosen for subsequent *in vitro* and *in vivo* pharmacological characterization. In contrast, introduction of (pF)­Phe^4^ did not confer any additional advantage over Cha^1^, whereas substitution of Phe^1^ with Nphe^1^ yielded a low-potency partial agonist in the NOP–G protein interaction assay, rather than a pure antagonist; thus, these analogues were not further pursued.

The receptor selectivity of compound **3a** was investigated in CHO cells co-expressing the mu, delta, and kappa opioid receptors together with the chimeric G proteins. As expected, Dermorphin, DPDPE ([d-Pen^2^ ,d-Pen^5^]­enkephalin), and Dynorphin A elicited robust concentration-dependent calcium responses in cells expressing the mu, delta, and kappa receptors, respectively. Compound **3a** did not evoke detectable responses at concentrations up to 1 μM (Figure 2), demonstrating at least 1000-fold selectivity for the NOP over classical opioid receptors.

**Figure 2:**
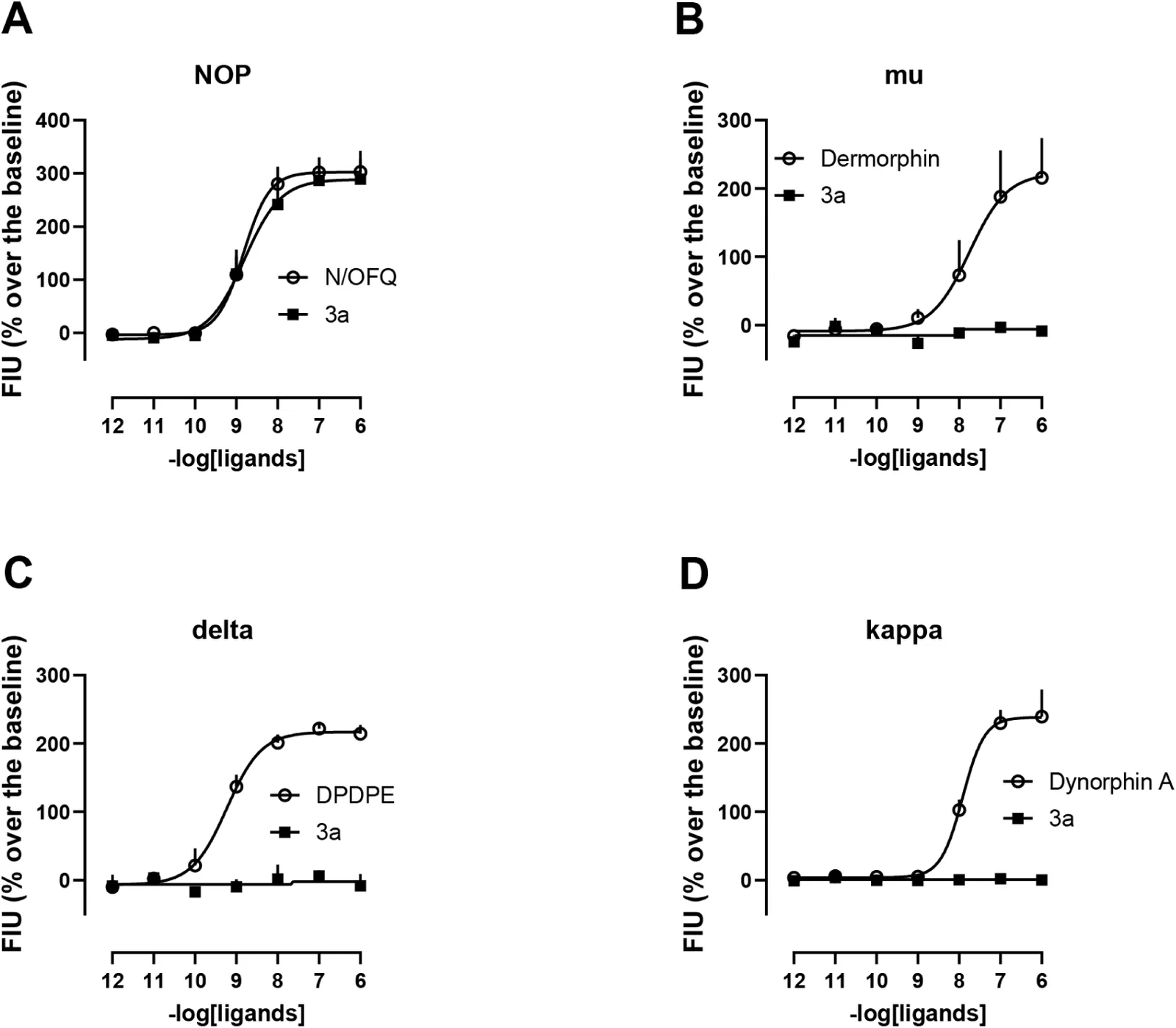
Compound **3a** selectivity of action at the NOP receptor by calcium mobilization. Concentration–response curves to N/OFQ, dermorphin, DPDPE, dynorphin A (i.e., standards), and compound **3a** in CHO cells stably expressing the NOP (panel A), mu (panel B), delta (panel C), and kappa (panel D) receptors and chimeric G proteins. Data are the mean ± S.E.M. of 5 experiments conducted in duplicate.

In the electrically stimulated mouse vas deferens (mVD), N/OFQ produced a concentration-dependent inhibition of the electrically evoked twitch response, displaying a pEC_50_ of 7.61 (7.44–7.78) and a maximal inhibition of 91 ± 2%. The reference long-acting NOP receptor agonist UFP-112 elicited a similar maximal effect (92 ± 3%) but with higher potency (pEC_50_ = 8.73 (8.36–9.10)) (Figure 3A). The N/OFQ analogues [Arg^14^ Lys^15^]­N/OFQ­(1–15)-NH_2_ and compound **1c** mimicked the inhibitory action of N/OFQ, with the same potency and efficacy as the natural peptide, while compound **3a** displayed the same efficacy but 3-fold lower potency (pEC_50_ = 7.19 (6.93–7.45)) (Figure 3B).

**Figure 3:**
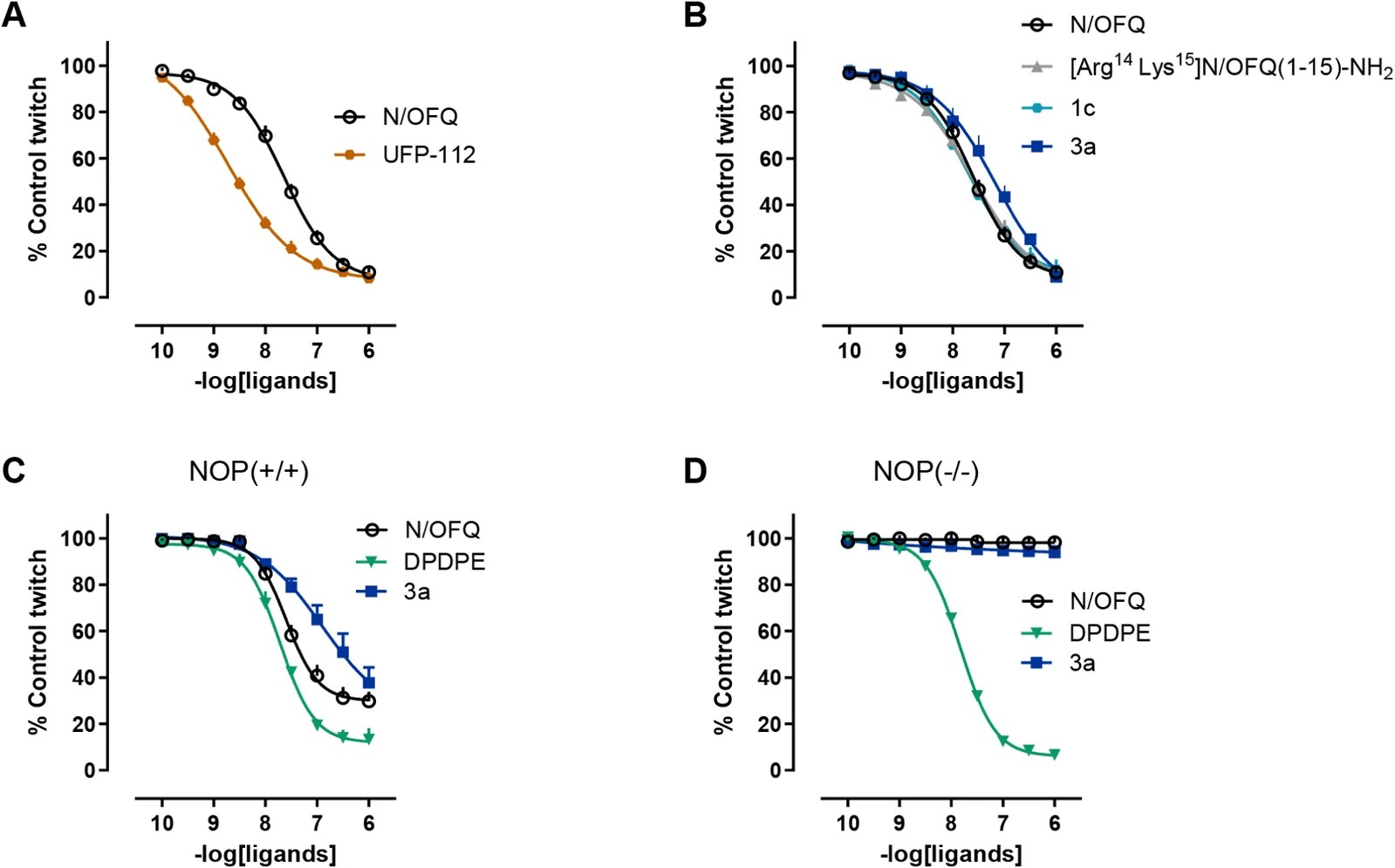
Mouse vas deferens bioassay. Concentration–response curves to N/OFQ, UFP-112 (panel A), [Arg^14^ Lys^15^]­N/OFQ­(1-15)-NH_2_, compounds **1c** and **3a** (panel B). Concentration–response curves to DPDPE, N/OFQ, and compound **3a** in tissue taken from NOP­(+/+) (panel C) and NOP­(-/-) mice (panel D). Data are the mean ± S.E.M. of 5 (panels A and B) or 4 (panels C and D) experiments.

To verify the receptor mechanism underlying the effects of compound **3a** in the mVD, experiments were repeated using tissues from NOP wild-type (NOP­(+/+)) and NOP knockout mice (NOP­(−/−)). The concentration-response curve to the selective delta agonist DPDPE was unaffected by NOP receptor deletion, being superimposable in tissues from both genotypes. Conversely, neither N/OFQ nor compound **3a** produced any inhibitory effect in preparations obtained from NOP (−/−) mice (Figure 3D), confirming that their actions are mediated by NOP receptor activation.

The kinetic profile of compound **3a** was then compared to that of N/OFQ and UFP-112. N/OFQ induced a rapid inhibition of the electrically evoked twitch, which was completely and rapidly reversible upon washout. In contrast, UFP-112 displayed a slower onset of action, and its inhibitory effect persisted after washing. Compound **3a** showed an intermediate behavior, with an onset slower than that of N/OFQ but faster than that of UFP-112, and reduced reversibility upon washout compared with that of N/OFQ, although this effect was less pronounced than that of UFP-112 (Supplementary Figure 9).

### Molecular Dynamics

We carried out a molecular modeling analysis aimed at rationalizing the trend observed *in vitro*. Peptides **1a**-**1i** and the [Arg^14^ Lys^15^]­N/OFQ­(1–15)-NH_2_ reference were subjected to a 100 ns Molecular Dynamics (MD) trajectory of the peptide-receptor system embedded in a solvated membrane environment (system details in Supplementary Table 1). As shown in Figure 4 and Video S1, the NOP receptor complex with [Arg^14^ Lys^15^]­N/OFQ­(1–15)-NH_2_ is stable in the simulated time frame thanks to a tight interaction pattern between the peptide and the residues in the receptor orthosteric site. Along with established interactions involving the FGGF message moiety, such as the salt bridge between the peptide N-terminus and the conserved D130^3.32^ residue, and stabilizing salt-bridge interactions between positively charged residues in the address moiety, namely Arg^8^, Lys^9^, Arg^12^, and Lys^13^, with negatively charged residues located in the TM2-ECL2 region, already described in previous N/OFQ^30^ and N/OFQ(1–13)-NH_2_ ^31^ MD studies, the introduction of two additional positive charges in the peptide C-terminus, namely Arg^14^ and Lys^15^, contributes to further stabilizing the address α-helix. In particular, Arg^14^ H-bond with S293^ECL3^ and Lys^15^ salt-bridge with E295^7.29^ provide an additional anchor to the NOP receptor TM7 extracellular region. As shown in the panels in Figure 4B and Supplementary Table 2, the complex Root Mean Square Deviation (RMSD) and the Root Mean Square Fluctuation (RMSF) analysis reveal remarkable stability of both the receptor and the peptide during the simulated time, with average RMSD and RMSF below 1.5 and 1.2 Å, respectively.

**Figure 4:**
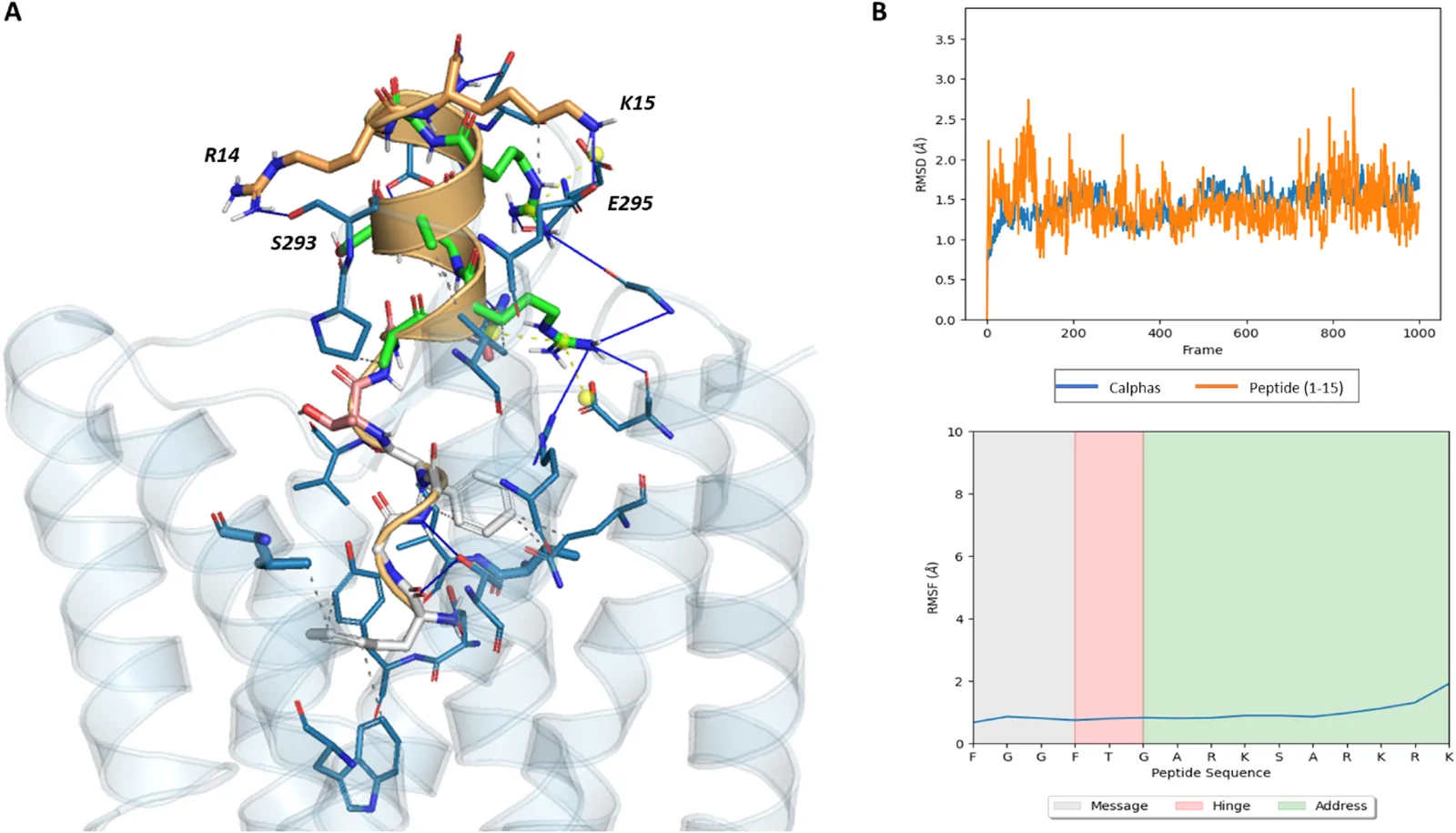
Analysis of the reference peptide [Arg^14^ Lys^15^]­N/OFQ­(1–15)-NH_2_. (A) receptor–peptide interaction pattern: the peptide is represented in orange cartoons, with message, hinge, and address (7–13) residues highlighted with gray, pink, and green sticks, respectively. Arg^14^ and Lys^15^ are shown as orange sticks. Receptor residues interacting with the peptide are shown as blue sticks. Interaction types are represented according to PLIP representation: H-bonds as blue continuous lines, hydrophobic interactions as gray dotted lines, and salt bridges as yellow dotted lines, with the charge center shown as a yellow sphere. Arg^14^ and Lys^15^ contribute to complex stability through salt-bridge and H-bond interactions with E295^7.29^ and S293^ECL3^, respectively. (B) Top: RMSD profile of the receptor (blue) and peptide (orange) during the simulated time. Bottom: peptide average RMSF profile. The initial complex was generated using the Cryo-EM structure with PDB ID: 8F7X as template.

The introduction of a d-amino acid at position 15 in **1a** preserves the complex stability. Although the salt bridge with E295^7.29^ is lost, the terminal carboxy-amide group interacts with S293^ECL3^ via an H-bond and contributes to stabilizing the C-terminal α-helix, as shown in the RMSF profile (Figure 5A and Video S2). In the **1b** complex, the Lys^15^ residue establishes a salt bridge with E295^7.29^ and an H-bond with S293^ECL3^ via its terminal carboxy-amide group. The configuration inversion at position 14 does not produce any additional interaction (Figure 5B and Video S3). The additional configuration inversion of the amino acid at position 13 in **1c** generates a new peptide conformation where d-Lys^13^ is projected toward negatively charged residues in the ECL2. In particular, d-Lys^13^ establishes a bidentate salt bridge with E194^ECL2^ and E196^ECL2^, partially shared with Lys^12^, while D-Lys^15^ is projected toward TM5 near D209^5.32^ (Figure 5C and Video S4).

**Figure 5:**
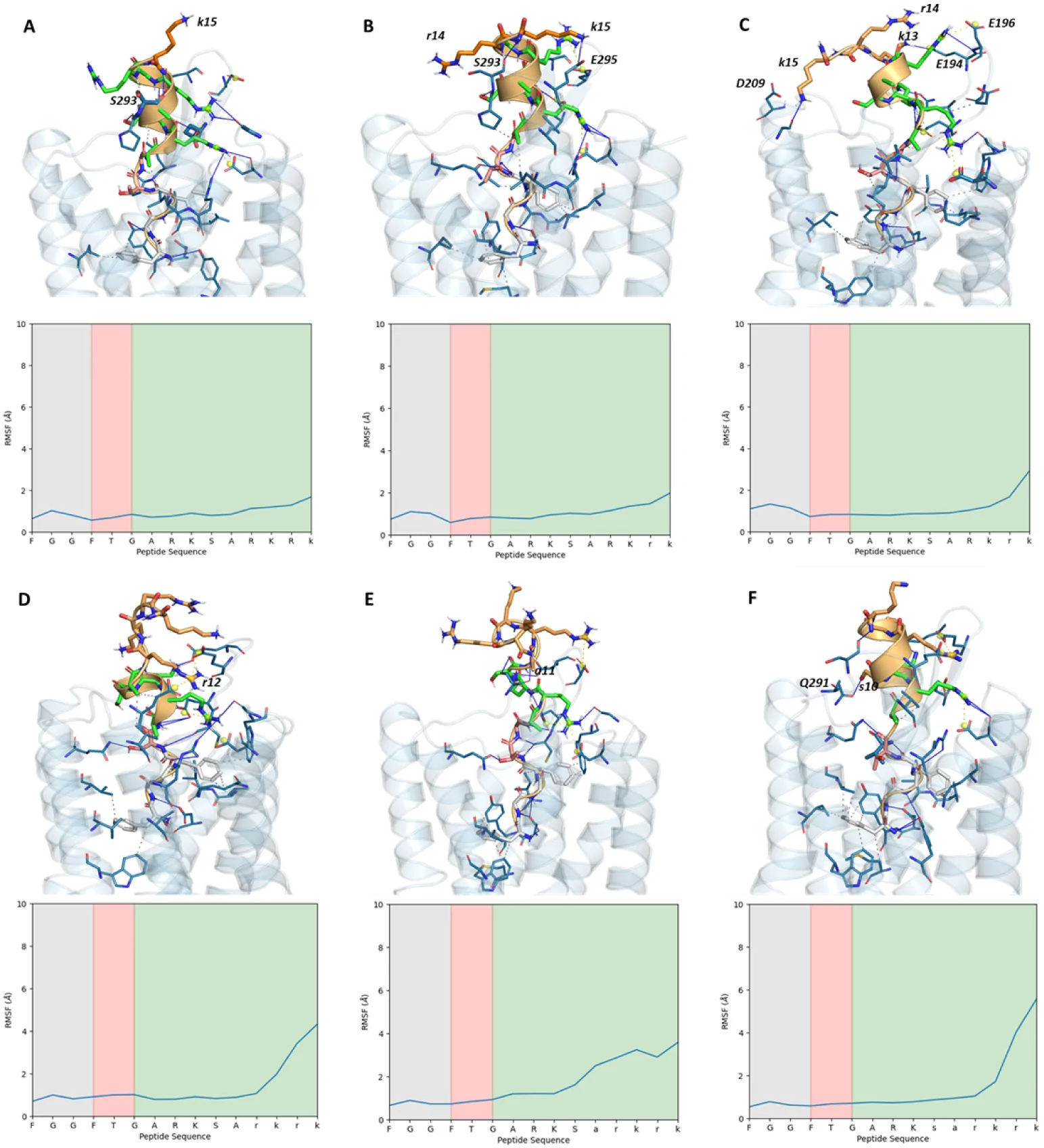
Analysis of peptides **1a**–**1f**. Top panels: peptide–receptor interaction pattern observed during the trajectory. Bottom panels: peptide average RMSF profile. All the complexes are stable, with average receptor RMSF below 1.5 Å. As far as the peptide is concerned, compounds **1a**–**1c** show average RMSF values below 1.2 Å, whereas **1d**–**1f**, due to the loss of anchoring interactions involving residues in the C-term (from residue 12 to 15), show average RMSF values above 1.6 Å. D-Arg^14^ residue in **1b–1c** (panels B–C, respectively) does not establish specific interactions, while the inversion of configuration in residues at position 15 (d-Lys^15^ in **1a–1c** shown in panels A–C, respectively) and 13 (d-Lys^13^ in **1c**, panel C) preserves the complex stability through a network of H-bonds and salt bridges. (D) d-Arg^12^ in **1d** establishes a salt-bridge network with negatively charged residues in receptor ECL2, but the interaction pattern of the peptide C-terminal residues is lost with a consequent stability reduction. (E) d-Ala^11^ in **1e** does not establish additional interactions, while d-Ser^10^ in **1f** (panel F) interacts with Q291^ECL3^ through an H-bond. The initial complexes were generated using the Cryo-EM structure with PDB ID: 8F7X as template.

A reduction in potency is observed when a progressive further configuration inversion is introduced in adress residues 12 to 10. The MD analysis helps rationalize this observation with a partial loss of the above-described interactions. In the **1d** complex, the d-Lys^12^ residue reaches E197^ECL2^ through a salt bridge, but the previously described interaction pattern involving d-Lys,^13^ d-Arg,^14^ and d-Lys^15^ is partially lost, resulting in decreased stability, as demonstrated by the average RMSF spike observed for the C-terminal residues shown in Figure 5D and Video S5. D-Ala^11^ in **1e** does not engage in specific interactions (Figure 5E and Video S6), while D-Ser^10^ in **1f** is anchored to Q291^ECL3^ (Figure 5F and Video S7) via an H-bond.

A further configuration inversion in residues 7–9 leads to progressively less potent peptides (*e.g.*, **1h** and **1i** with pEC_50_ for the NOP–G protein interaction assay below 7). This can be ascribed to a partial or total loss of peptide secondary structure (Supporting Information Figure S10). Trajectory visualization (Videos S8-S10) revealed that the inversion of configuration in position 8, in particular, causes the lack of anchoring interactions with nearby receptor residues, as the D-Arg^9^ side chain engages in an intramolecular H-bond with Ala^7^ and Phe^4^ backbone carbonyl in **1h** and **1i**, respectively.

### In Vivo Studies

Because N/OFQ and other NOP receptor agonists are known to induce loss of the righting reflex (RR) in mice,^26,28^ the *in vivo* activity of compound **3a** was assessed using this experimental paradigm. The effects of compound **3a** were directly compared with those of N/OFQ and the reference agonist UFP-112. At the high dose tested (10 nmol), N/OFQ induced RR loss in fewer than half of the treated animals, and the effect was short-lived (Figure 6). In contrast, UFP-112 caused RR loss in 88% of mice at both 0.3 nmol and 1 nmol. Animals responding to UFP-112 remained asleep until the experimental cutoff of 420 minutes, irrespective of the dose administered. Superimposable results were obtained with compound **3a**, which induced a comparable, long-lasting loss of the RR at both 0.3 and 1 nmol (Figure 6). Notably, while only a few minutes were sufficient to observe the onset of the effect of N/OFQ, several tens of minutes were required for the development of the RR loss induced by both UFP-112 and compound **3a**.

**Figure 6:**
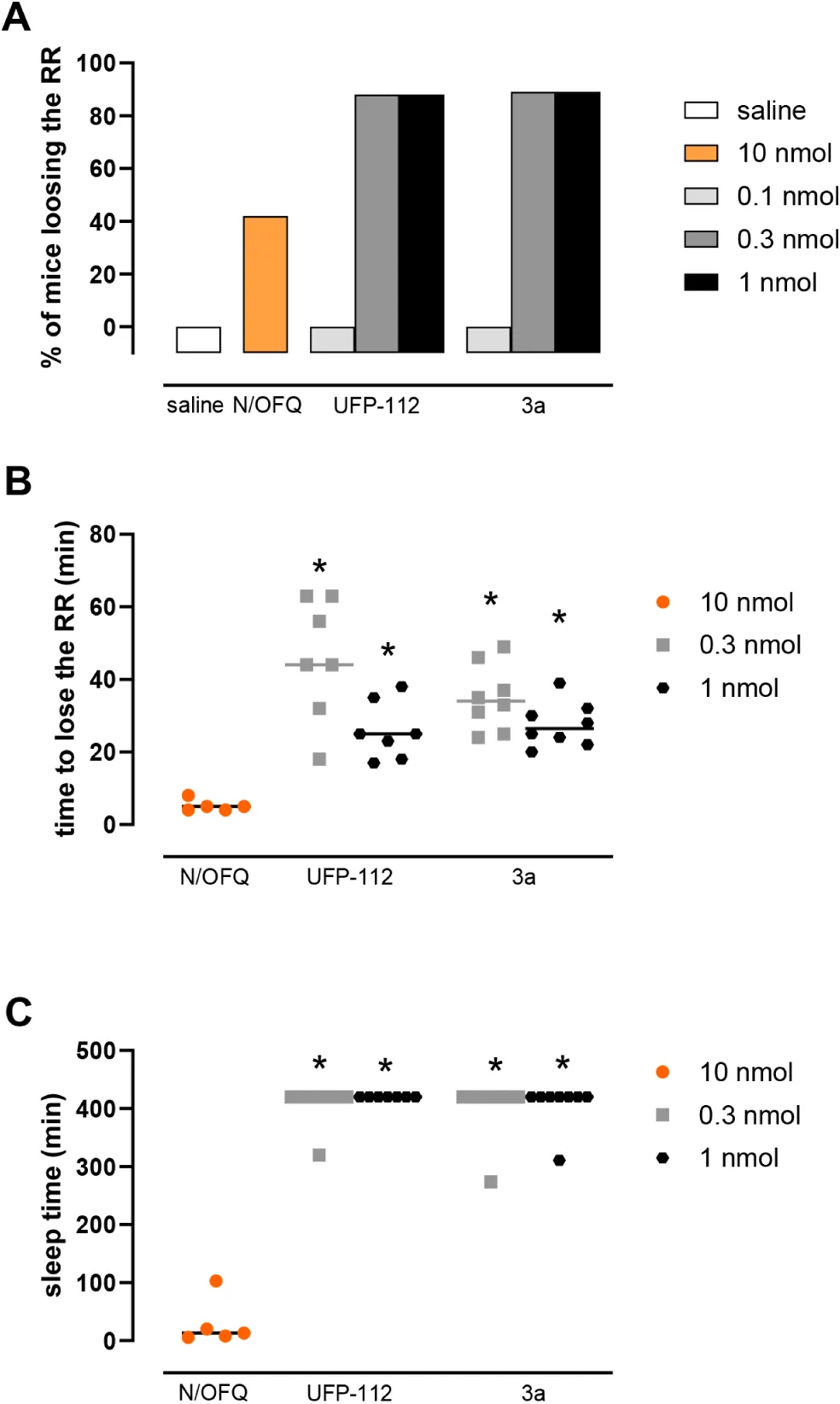
Loss of the RR assay. Panel A shows the percentage of animals that lost the RR; panel B shows the latency to lose the RR from compound i.c.v. injection; panel C shows sleep duration. Sleep duration is defined as the interval between loss and recovery of the RR. Data are expressed as a percentage (RR loss) or mean and S.E.M. (latency and sleep duration). N/OFQ, *N* = 12 mice per group; UFP-112, *N* = 8 mice per group; compound **3a**, *N* = 9 mice per group. Graphs B and C include only those animals that lost the RR. Latency: *p < 0.05 vs N/OFQ, according to one-way ANOVA followed by Dunnett’s post hoc test (F­(4,30) = 13.42). Sleep time: *p < 0.05 vs N/OFQ, according to Kruskal–Wallis test followed by Dunn’s post hoc test (Kruskal–Wallis statistic = 23.46, *P* = 0.0001).

The *in vivo* selectivity of compound **3a** was further assessed in NOP­(−/−). Following i.c.v. administration (1 nmol), compound **3a** induced a long-lasting loss of the RR in NOP­(+/+) mice but was completely inactive in NOP­(−/−) animals (Figure 7).

**Figure 7:**
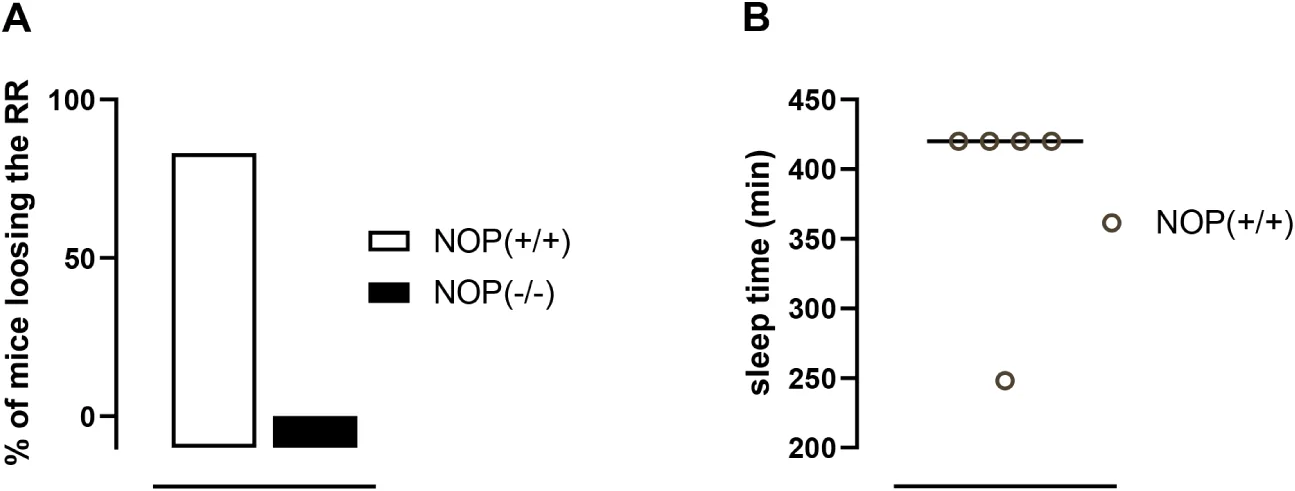
Loss of the RR assay. Compound **3a** (1 nmol, i.c.v.) in NOP­(+/+) and NOP­(−/−) mice. Panel A shows the percentage of animals that lost the RR; panel B shows their sleep duration. Sleep duration is defined as the interval between loss and recovery of the RR. Data are expressed as a percentage (RR loss) or mean ± S.E.M. (sleep duration). *N* = 6 mice per group; sleep-duration graphs include only those animals that lost the RR.

## Discussion

In this study, we investigated a sequential d-amino acid substitution strategy applied to the NOP receptor agonist [Arg^14^ Lys^15^]­N/OFQ­(1-15)-NH_2_ to improve metabolic stability while preserving receptor potency, efficacy, and selectivity. This approach led to the identification of [Cha^1^ D-Lys^13,15^ D-Arg^14^]­N/OFQ­(1–15)-NH_2_ (**3a**), which retains full agonist activity, high potency, NOP selectivity, and displays long-lasting NOP-mediated effects *in vivo*.

To overcome the intrinsic limitations of peptide-based therapeutics, including rapid enzymatic degradation and short duration of action *in vivo*, several medicinal chemistry strategies have been explored to improve metabolic stability while preserving receptor potency and efficacy. Among these, the introduction of non-natural amino acids, particularly those of the D-series, represents a well-established approach to reduce proteolytic susceptibility without substantially altering pharmacological activity.^22,23^ In the present study, this strategy was applied to a minimal, fully active N/OFQ-derived template extended with the potency-enhancing Arg^14^–Lys^15^ motif,^16^ *i.e.*, [Arg^14^ Lys^15^]­N/OFQ­(1-15)-NH_2_. Multiple progressive d-amino acid substitutions were introduced with the aim of maximizing metabolic stability without altering pharmacodynamic properties. To preserve the N-terminal message domain required for NOP receptor activation,^4,33,34^ d-amino acid substitutions were confined to the C-terminal address domain of the peptide. Although the use of d-amino acids to improve peptide stability is well established, little information is available regarding the extent to which multiple sequential stereochemical inversions can be accommodated by the NOP receptor without compromising agonist activity. Therefore, the primary objective of this work was to define the stereochemical tolerance of the NOP receptor toward progressive l-to-d substitutions within the address domain of the N/OFQ peptide.

For *in vitro* profiling, compounds were evaluated using complementary NOP–G protein interaction and calcium mobilization assays. These assays differ in both the proximity of the measured signaling event and the degree of signal amplification. The BRET assay directly measures receptor–G protein coupling and therefore provides a proximal assessment of receptor activation, whereas the calcium mobilization assay relies on chimeric G proteins to redirect signaling toward intracellular calcium release, generating a more amplified downstream functional response. The combined use of these assays therefore allows a more comprehensive pharmacological characterization of ligand efficacy, which may depend on the signaling system employed. Accordingly, ligand classification as full or partial agonists throughout this work always refers to the specific functional assay employed, as discussed by Kenakin (Chapter 2).^35^ Systematic analysis of the first two series of [Arg^14^ Lys^15^]­N/OFQ­(1-15)-NH_2_ analogues bearing cumulative d-amino acid substitutions (from position 15 to 7 and from position 7 to 14) revealed a positional dependence of stereochemical tolerance along the peptide sequence. When d-amino acids were introduced from the C-terminus toward the central region (positions 15 to 7), the NOP receptor tolerated multiple substitutions without substantial loss of potency and efficacy. In particular, replacement of up to three residues within the distal address domain (compounds **1a**–**1c**) preserved agonist potency while maintaining full efficacy in both assays. These findings indicate that the C-terminal portion of the peptide is permissive to stereochemical modification and can accommodate multiple d-amino acids without compromising ligand–receptor interaction, as shown in the MD analysis of **1a**–**1c**. The modeling analysis, in particular, suggested that potency retention is driven by positions 13 and 15, with d-Lys residues engaging in additional salt bridges with residues in ECL2 and TM5. A progressive loss of potency became evident only when substitutions extended beyond position 12, with more pronounced effects observed for analogues containing d-amino acids closer to the central region. This can be ascribed to the loss of the peptide secondary structure, which is held in place by an H-bond between the backbone carbonyl of the residue in position 7 and the backbone amide group of the residue in position 11. Our MD analysis, in particular, highlighted the role played by D-Arg^9^ in disrupting the α-helix by establishing an intramolecular H-bond through its side chain with the backbone carbonyl of residues in positions 4 and 7, therefore causing the loss of anchoring electrostatic interaction with the receptor. In contrast, the introduction of d-amino acids starting from the central region toward the C-terminus (positions 7–14) produced an immediate impact on ligand potency. Notably, substitution of a single residue at position 7 was sufficient to reduce potency in the NOP–G protein interaction assay, indicating that this region displays limited tolerance to stereochemical inversion. However, the impact of d-Ala^7^ substitution within the [Arg^14^ Lys^15^]­N/OFQ­(1-15)-NH_2_ template (compound **2a**) appears less pronounced than that previously reported for the native N/OFQ sequence. This effect is likely attributable to the additional anchoring electrostatic interactions established by the positively charged Arg^14^ and Lys^15^ side chains, which contribute to holding the peptide in the orthosteric binding site and compensate for the α-helix disruption caused by the inversion of configuration in position 7. SAR studies showed that [d-Ala^7^]­N/OFQ, despite exhibiting comparable binding affinity (*K*_i_) to the natural peptide, behaved as a markedly less potent partial agonist in cAMP inhibition assays, with approximately 30-fold reduced potency and intrinsic activity below 0.5. Further substitutions led to a progressive decrease in potency and, for selected analogues, a reduction in intrinsic efficacy in the BRET assay. This loss of efficacy was not evident in the calcium mobilization assay. This apparent discrepancy is consistent with the higher degree of signal amplification associated with the calcium assay, which may mask partial agonism by allowing ligands with reduced intrinsic efficacy to generate maximal downstream responses. Similar assay-dependent differences in efficacy have been widely described for GPCR ligands and emphasize the value of combining proximal and downstream functional assays during pharmacological characterization.^35,36^ On the basis of these SAR data, compound **1c** was identified as the optimal balance between chemical modification and pharmacological performance. This analogue incorporates the highest number of d-amino acid substitutions compatible with preserved potency and full agonist efficacy, thereby maximizing the potential for resistance to enzymatic degradation while retaining the pharmacological profile of the parent peptide.

Further optimization of the **1c** scaffold focused on the introduction of modifications previously shown to modulate NOP receptor pharmacology through distinct mechanisms. Specifically, substitution of Phe^1^ with the non-natural amino acid Cha^1^ (compounds **3a** and **3c**) was explored to improve metabolic stability;^29^ introduction of (pF)­Phe^4^ (compound **3b** and, in combination, compound **3c**) was investigated to enhance agonist potency;^14,15^ and replacement of Phe^1^ with Nphe^1^ (compound **3d**) was employed to reduce efficacy and generate an antagonist profile.^13,17,18^ Neither the introduction of Cha^1^ at position 1, nor substitution of Phe^4^ with (pF)­Phe, nor the combination of these two modifications produced significant changes in the *in vitro* pharmacodynamic profile of compound **1c**. Compounds **3a**, **3b**, and **3c** displayed potency and full agonist efficacy comparable to those of the parent scaffold in both the NOP–G protein interaction and calcium mobilization assays, indicating that these substitutions did not confer additional *in vitro* advantages. Nevertheless, in the case of compound **3a**, the presence of a non-natural amino acid at the N-terminal position may be expected to enhance resistance to enzymatic degradation, particularly by aminopeptidases—an effect that is not captured by the recombinant cell-based assays employed here. Given that N/OFQ is rapidly degraded in plasma by aminopeptidases^37–39^ incorporation of a non-natural N-terminal residue may provide a stability advantage. Moreover, regarding the known endopeptidase cleavage sites of N/OFQ (specifically Ala^11^Arg^12^ and Lys^13^ ^39^), the simultaneous D-aa substitution at positions 13 to 15 is intended to promote a protective effect that can extend to the neighboring positions, thereby contributing to peptide stabilization.^40^ For this reason, compound **3a** remained an interesting candidate for further evaluation in isolated tissue preparations, which better approximate physiological conditions, and subsequently *in vivo*.

In contrast, substitution of Phe^1^ with Nphe^1^, which in previous studies led to potent NOP receptor antagonists such as [Nphe^1^]­N/OFQ­(1–13)-NH_2_ ^13^ and UFP-101,^13,17,18^ resulted here in a different pharmacological outcome. In fact, compound **3d** exhibited a loss of potency and retained intrinsic activity, behaving as a low-potency NOP partial agonist. This is a rather unexpected finding considering that compound **3d** differs from [Nphe^1^]­N/OFQ­(1-13)-NH_2_ and UFP-101 only at the C terminus, sharing the same N-terminal message domain Nphe-Gly-Gly-Phe. Structural findings may help to interpret the present results. In the inactive NOP^41^ the benzyl ring of Nphe^1^ in UFP-101 interacts with the receptor residues Tyr131^3.33^ and Met134^3.36^ . In the active NOP^42^ the benzyl ring of Phe^1^ in N/OFQ occupies the same hydrophobic chamber but engages additional hydrophobic contacts with residues Ile219^5.42^, Val279^6.51^, and Val283^6.55^, in addition to π–π interactions with Tyr131^3.33^. Thus it is possible to speculate that the D-amino acid substitutions at positions 13, 14, and 15 of compound **3d** by reorganizing the C-terminal interaction network with ECL2, similarly to what was observed for compound **1c**, may slightly alter the positioning of the Nphe^1^ benzyl ring allowing the interaction not only with Tyr131^3.33^ but also with Ile219^5.42^, Val279^6.51^, and Val283^6.55^ and thus explaining the partial agonist behavior of this NOP ligand. This view is consistent with the structural evidence provided by Wang et al.^42^ and Thompson et al.,^41^ and with the computational data reported by Daga and Zaveri,^43^ and highlights the sensitivity of the Nphe^1^ pharmacophore to structural modifications in the peptide address domain. The partial agonist profile of compound **3d** limited its suitability for further development within this series.

The electrically stimulated mVD provides a well-established *ex vivo* model for evaluating the pharmacological activity of NOP receptor ligands in their native tissue environment.^44^ In this assay, the pharmacological activity of compound **3a** was directly compared with that of the potent and long-lasting NOP receptor agonist UFP-112.^20,21^ In the present study, UFP-112 behaved as a full NOP receptor agonist and displayed an approximately 30-fold higher potency than N/OFQ, in agreement with previous reports.^20^ Compound **3a** also behaved as a full NOP receptor agonist in the mVD, producing maximal inhibition of electrically evoked contractions comparable to those elicited by both N/OFQ and UFP-112. In terms of potency, compound **3a** was modestly less potent than N/OFQ and markedly less potent than UFP-112. Regarding the kinetics of action, N/OFQ induced a rapid and fully reversible inhibition of the twitch response; compound **3a** was characterized by a slower onset of action and an incomplete recovery upon washout. This kinetic behavior closely mirrors that described for UFP-112 in isolated tissue assays, where its effects are characterized by a delayed onset and slow, often incomplete, reversibility after washing.^20^

Although compound **3a** was less potent than both N/OFQ and UFP-112 in the mVD assay, its *in vivo* profile proved markedly different. *In vivo*, compound **3a** induced loss of the RR in mice with approximately 30-fold higher potency than N/OFQ. Moreover, whereas the effects of N/OFQ were short-lived, lasting only a few minutes, compound **3a** produced a remarkably prolonged loss of the RR that reached the experimental cutoff of 7 h. The *in vivo* pharmacological profile of compound **3a** therefore closely overlaps with that of UFP-112 with regard to potency and persistence of action. In the literature, UFP-112 has been reported to be 10–100-fold more potent than N/OFQ, depending on the species and assay employed, including rodents^20,45^ and non-human primates,^46^ and to elicit effects lasting several hours. The behavior of UFP-112 observed here is fully consistent with these previous findings.

With respect to duration of action, compound **3a** proved indistinguishable from UFP-112 in the RR assay, with both ligands reaching the experimental cutoff of 7 h. A similar outcome has been reported in studies exploring peptide multimerization as a strategy to obtain long-acting NOP receptor agonists, where multimeric N/OFQ derivatives, administered i.c.v., reached the assay cutoff for duration of action.^28^ A different profile has been reported for non-peptide NOP agonists, including Ro 64–6198 ^6^ and sunobinop,^5,47^ which produce sleep episodes lasting approximately 3–5 h in rats, as assessed by EEG recordings. The prolonged RR suppression observed with peptide agonists following i.c.v. administration suggests that additional factors may contribute to the persistence of the behavioral effect. One possibility is that central delivery allows high and sustained local concentrations of peptide in specific brain regions, a scenario that may differ substantially from systemic administration of small-molecule ligands. Under these conditions, the RR assay may not permit fine discrimination between compounds with extended duration of action once the experimental cutoff is reached. Nevertheless, the assay clearly distinguishes short-lived peptides such as N/OFQ and N/OFQ(1–13)-NH_2_ ^28^ from long-acting analogues, including multimeric derivatives,^28^ UFP-112, and compound **3a**. Thus, the present results support the classification of compound **3a** as a potent NOP receptor agonist with prolonged *in vivo* activity, although additional studies will be needed to fully characterize its pharmacokinetic profile. In addition, compound **3a** displayed high NOP selectivity, and its effects were present in NOP­(+/+) but absent in NOP­(−/−) mice both *ex vivo* and *in vivo*, demonstrating that its pharmacological activity is strictly NOP mediated.

## Conclusions

In this study, we described the design and pharmacological characterization of novel N/OFQ-derived peptide ligands obtained through a cumulative d-amino acid substitution strategy applied to the scaffold [Arg^14^ Lys^15^]­N/OFQ­(1-15)-NH_2_. Beyond the identification of a new bioactive analogue, this work defines the stereochemical tolerance of the NOP receptor toward multiple sequential d-amino acid substitutions within the address domain of the N/OFQ peptide, providing novel SAR information for the rational design of metabolically stable peptide agonists. This approach led to the identification of compound **3a**, which combines extensive chemical modification with preserved full agonist activity at the NOP receptor. Compound **3a** displayed high potency and selectivity *in vitro* and markedly prolonged NOP-mediated effects *in vivo*, inducing loss of the RR for up to 7 h after supraspinal administration. Notably, its *in vivo* pharmacological profile closely overlaps with that of the reference long-acting agonist UFP-112, despite distinct structural design strategies. Compound **3a** should be regarded as a proof-of-concept demonstrating the validity of the proposed stereochemical design strategy rather than as a replacement for UFP-112. Overall, these findings demonstrate that multiple d-amino acid incorporation into the N/OFQ peptide represents an effective alternative approach to generate long-acting NOP receptor agonists and further expand the medicinal chemistry toolbox available for peptide-based modulation of the NOP receptor.

## Materials and Methods

### Solid-Phase Peptide Synthesis

Peptide sequences were assembled by solid-phase peptide synthesis (SPPS) employing conventional Fmoc/tBu chemistry. Fmoc-protected amino acids and Rink Amide AmphiSpheres 20 RAM linker (loading 0.55 mmol/g; particle size 75–150 μm) were obtained from BLDpharm. The polystyrene-based resin Rink Amide was used as the solid support, allowing *C*-terminal amidation upon final cleavage. Automated synthesis was carried out on a SyroXP peptide synthesizer. The synthetic procedure consisted of iterative cycles including amino acid coupling, capping, and Fmoc group removal. Automated steps were performed using standardized solutions in DMF at fixed concentrations: HBTU (0.62 M), DIPEA (0.87 M), Ac_2_O (0.5 M), NMM (0.25 M), and piperidine (40%). Peptide cleavage from the resin was achieved using a trifluoroacetic acid-based mixture (TFA/triisopropylsilane/H_2_O, 95:2.5:2.5, 10 mL) for 4 hours at room temperature. The resin was removed by filtration, and the solution was concentrated under reduced pressure. Cold diethyl ether (−20 °C) was added to induce peptide precipitation. The solid products were subsequently collected by centrifugation and dried. Crude peptides were purified by reverse-phase semi-preparative 1260 Infinity II Preparative LC System (Agilent) equipped with a Jupiter C18 column (Phenomenex, 15 μm, 300 Å, 250 × 30 mm). Elution was performed at a flow rate of 30 mL/min, employing a linear gradient of solvent A (H_2_O with 0.1% TFA) and solvent B (40% H_2_O, 60% CH_3_CN, 0.1% TFA). The gradient conditions were optimized according to the analytical HPLC profile of each crude sample. Molecular weights of intermediates and final peptides were verified by electrospray ionization mass spectrometry (ESI-MS) using a MICROMASS ZMD 2000 instrument. Final compound purity was assessed by analytical reverse-phase HPLC on an Agilent 1200 series system equipped with UV detection. Analyses were performed on a Kinetex C18 column (Phenomenex, 5 μm, 100 Å, 150 × 4.6 mm) using a binary mobile phase composed of solvent A (H_2_O with 0.1% TFA) and solvent B (CH_3_CN with 0.1% TFA). A linear gradient from 100% A to 100% B over 25 minutes was applied. All purified peptides showed a purity higher than 95% by HPLC (see Supporting Information for chromatograms). High-resolution mass measurements for compound **3a** were obtained using an OptaMax NGH-ESI source coupled to a high-resolution Q-Orbitrap Exploris 240 mass spectrometer (see SI).

### Calcium Mobilization Assay

Chinese Hamster Ovary (CHO) cells stably co-expressing the human NOP, mu, or kappa opioid receptors and the Gα_qi5_ protein, or the human delta and the Gα_qG66Di5_ protein, were used in this assay.^24,48^ Cells were cultured in DMEM/F-12 (1:1) medium supplemented with 10% FBS, 2 mM l-glutamine, 200 μg/ml G418, 100 μg/ml Hygromycin B, 100 IU/ml penicillin, and 100 IU/ml streptomycin. Cells were maintained at 37 °C in a humidified atmosphere with 5% CO_2_ and were seeded at 50,000 cells/well into 96-well black, clear-bottom plates 24 h prior to testing. Loading for 45 min with a solution consisting of HBSS supplemented with 2.5 mM probenecid, 3 μM Fluo-4 AM, and 0.01% pluronic acid ensured the calcium-sensitive dye (Fluo-4) reached the required concentration inside the cells, while the exchange of the solution with 100 μL/well of buffer consisting of HBSS with 20 mM HEPES, 2.5 mM probenecid, and 500 μM Brilliant Black allowed the fluorescence background to decrease. Serial dilutions of ligands were prepared in HBSS buffer with 20 mM HEPES and 0.02% bovine serum albumin (BSA) to minimize ligands’ stickiness to plasticware. The automated microplate reader FlexStation II (Molecular Devices, CA, US) was employed at 37 °C to detect changes in fluorescence intensity. The effects of all compounds were expressed as the maximum change in percentage over the baseline fluorescence, measured in samples treated with vehicle.

### NOP Receptor–G Protein Interaction

A BRET interaction assay was used to study the propensity of ligands to evoke interaction between the NOP receptor and G protein.^25,49^ Membranes taken from HEK293 cells stably co-expressing the fusoproteins NOP-RLuc and Gβ1-RGFP were used. Such cells were grown in DMEM medium supplemented with 10% FBS, 2 mM l-Glutamine, 200 μg/ml G418, 100 μg/ml Hygromycin B, 100 IU/ml penicillin, and 100 IU/ml streptomycin in a humidified atmosphere with 5% CO_2_ at 37 °C. Cell membranes were thawed and resuspended in PBS supplemented with 0.01% BSA before the assay, and an amount of 3 μg of total protein was dispensed in each of the 96 wells together with 2 μM Prolume Purple Coelenterazine. All experiments were carried out at room temperature. Ligands were added, and averaged BRET ratios from 10 min measurements were computed. BRET ratios were computed as counts per second (CPS) obtained on a Victor Nivo (PerkinElmer) with 405 (10) nm and 510 (30) nm bandpass filters. BRET ratios were derived from light passed through the 510 nm filter divided by that passed through the 405 nm filter . Vehicle BRET values were subtracted from all computations, and datasets were normalized as a fraction of N/OFQ maximal effects. In antagonism experiments, a 10-min preincubation with vehicle or antagonist preceded the injection of the agonist.

### Mouse Vas Deferens Bioassay

This study was approved by the Animal Welfare Body of the University of Ferrara and by the Italian Ministry of Health (authorization number CBCC2.N.BXI). Experiments were performed on isolated mouse vas deferens. All experiments were conducted with mice bred and housed in the University of Ferrara’s animal facility under specific pathogen-free conditions (SPF). All mice were housed in cages with individual ventilation, with a constant temperature of 21 °C, 60% humidity, and a 12-hour light/dark cycle. Food and water were provided ad libitum. CD-1 NOP­(+/+) and CD-1 NOP­(−/−) male mice, aged 9 to 12 months, were used. Animals were sacrificed on the day of the experiment with CO_2_ overdose. Bioassay experiments were performed as previously described. The tissues were suspended in 5 mL organ baths containing Krebs solution (NaCl 118.5 mM, KCl 4.7 mM, KH_2_PO_4_ 1.2 mM, NaHCO_3_ 25 mM, CaCl_2_ 2.5 mM, and glucose 10 mM). The Krebs solution was oxygenated with 95% O_2_ and 5% CO_2_, and temperature set at 33 °C with a resting tension of 0.3 g applied to the tissues. Tissues were stimulated through two platinum electrodes with supramaximal rectangular pulses of 1 msec duration, 0.05 Hz frequency, and 80 V amplitude. The electrically evoked contractions were measured isotonically by means of Basile strain gauge transducers (Basile, IT) and recorded with Power Lab 8 (ADInstruments, CO, US). Following an equilibration period of approximately 60 min, the contractions induced by electrical field stimulation were stable. At this time, cumulative concentration–response curves were carried out.

### Loss of the Righting Reflex Assay

All the experimental procedures adopted in the *in vivo* studies comply with the European Directive 2010/63/EU on protecting animals used for scientific purposes and Italian Legislative Decree no. 26 of 4 March 2014. These experiments have been approved by the Animal Welfare Body of the University of Ferrara and by the Ministry of Health (authorization number 677/2024-PR). *In vivo* studies have been reported following ARRIVE guidelines.^50^ All experiments were conducted with mice bred and housed in the University of Ferrara’s animal facility under SPF conditions. All mice were housed in cages with individual ventilation, with a constant temperature of 21 °C, 60% humidity, and a 12-hour light/dark cycle. Food and water were provided ad libitum. CD-1 male and female mice, aged 2 to 4 months, were used. Details about the generation of NOP­(−/−) and NOP­(+/+) mice have been published previously;^51,52^ these mice have been backcrossed onto the CD-1 strain in our laboratories. NOP­(+/+) and NOP­(−/−) littermates were obtained by mating NOP­(+/−) mice. All mice were genotyped using the polymerase chain reaction as previously described.^53^ All mice were used only once. The RR assay was performed as previously described.^54^ Mice were given an i.c.v. injection of saline, N/OFQ (10 nmol), UFP-112 (0.1–1 nmol), or compound **3a** (0.1 – 1 nmol). I.c.v. injections (2 *μ*L per mouse) were administered under light isoflurane anesthesia into the left ventricle according to the procedure described by Laursen and Belknap^55^ and routinely adopted in our laboratory.^54^ When the animals lost RR, they were placed in a plastic cage, and the time was recorded by an expert observer blind to drug treatments and/or genotype. Animals were judged to have regained the RR response when they could right themselves three times within 30 s. Sleeping time is defined as the amount of time between the loss and regaining of the RR; it was rounded to the nearest minute.

### Molecular Modeling

The three-dimensional structure of the N/OFQ-NOP receptor complex was retrieved from the Protein Data Bank (PDB ID: 8F7X). Protein preparation was performed in MOE.^56^ The Gi protein subunits were deleted to reduce the complexity of the analyzed system. The N/OFQ Ala^15^ residue missing in the Cryo-EM structure was modeled by superimposing the experimental structure onto a previously generated AlphaFold model.^30^ d-amino acids were introduced by exploiting the mutate function in the MOE Protein Builder tool (details of the computational workflow are provided in Supplementary Figure S10). System preparation was carried out with the CHARMM-GUI web server.^57^ The receptor was embedded in an 80 Å × 80 Å 1-palmitoyl-2-oleyl-sn-glycero-3-phosphocoline (POPC) bilayer, and its orientation with respect to the membrane was predicted via the PPM 2.0 web server,^58^ as implemented in the CHARMM-GUI interface. Each system was included in a cubic box (system size: ∼80 Å x 80 Å x 120 Å; full details in Supplementary Table 1), solvated using TIP3P water molecules, and neutralized and salted to 0.15 M NaCl. The MD input files were generated by choosing the CHARMM36m force field and setting the WYF parameters for cation-π interactions. System equilibration was carried out in compliance with validated protocols for membrane systems, and 100 ns of MD production were performed in isothermal-isobaric conditions (NPT) by setting the temperature to 310.15 K using a Langevin thermostat and maintaining the pressure at 1 atm with a Monte Carlo barostat. All the MD simulations were carried out on a hybrid CPU/GPU cluster equipped with two NVIDIA RTX 3070 and one NVIDIA RTX 3070 Ti GPUs using the OpenMM 7.7.0 engine.^59^ MD trajectory analysis was performed using the MDAnalysis Python library^60^ and an in-house customized tcl script based on the NAMD 2.10 mdenergy function implemented in VMD.^61,62^ Receptor–peptide interactions were analyzed using the Protein-Ligand Interaction Profiler (PLIP)^32^ and visualized in PyMOL open source.

### Data Analysis, Statistics, and Terminology

Concentration-response curves to agonists were analyzed by a four-parameter logistic nonlinear regression model: Effect = baseline + *(E*_max_ – baseline) / (1 + 10 ^ ((logEC_50_ – log­[ligand]) slope)). In BRET, the effects were normalized to that of N/OFQ-NH_2_ or [Arg^14^Lys^15^N/OFQ-NH_2_ (*E*_max_ = 1). Antagonist potency was expressed as pA_2_, assuming a competitive type of antagonism. pA_2_ was calculated using the following equation: pA_2_ = log­(CR-1) – log­[*A*], where CR is the ratio between agonist potency (EC_50_) in the presence and absence of antagonist, and [*A*] is the molar concentration of antagonist. Experimental data were expressed as mean ± SEM of at least 5 experiments. Potency values were expressed as mean and CL_95%_. GraphPad Prism 10.0 software was used for all analyses. *In vitro* *E*_max_ values were compared using CL_95%_, lack of overlap with the reference agonist CL_95%_ was considered indicative of reduced efficacy. The normality of latency (time to lose the righting reflex) data was assessed using the Shapiro–Wilk test. Since the data fulfilled the assumption of normality, latency was analyzed by one-way ANOVA followed by Dunnett’s multiple comparisons test. Sleep duration data were analyzed using the non-parametric Kruskal–Wallis test followed by Dunn’s multiple comparisons test because the experimental cutoff introduced a ceiling effect that prevented the use of parametric statistics. The terminology employed is consistent with the International Union of Basic and Clinical Pharmacology (IUPHAR) recommendations.^63,64^

## Supplementary Material









## Data Availability

PDB structures of initial peptide-receptor complexes are provided as Supplementary Files. MD trajectories and topologies, together with all input files and scripts required to reproduce the simulations, will be made publicly available upon manuscript acceptance at: https://doi.org/10.5281/zenodo.21221071.

## Notes

3D coordinates of the NOP receptor complexes with [Arg^14^ Lys^1^ ^5^]­N/OFQ­(1–15)-NH_2_ and **1a–1i** (PDB) (ZIP)
HRMS spectra of compound **3a** and HPLC traces of all synthesized peptides **1a–i**, **2a–h**, **3a-3d** (S13–S34). Molecular formula strings (CSV)
Figures S1–S7 (NOP–G protein interaction assay and calcium mobilization assay). Figure S8 (mouse vas deferens bioassay). Figure S9 (MD analysis of peptides **1g**–**1i**). Table S1 (system details for MD simulations ). Table S2 (average Cα RMSD and RMSF values computed during 100 ns of MD). Figure S10 (computational Workflow) (PDF)
MD trajectory visualization of the NOP receptor in complexes with peptides [Arg^14^ Lys^15^]­N/OFQ­(1–15)-NH_2_ and **1a**–**1i** (ZIP)

## Abbreviation

ANOVA: analysis of variance
BRET: bioluminescence resonance energy transfer
BSA: bovine serum albumin
cAMP: cyclic adenosine monophosphate
CHO: Chinese hamster ovary cells
CL95%: 95% confidence limits
CPS: counts per second
DMEM: Dulbecco’s Modified Eagle Medium
DPDPE: [d-Pen^2^ , d-Pen^5^]­enkephalin
FBS: fetal bovine serum
GPCR: G protein-coupled receptor
HBSS: Hanks’ Balanced Salt Solution
HEK293: human embryonic kidney 293 cells
i.c.v.: intracerebroventricular­(ly)
mVD: mouse vas deferens
N/OFQ: nociceptin/orphanin FQ
NOP: nociceptin/orphanin FQ peptide receptor
PBS: phosphate-buffered saline
RR: righting reflex
SEM: standard error of the mean
