# Supplementary material for: Design of New Potent, Selective, and Long-Acting NOP Receptor Agonists through Multiple Sequential d‑Amino Acid Substitutions of [Arg14 Lys15]N/OFQ(1–15)-NH2
Source: J Med Chem. 2026 Jul 23;69(15):18977–91. doi: 10.1021/acs.jmedchem.6c01281 (PMC13492370; doi:10.1021/acs.jmedchem.6c01281)
Supplement: Supplementary file 1 [file jm6c01281_si_003.pdf]

Supporting Information for

Design of new potent, selective, and long-acting NOP receptor agonists through multiple sequential D-amino acid substitutions of [Arg^{14} Lys^{15}]N/OFQ(1-15)-NH_2

Chiara Sturaro[1#], Alessandra Rizzo[2#], Erika Morrone[3], Erika Marzola[2], Pietro Pola[1], Alessia Frezza[1], Michela Argentieri[1], Federica Agosta,[2] Antonella Ciancetta[2], Delia Preti[2], Salvatore Pacifico[2], Valentina Albanese[2], Giulio Meneguzzo[2], Davide Malfacini[3], Chiara Ruzza[1,4*,] Girolamo Calò[3], and Remo Guerrini[2,4]

[1] *Department of Neuroscience and Rehabilitation, University of Ferrara, Via Luigi Borsari 46, 44121 Ferrara, Italy.*
[2] *Department of Chemical, Pharmaceutical and Agricultural Sciences, University of Ferrara, Via Luigi Borsari 46, 44121 Ferrara, Italy.*
[3]*Department of Pharmaceutical and Pharmacological Sciences, Section of Pharmacology, University of Padova, Largo Meneghetti, 2, 35131 Padova, Italy.*
[4]*Technopole of Ferrara, Laboratory for Advanced Therapies (LTTA), via Fossato di Mortara 70, 44121 Ferrara, Italy*

[#] These authors contributed equally to this work

* Corresponding author
Chiara Ruzza, Email: chiara.ruzza@unife.it

CONTENTS

	Pag.
Figure **S1**. NOP – G protein interaction assays	*S1*
Figure **S2**. Calcium mobilization assays	*S2*
Figure **S3**. [Arg^{14} Lys^{15}]N/OFQ(1-15)-NH_2 analogues **1a-i** in NOP – G protein interaction assays	*S3*
Figure **S4**. [Arg^{14} Lys^{15}]N/OFQ(1-15)-NH_2 analogues **1a-i** in calcium mobilization assays	*S4*
Figure **S5**. [Arg^{14} Lys^{15}]N/OFQ(1-15)-NH_2 analogues **2a-h** in NOP – G protein interaction assays	*S5*
Figure **S6**. [Arg^{14} Lys^{15}]N/OFQ(1-15)-NH_2 analogues **2a-h** in calcium mobilization assays	*S6*
Figure **S7**. UFP-101 in NOP – G protein interaction and calcium mobilization assays. Crc to N/OFQ in the absence and in the presence of UFP-101 and **3d**	*S7*
Figure **S8**. Mouse vas deferens bioassay	*S8*
Figure **S9**. MD Analysis of peptides **1g-1i**	*S9*
Table **S1**. Systems details for Molecular Dynamic Simulations	*S10*
Table **S2**. Average Cα RMSD and RMSF values computed during 100 ns of MD	*S11*
Figure **S10**. Computational Workflow	*S12*
Supplementary Video Caption and Details	*S13*
HRMS method, spectrum and isotopic composition of **3a**	*S14*
HPLC and ESI-MS traces of peptides **1a-i**, **2a-h**, **3a-d**	*S15-35*

Supplementary figures

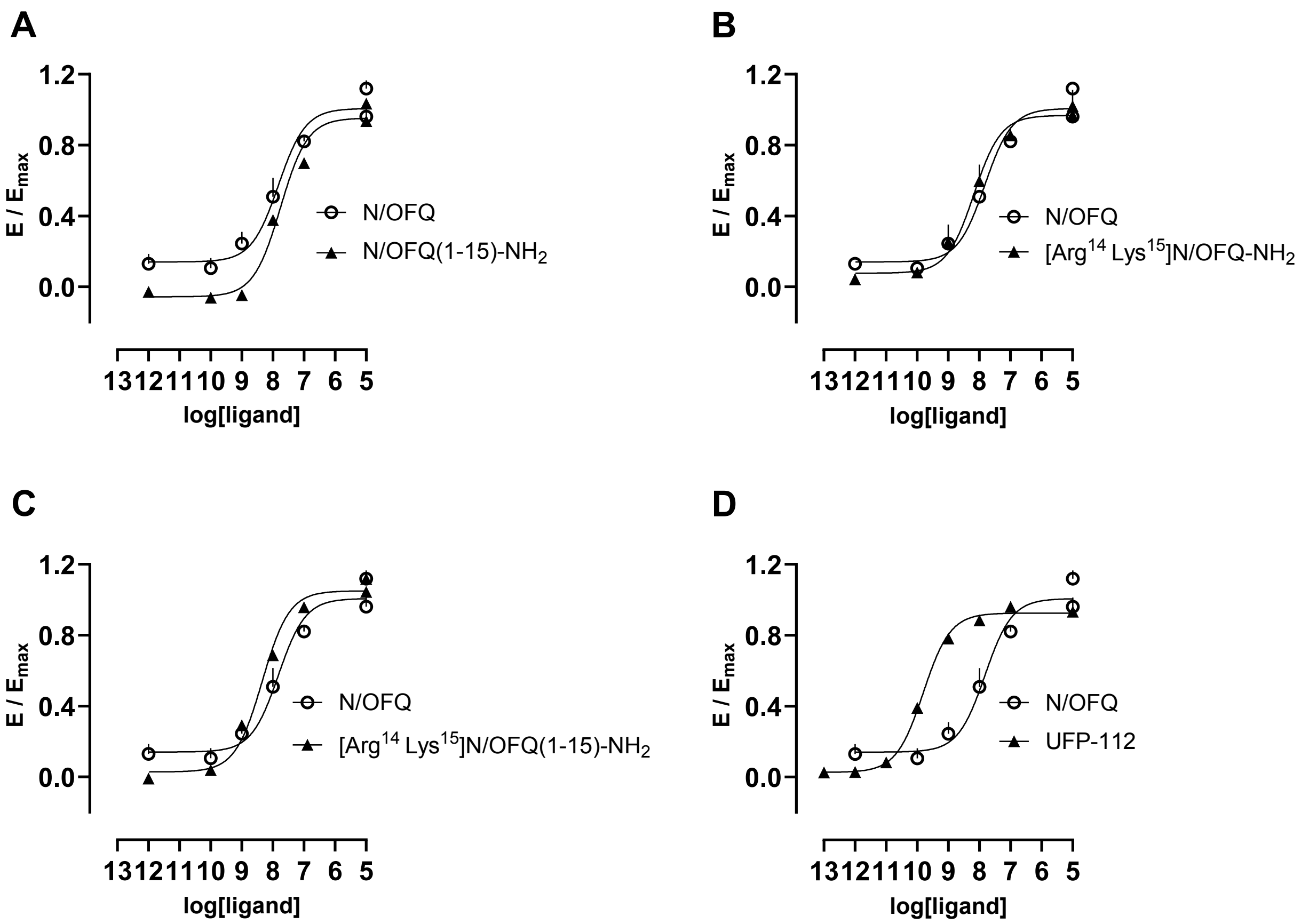

Supplementary Figure 1. In vitro pharmacological activity of N/OFQ, N/OFQ(1-15)-NH_2 (panel A), [Arg^{14} Lys^{15}]N/OFQ (panel B), [Arg^{14} Lys^{15}]N/OFQ(1-15)-NH_2 (panel C), and UFP-112 (panel D) in NOP – G protein interaction assays. Responses are expressed as E/E_{max} (E_{max} = maximal effect of N/OFQ. Data are the mean ± sem of 5 experiments conducted in duplicate.

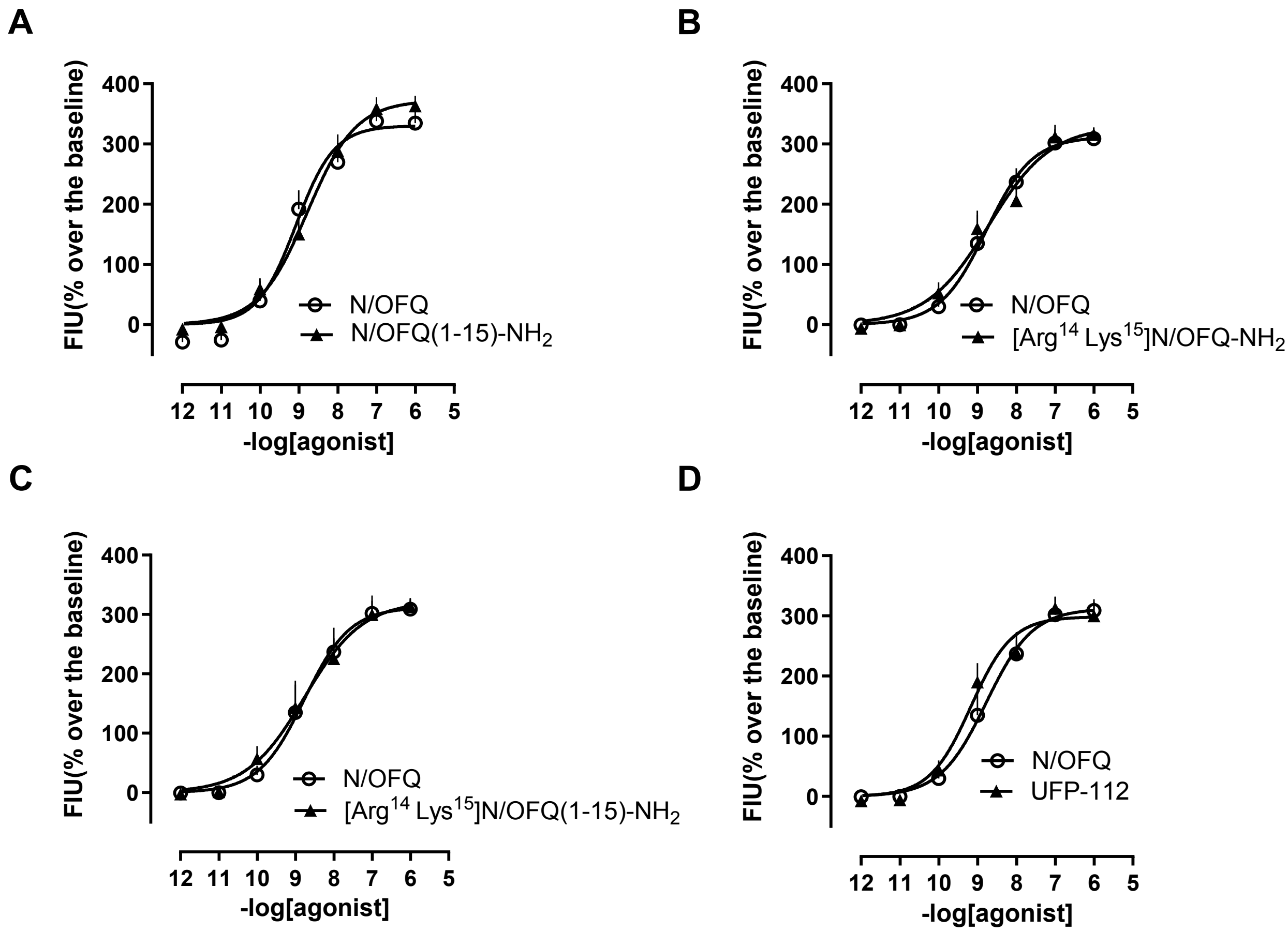

Supplementary Figure 2. In vitro pharmacological activity of N/OFQ, N/OFQ(1-15)-NH_2 (panel A), [Arg^{14} Lys^{15}]N/OFQ (panel B), [Arg^{14} Lys^{15}]N/OFQ(1-15)-NH_2 (panel C), and UFP-112 (panel D) in calcium mobilization and assays. Data are the mean ± sem of 5 experiments conducted in duplicate.

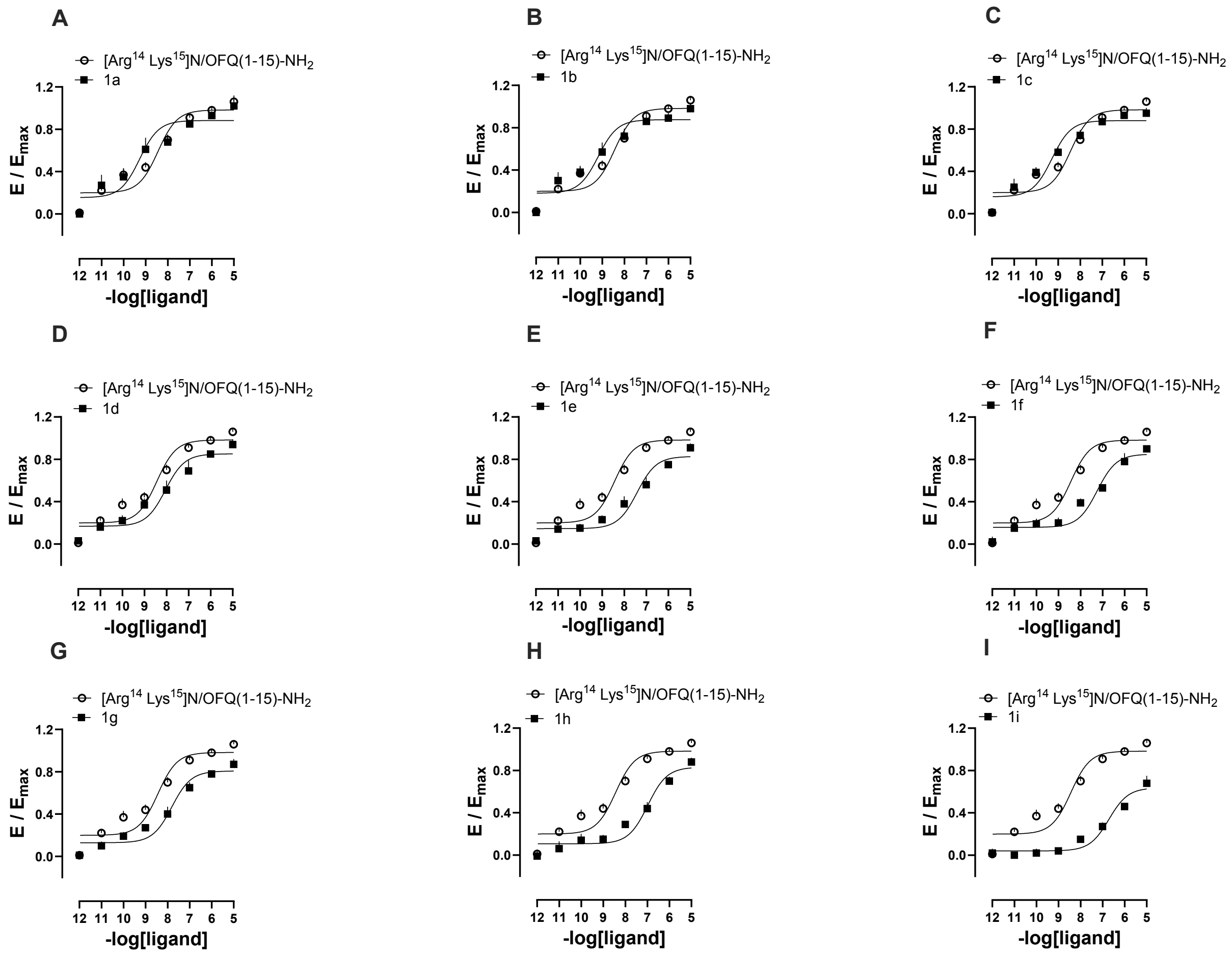

Supplementary Figure 3. In vitro pharmacological activity of [Arg^{14} Lys^{15}]N/OFQ(1-15)-NH_2 analogues with progressive D-amino acid substitutions from position 15 to 7 in NOP – G protein interaction assays. Panels A–I show the concentration–response curves of compounds 1a–1i, respectively. Responses are expressed as E/E_{max} (E_{max} = maximal effect of [Arg^{14} Lys^{15}]N/OFQ(1–15)-NH_2). Data are the mean ± sem of 5 experiments conducted in duplicate.

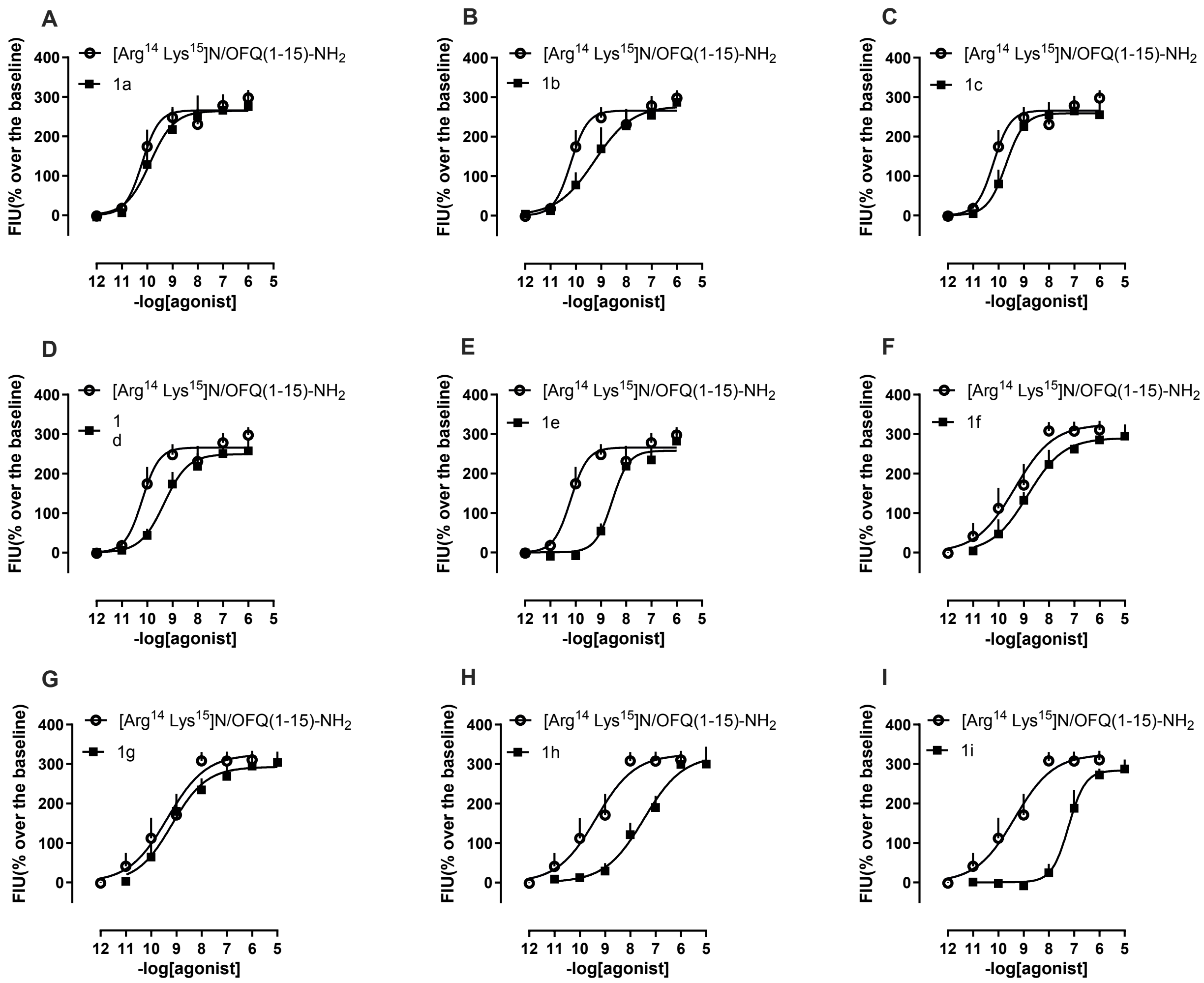

Supplementary Figure 4. In vitro pharmacological activity of [Arg^{14} Lys^{15}]N/OFQ(1-15)-NH$_2$ analogues with progressive D-amino acid substitutions from position 15 to 7 in calcium mobilization assays. Panels A–I show the concentration–response curves of compounds 1a–1i, respectively. Data are the mean ± sem of 5 experiments conducted in duplicate.

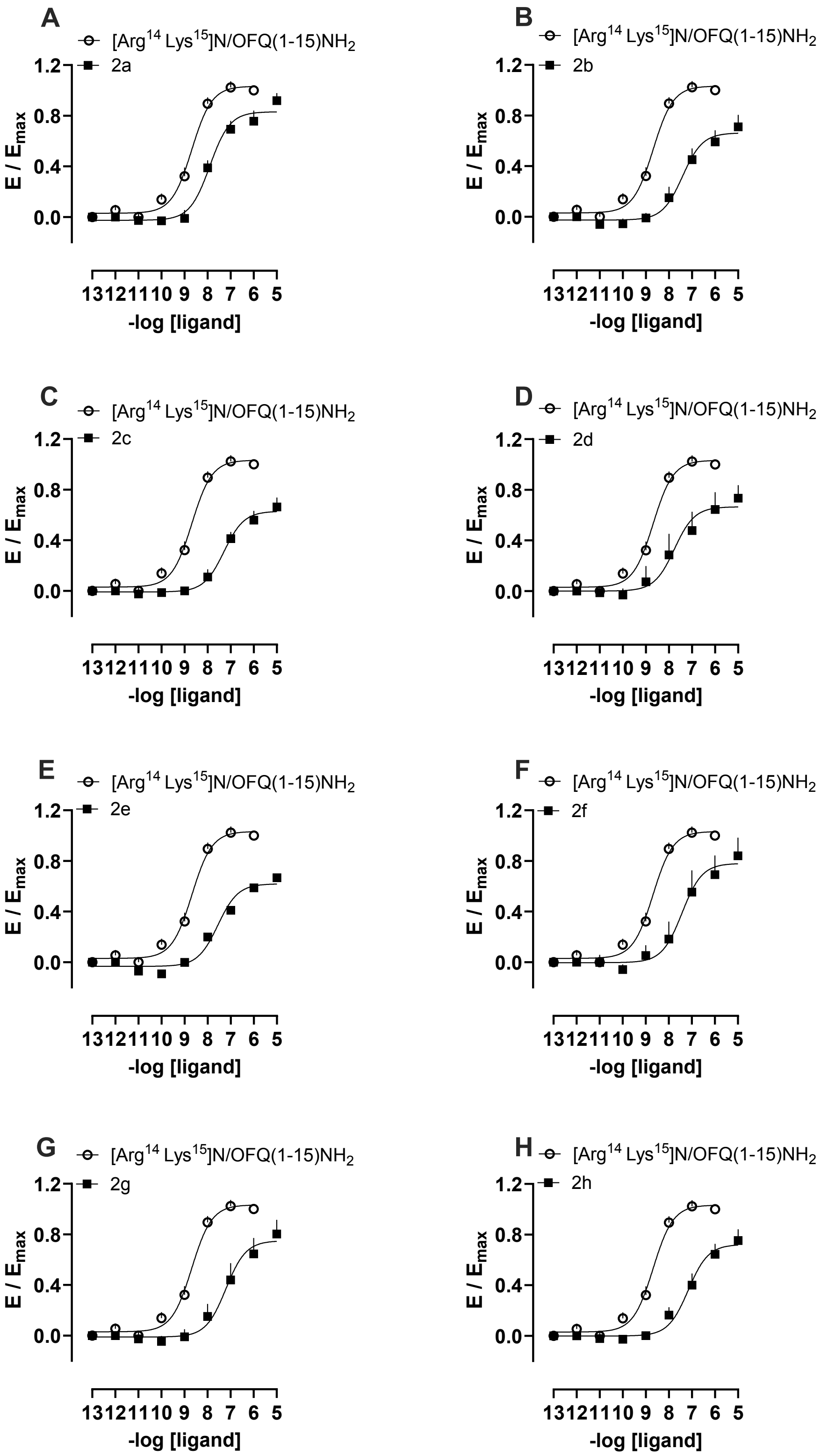

Supplementary Figure 5. In vitro pharmacological activity of [Arg[14] Lys[15]]N/OFQ(1-15)-NH_2 analogues with progressive D-amino acid substitutions from position 7 to 14 in NOP – G protein interaction assays. Panels A–H show the concentration–response curves of compounds 2a–2h, respectively. Responses are expressed as E/E_{max} (E_{max} = maximal effect of [Arg[14] Lys[15]]N/OFQ(1–15)-NH_2). Data are the mean ± sem of 5 experiments conducted in duplicate.

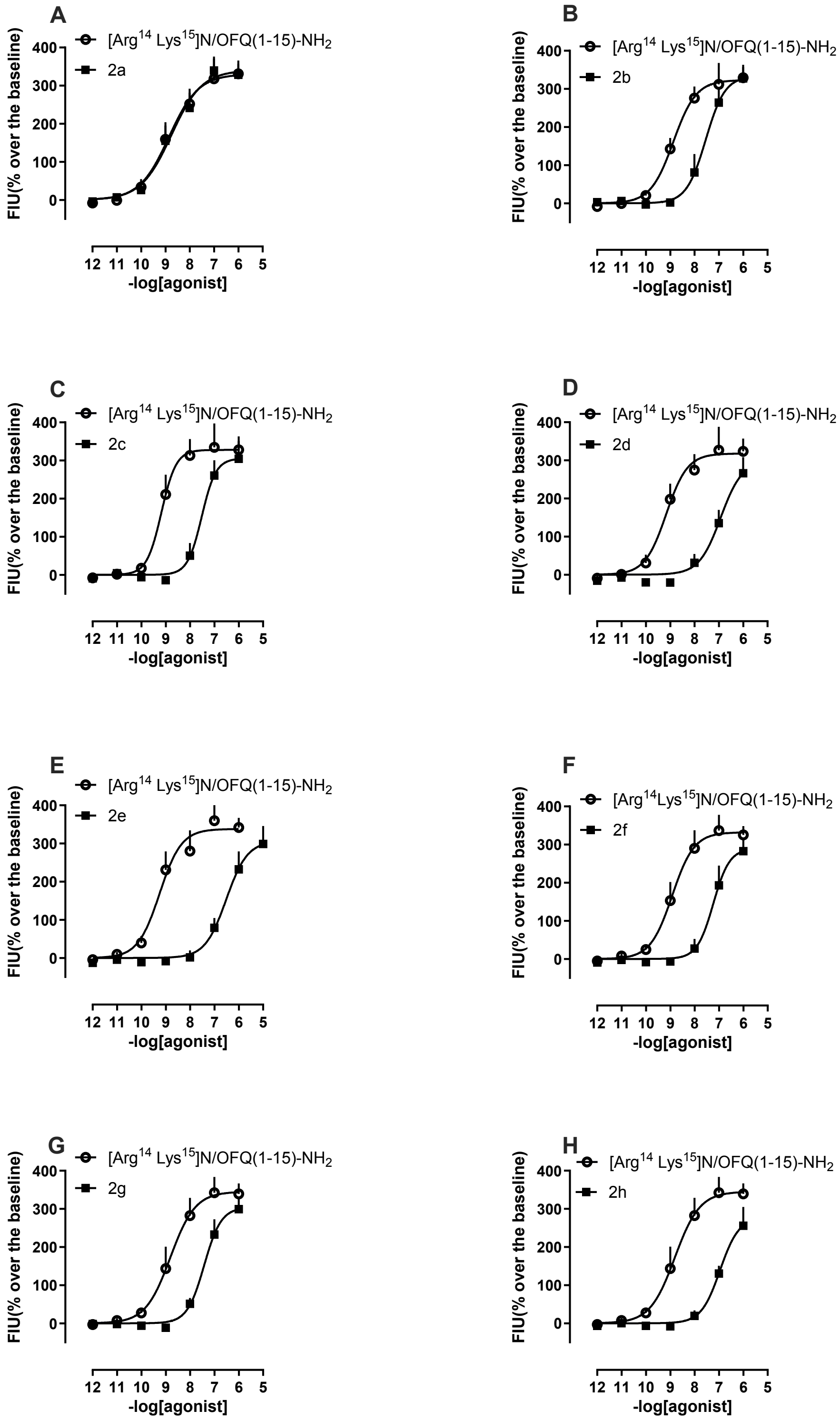

Supplementary Figure 6. In vitro pharmacological activity of [Arg^{14} Lys^{15}]N/OFQ(1-15)-NH_2 analogues with progressive D-amino acid substitutions from position 7 to 14 in calcium mobilization assays. Panels A–H show the concentration–response curves of compounds 2a–2h, respectively. Data are the mean ± sem of 5 experiments conducted in duplicate.

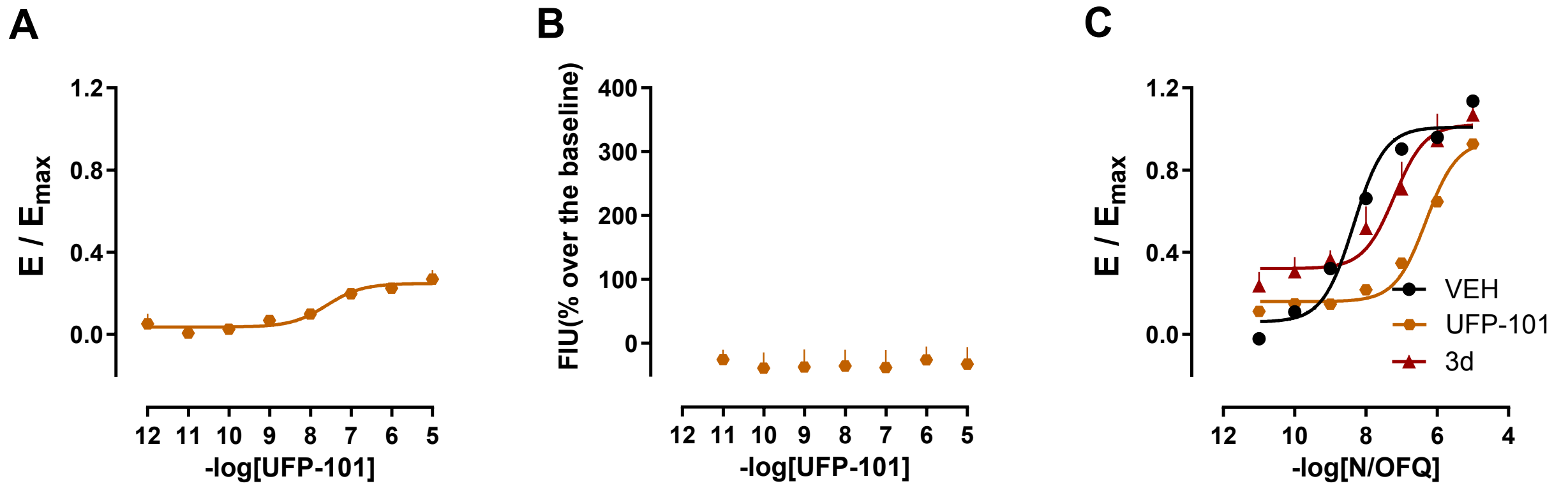

Supplementary Figure 7. In vitro pharmacological activity of UFP-101 in NOP – G protein interaction (panel A) and in calcium mobilization assays (panel B). Concentration-response curve to N/OFQ in the absence and in the presence of UFP-101 and compound 3d (panel C) in the NOP-G protein interaction assay. Panel C: UFP-101 and compound 3d 100 nM. In panels A and C, responses are expressed as E/E_{max} (E_{max} = maximal effect of [Arg^{14} Lys^{15}]N/OFQ(1–15)-NH_2). Data are the mean ± sem of 5 experiments conducted in duplicate.

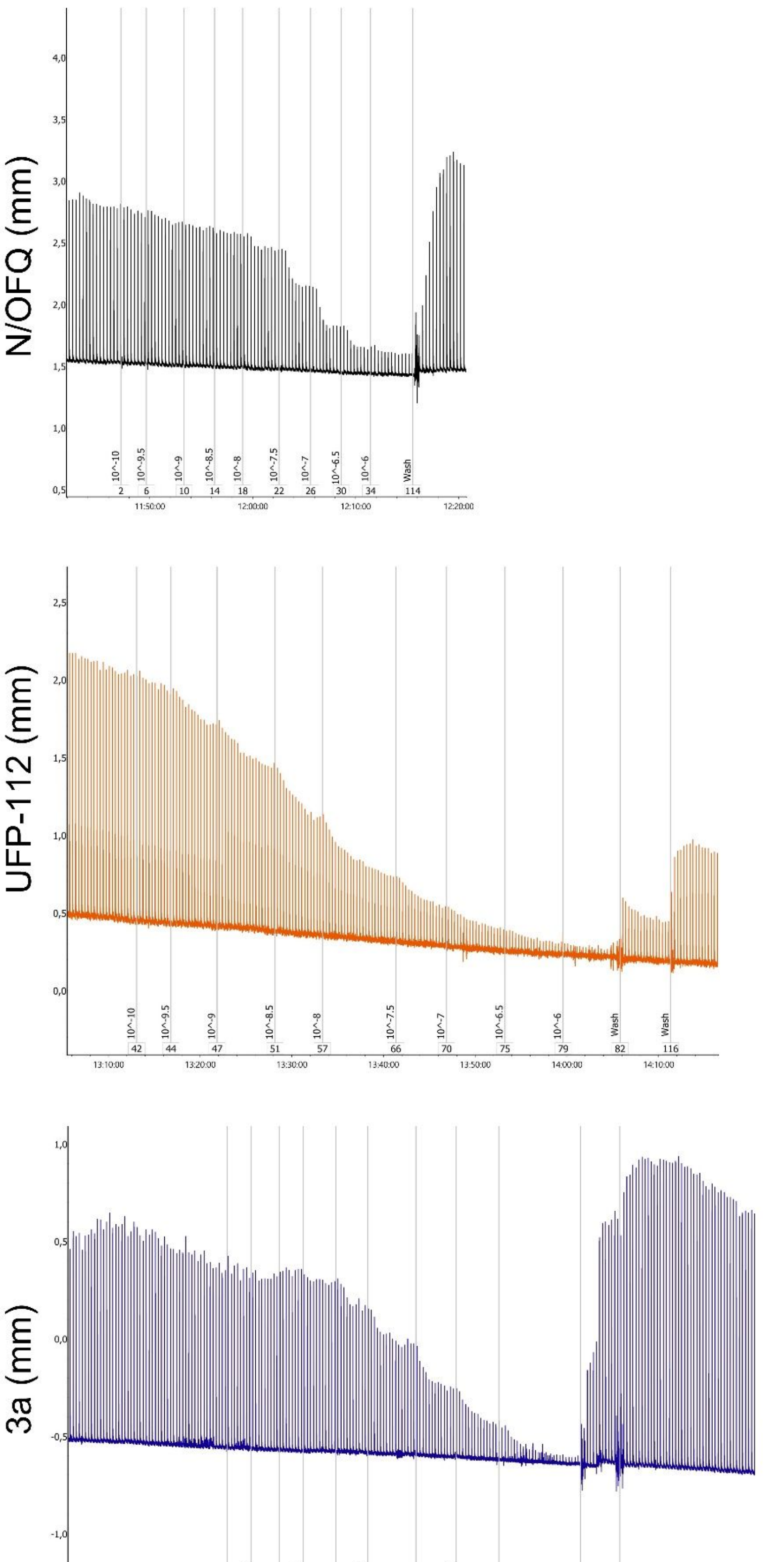

Supplementary Figure 8. Mouse vas deferens bioassay. Representative time-course recordings illustrating the onset and reversibility of the inhibitory effects induced by N/OFQ, UFP-112, and compound **3a** following washout.

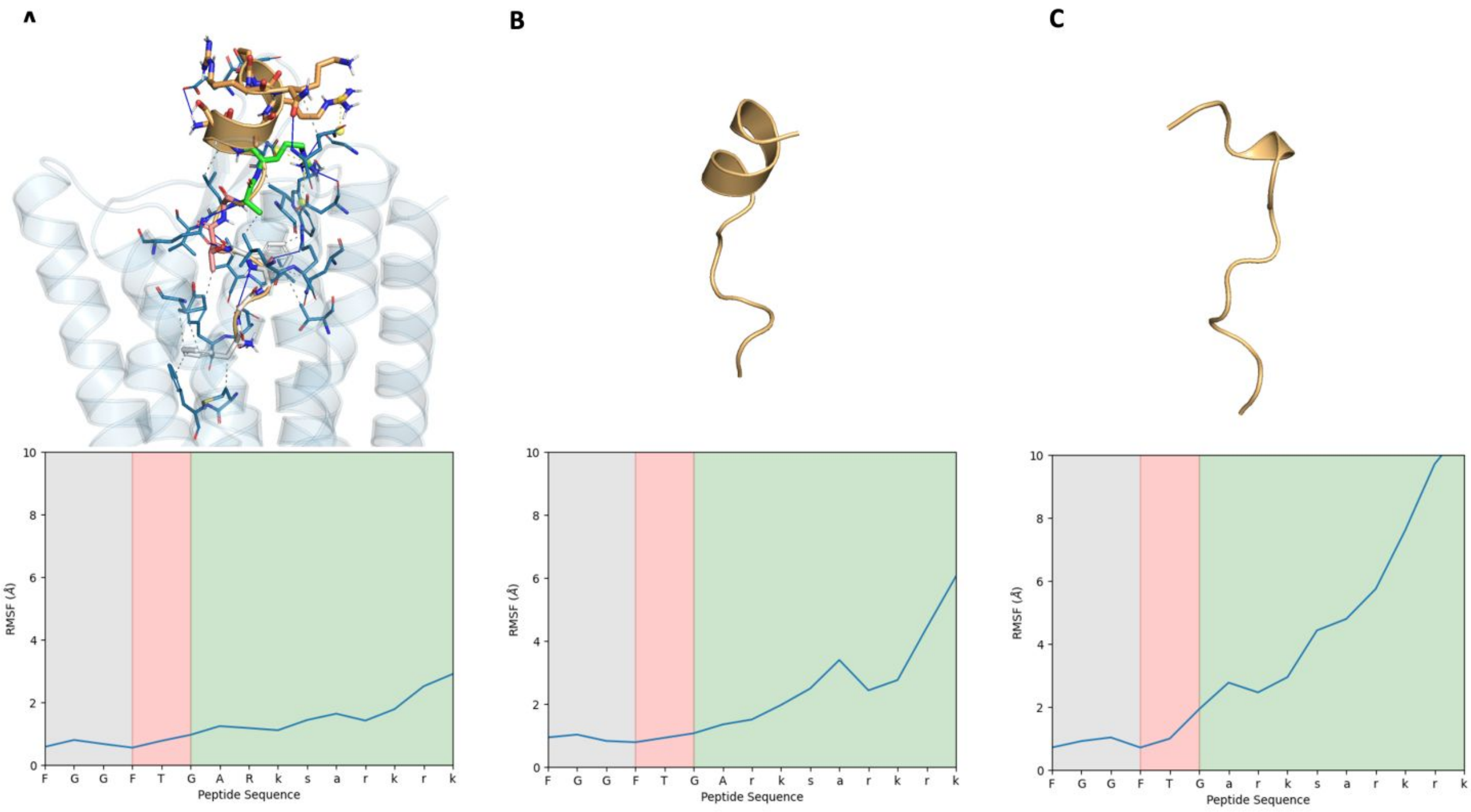

Supplementary Figure S9. **MD Analysis of peptides 1g-1i**. Top panels: peptide-receptor interaction pattern observed during the trajectory. Bottom panels: Peptide average RSMF profile. In the **1g** complex (A), D-Lys^{9} residue establishes an electrostatic interaction with E194^{ECL2}partially shared with D-Lys^{13}, while 1h (B) and **1i** (C) peptides are partially or totally unfolded with consequently increased peptide flexibility (as shown in the RMSF profile) and complex stability reduction. The initial complexes were generated using the Cryo-EM structure with PDB ID: 8F7X as template.

Supplementary Table S1. Systems details for Molecular Dynamic Simulations. NOP receptor (chain A) amino acid termini patches were set as GLYP and CT3, while the NTER and CT2 termini patches were applied to the peptides. The Disulfide bridge between Cys123 and Cys200 of Chain A was introduced into the topology file construction.

Protein-Peptide complex	Box Size			Number of Lipids		Number of Ions		Number of atoms	
Peptide sequence	X	Y	Z	Top	Bottom	Na^+	Cl^-	Waters	Total
H-FGGFTGARKSARKRK-NH_2	80.2	80.2	118.8	77	77	39	53	15068	70709
H-FGGFTGARKSARKRk-NH_2	80.2	80.2	118.8	77	77	39	53	15068	70707
H-FGGFTGARKSARKrk-NH_2	80.2	80.2	118.8	77	77	39	53	15092	70779
H-FGGFTGARKSARkrk-NH_2	80.2	80.2	119.3	77	77	40	54	15172	71019
H-FGGFTGARKSArkrk-NH_2	80.2	80.2	119.3	77	77	40	54	15161	70988
H-FGGFTGARKSarkrk-NH_2	80.2	80.2	119.3	77	77	40	54	15196	71093
H-FGGFTGARKsarkrk-NH_2	80.2	80.2	119.3	77	77	40	54	15176	71033
H-FGGFTGARksarkrk-NH_2	80.2	80.2	119.3	77	77	40	54	15173	71024
H-FGGFTGArksarkrk-NH_2	80.2	80.2	119.3	77	77	40	54	15162	70991
H-FGGFTGarksarkrk-NH_2	80.2	80.2	119.3	77	77	40	54	15152	70961

Supplementary Table 2. Average Cα Root Mean Square Deviation (RMSD) and the Root Mean Square fluctuation (RMSF) computed during a 100 ns MD trajectory for the solvated membrane-embedded peptide-NOP receptor complexes.

	RMSD		RMSF	
**System**	Receptor Cα	Peptide Cα	Receptor Cα	Peptide Cα
**Ref**	1.448±0.196	1.436±0.295	1.062±0.105	0.885±0.279
**1a**	1.431±0.195	1.402±0.249	0.984±0.137	0.877±0.275
**1b**	1.677±0.277	1.393±0.380	1.031±0.173	0.983±0.363
**1c**	1.891±0.271	2.380±0.411	1.210±0.162	1.184±0.326
**1d**	1.522±0.238	2.512±0.551	1.111±0.180	1.650±0.406
**1e**	1.629±0.223	2.801±1.072	1.091±0.191	1.639±1.005
**1f**	1.732±0.175	3.258±1.026	0.918±0.145	1.891±0.429
**1g**	1.667±0.211	2.045±0.960	1.010±0.181	1.368±0.476
**1h**	1.595±0.187	3.323±0.946	1.103±0.180	2.491±0.666
**1i**	1.581±0.196	6.027±1.198	1.381±0.155	4.865±1.244

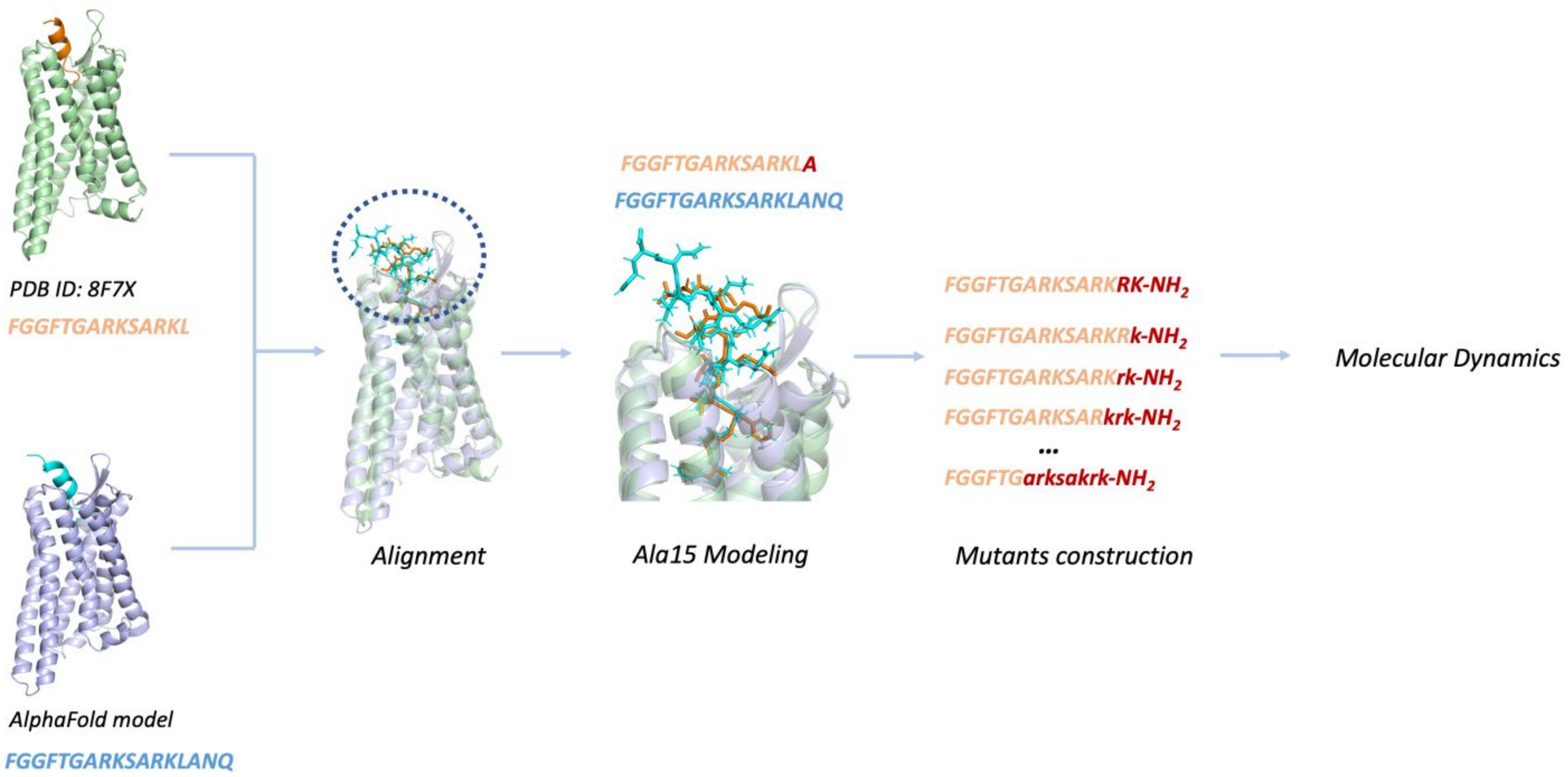

Supplementary Figure S10. **Computational Workflow.** The PDB structure (green model) was aligned to the AlfaFold in-house model (blue) to properly model the missing peptide Ala^{15} residue. D-peptides were generated through an in-silico mutation of native amino acids, and protein-peptide systems stability was analysed through molecular dynamics simulations.

Caption for Supplementary Videos. MD trajectory visualisation of the peptide–NOP receptor complex. The NOP receptor is shown as a green ribbon. The peptide is displayed as ribbons coloured according to functional regions: message (magenta), hinge (green), address (purple), and Arg^{14} and Lys^{15} residues (yellow). Receptor residues within 4 Å of the peptide are shown as lines with cyan carbon atoms. Hydrogen bonds are highlighted as orange dashed lines throughout the trajectory. Videos were rendered with VMD.

Video S1. MD trajectory visualisation of NOP receptor complex with of [Arg^{14} Lys^{15}]N/OFQ(1-15)-NH_2

Video S2. MD trajectory visualisation of NOP receptor complex with **1a**

Video S3. MD trajectory visualisation of NOP receptor complex with **1b**

Video S4. MD trajectory visualisation of NOP receptor complex with **1c**

Video S5. MD trajectory visualisation of NOP receptor complex with **1d**

Video S6. MD trajectory visualisation of NOP receptor complex with **1e**

Video S7. MD trajectory visualisation of NOP receptor complex with **1f**

Video S8. MD trajectory visualisation of NOP receptor complex with **1g**

Video S9. MD trajectory visualisation of NOP receptor complex with **1h**

Video S10. MD trajectory visualisation of NOP receptor complex with **1i**

HRMS Method

The identity of the synthesized peptide **3a** was confirmed by HRMS analysis. The dried peptide was reconstituted in LC–MS grade water containing 0.1% formic acid and diluted to obtain a final concentration of 5 ppm. The resulting solution was then introduced by direct infusion into an OptaMax NG H-ESI source coupled to a high-resolution Q-Orbitrap Exploris 240 mass spectrometer. The source parameters used for the analysis are reported in Tab S1.

***Tab S1**. H-ESI source and MS acquisition parameters*	
Spray Voltage	+3700 V
Sheath gas	50 a.u.
Aux Gas	3 a.u.
Sweep Gas	0 a.u
Ion Transfer Tube Temperature	320 °C
Vaporizer Temperature	0 °C
Siringe flow rate	5 µl/min
Scan Range	200-1700
Resolution	60,000 (at m/z 200)

A representative mass spectrum obtained from the analysis is shown in the following figure S9. As can be observed, the peptide was detected with multiple charge state distributions, with the +4 charge state being the most abundant. Significant signal intensities were also observed for the +5, +3, and +2 charge states. In Table S2, the monoisotopic m/z values for the different observed charge states of peptide **3a** are reported.

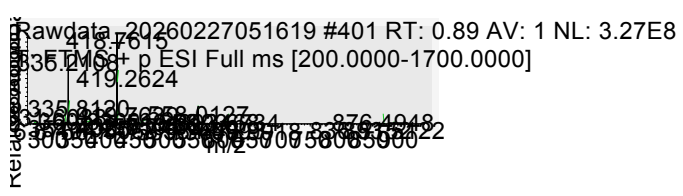

Supplementary Figure 11. Representative high-resolution mass spectrum of the synthesized peptide **3a**.

***Tab S3**. Comparison between theoretical and experimentally measured monoisotopic m/z values for the different observed charge states of the peptide. Mass accuracy is reported as absolute deviation (Δ, Da) and relative error (ppm).*					
**Found adducts with H^+**	**Theoretical monoisotopic m/z**	**Measured monoisotopic m/z**	**Δ (Da)**	**error (ppm)**	**Average isotopic spacing (m/z)**
$[M+2H]^{2+}$	836.5157	836.5152	−0.0005	−0.63	0.5014
$[M+3H]^{3+}$	558.0129	558.0127	-0.0002	-0.35	0.3341
$[M+4H]^{4+}$	418.7615	418.7615	0.0000	0	0.2505
$[M+5H]^{5+}$	335.2107	335.2108	0.0001	+0.43	0.2004

HPLC traces and ESI-MS spectra of final peptides 1a-i, 2a-h, 3a-d

*Compound **1a***

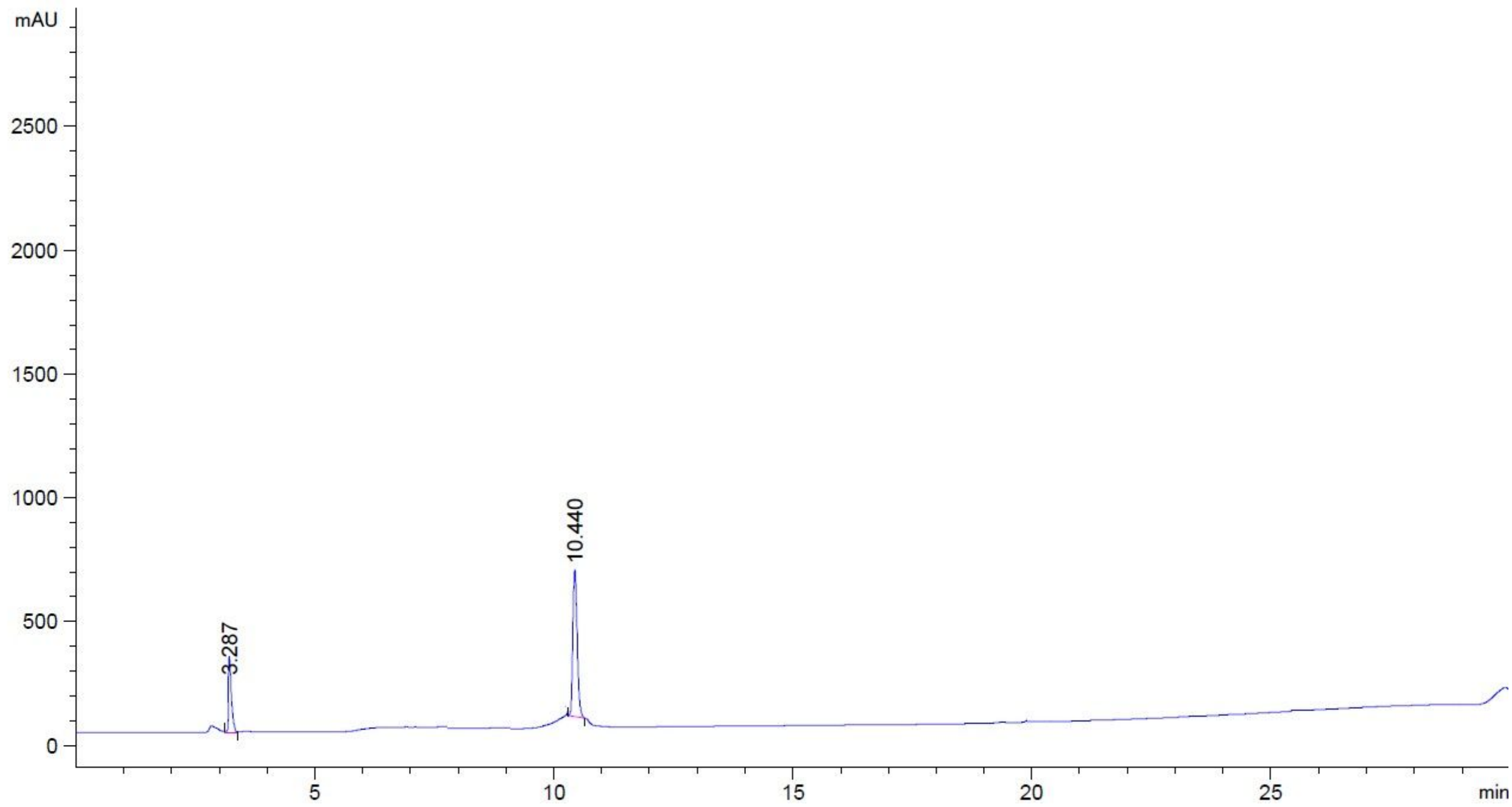

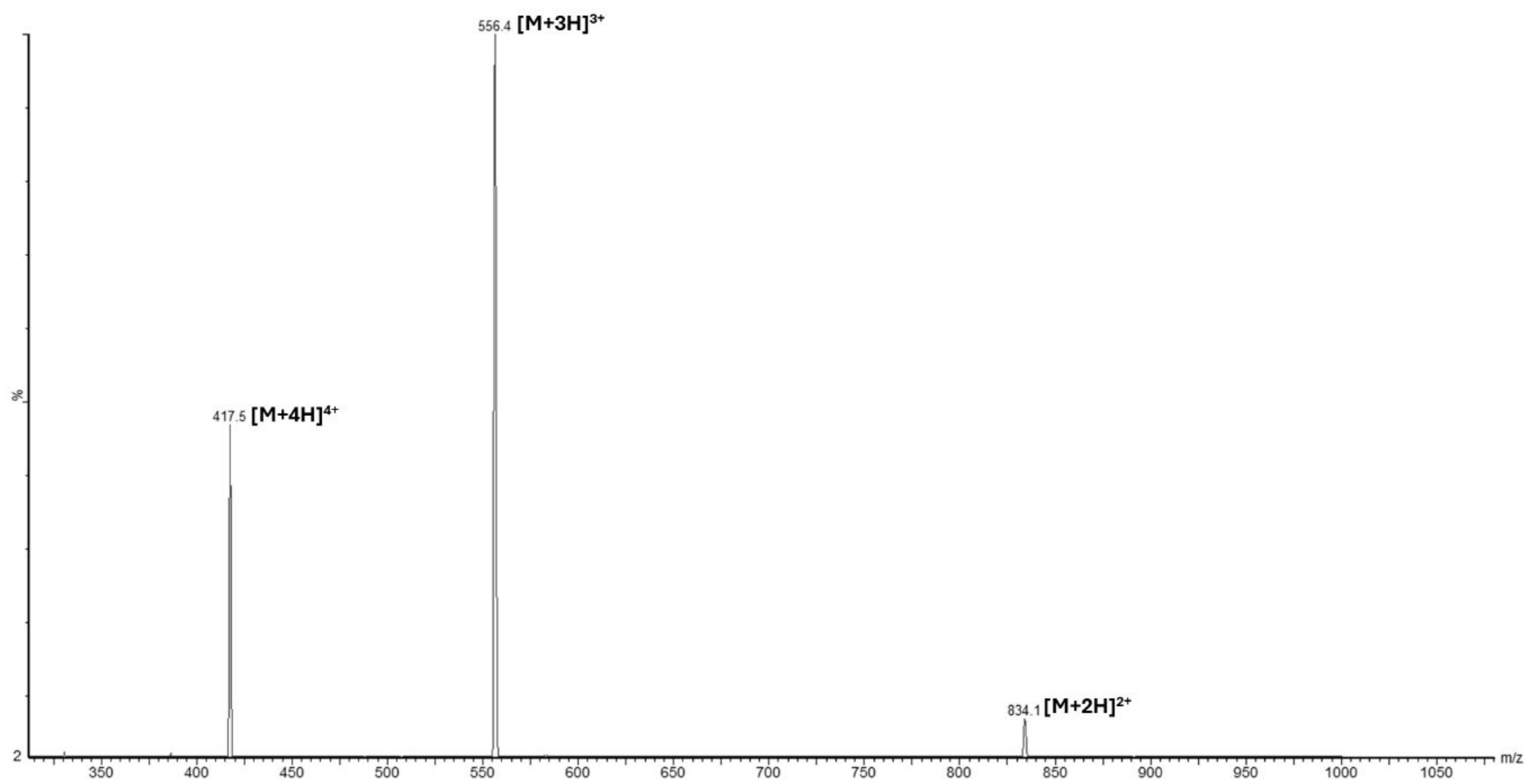

*Compound **1b***

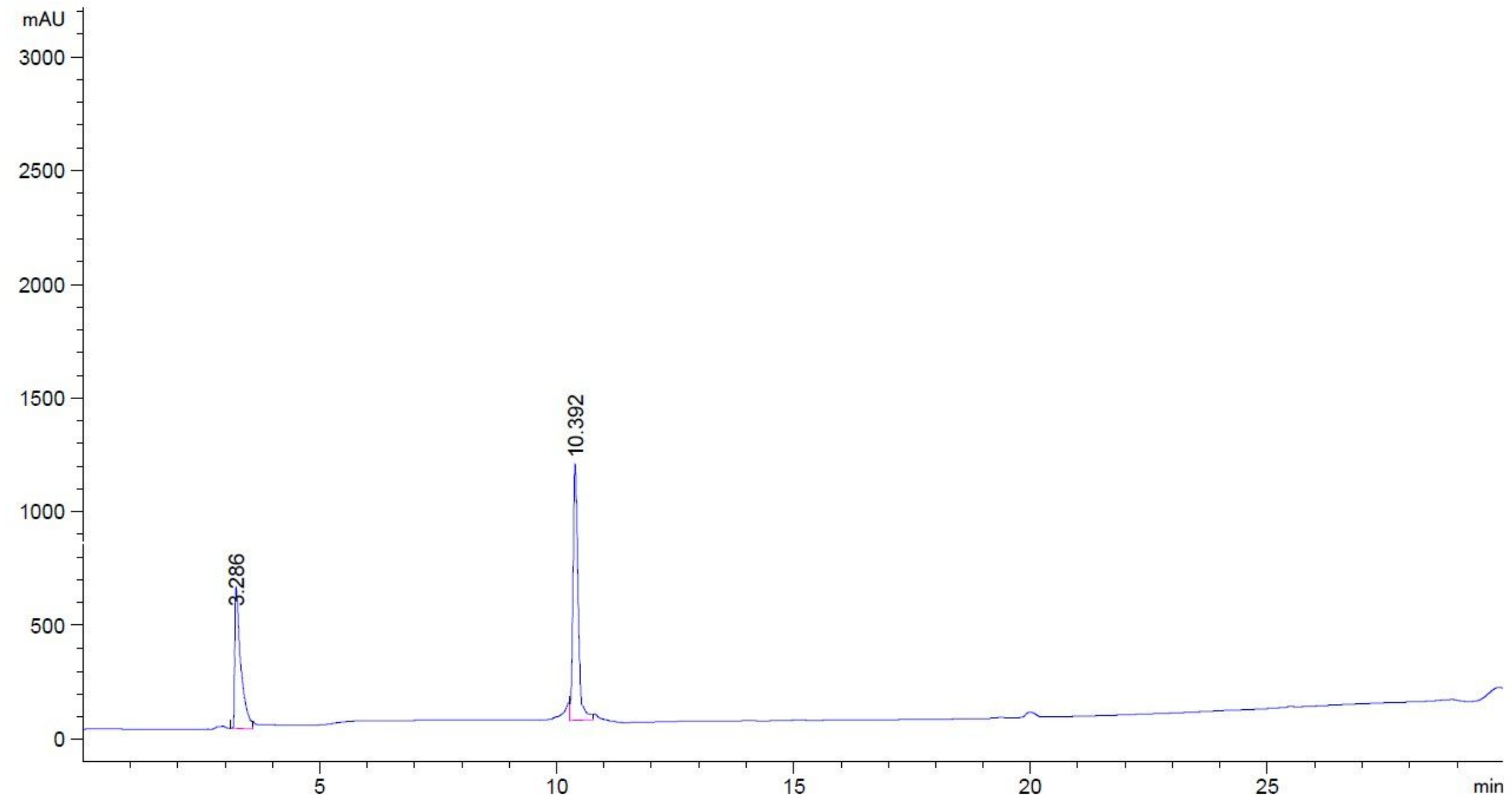
mAU
3000
2500
2000
1500
1000
500
0
3.286
10.392
5
10
15
20
25
min

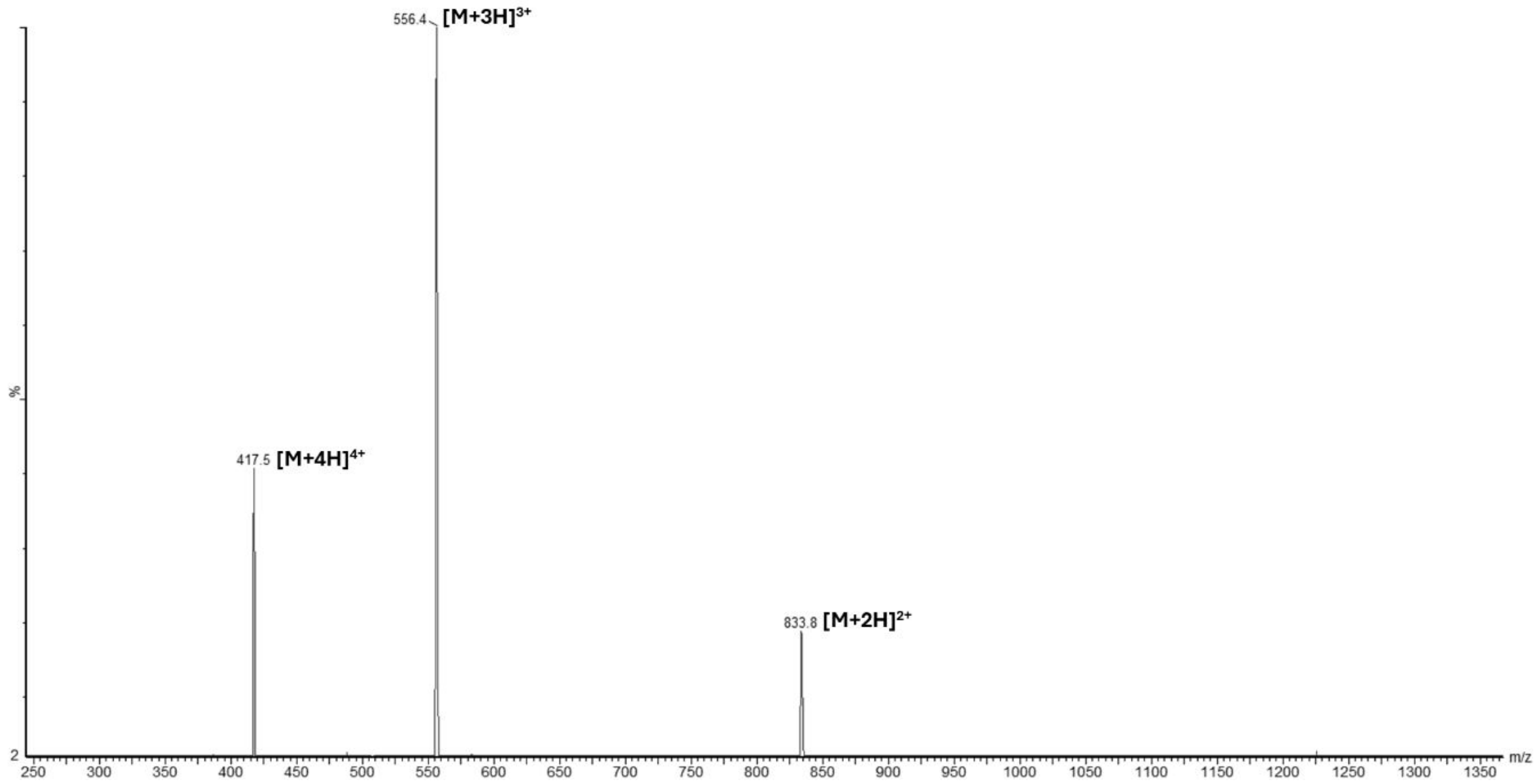
556.4 [M+3H]3+
417.5 [M+4H]4+
833.8 [M+2H]2+
%
m/z

*Compound **1c***

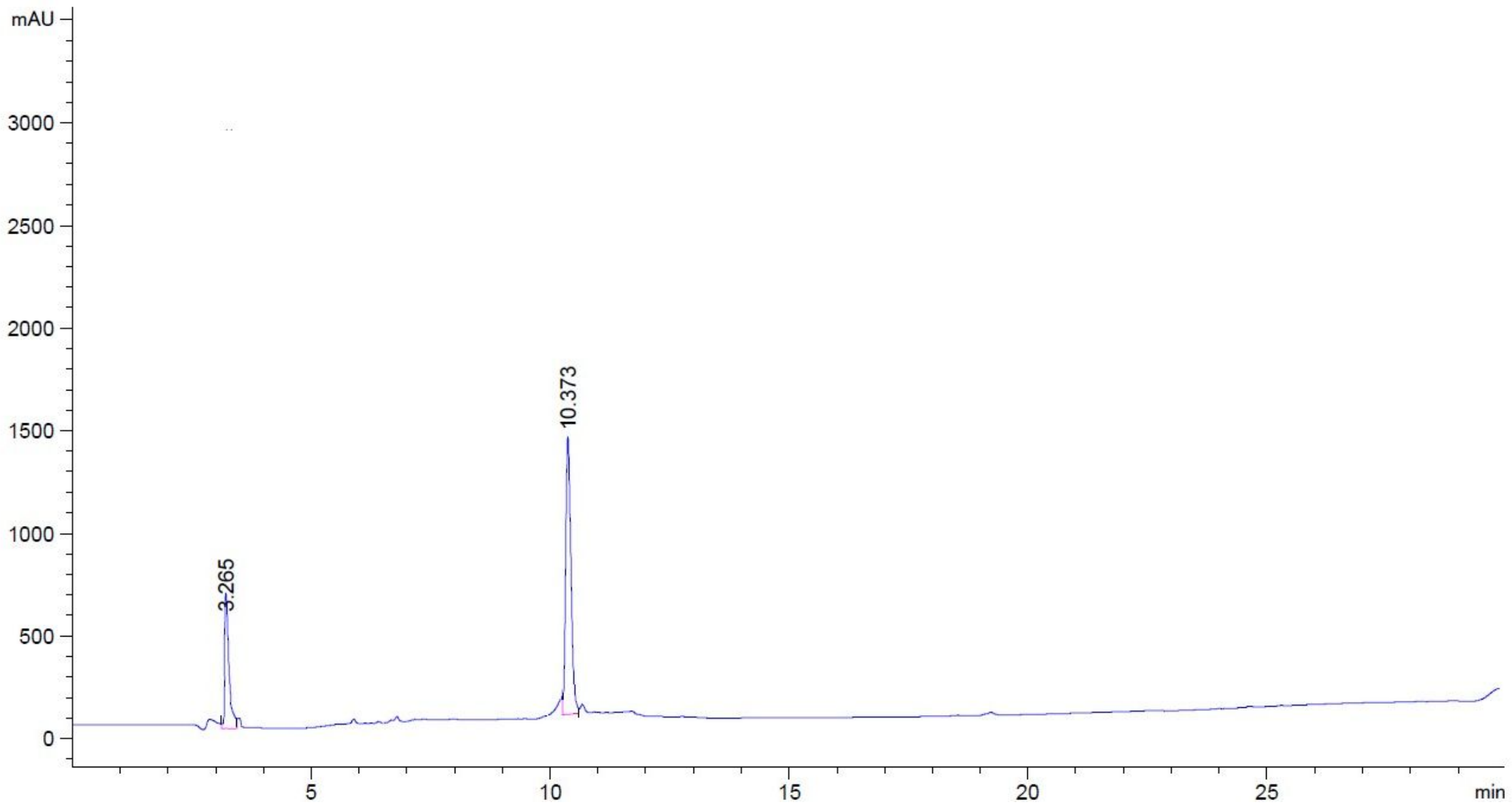
mAU
3000
2500
2000
1500
1000
500
0
10.373
3.265
5
10
15
20
25
min

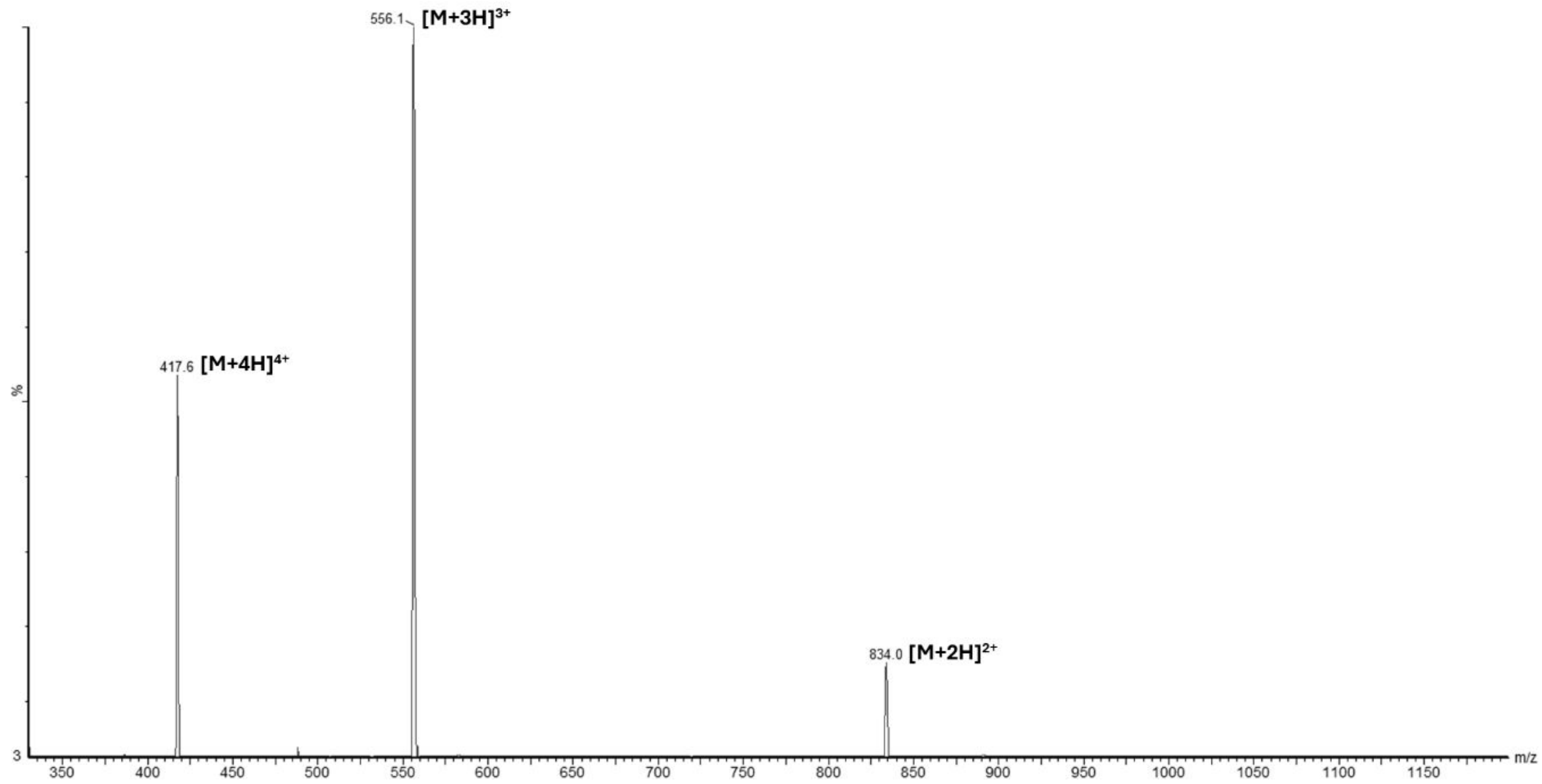
556.1 [M+3H]3+
417.6 [M+4H]4+
834.0 [M+2H]2+
%
3
350
400
450
500
550
600
650
700
750
800
850
900
950
1000
1050
1100
1150
m/z

*Compound **1d***

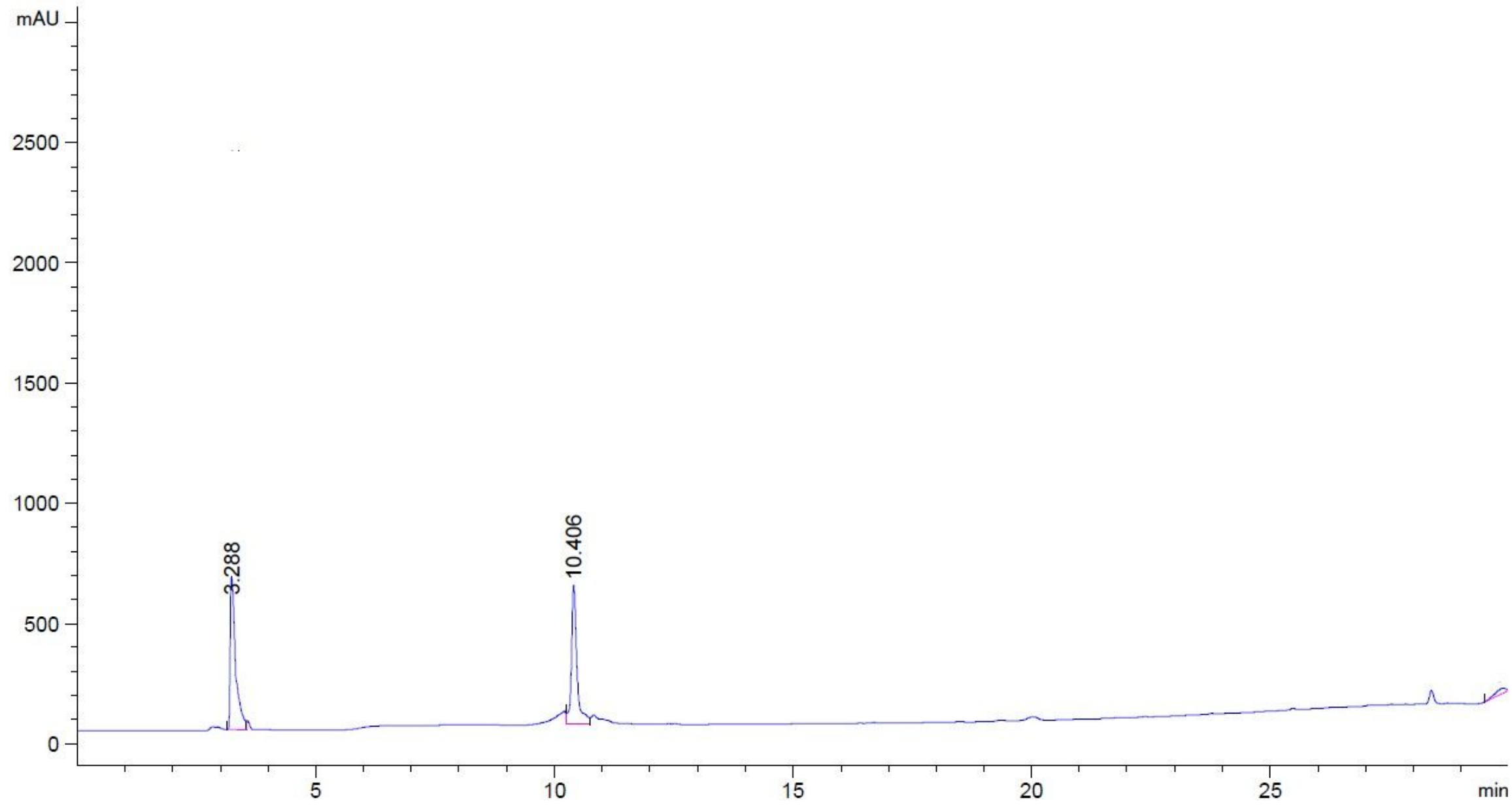
mAU
2500
2000
1500
1000
500
0
3.288
10.406
5
10
15
20
25
min

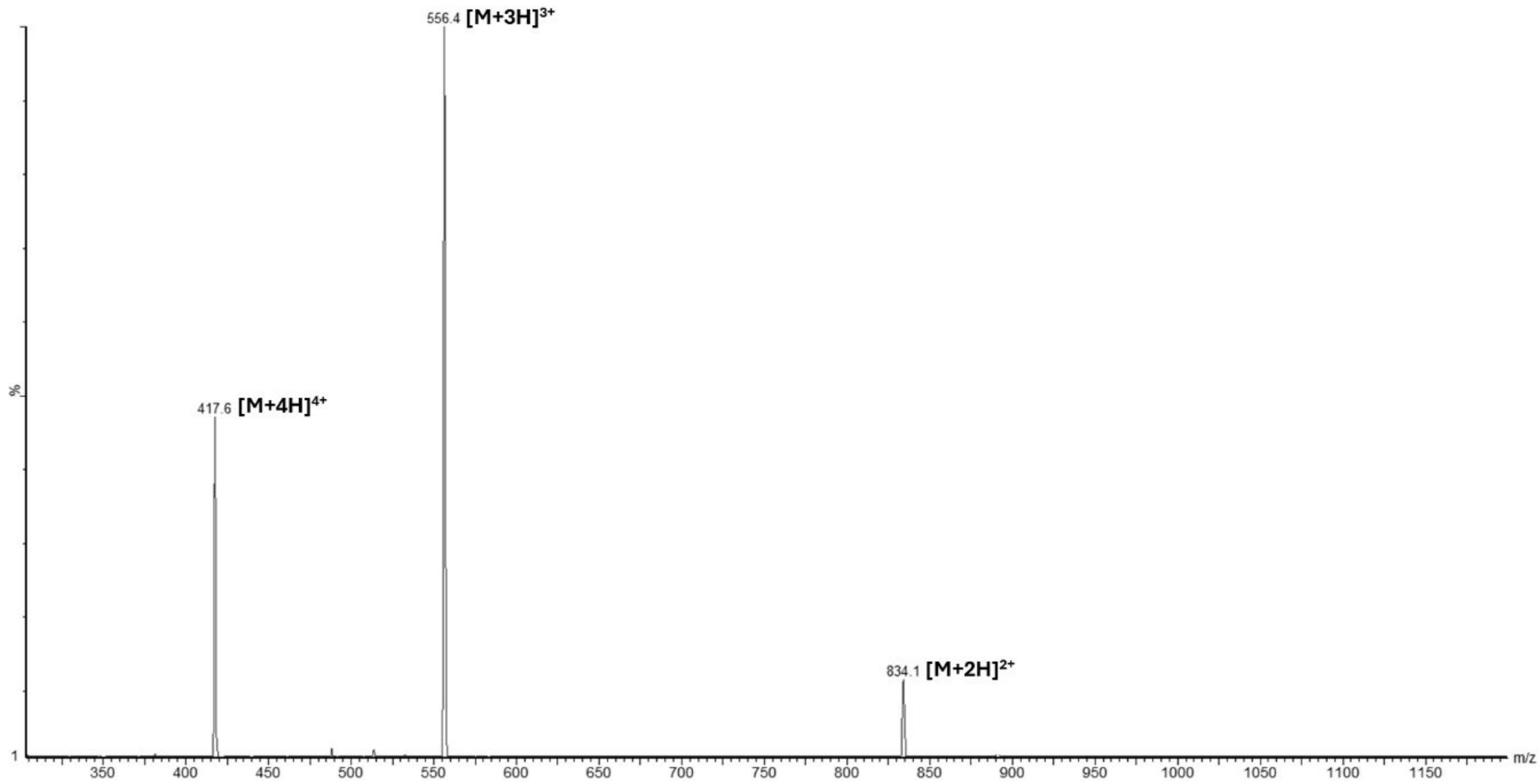
556.4 [M+3H]3+
417.6 [M+4H]4+
834.1 [M+2H]2+
%
1
350
400
450
500
550
600
650
700
750
800
850
900
950
1000
1050
1100
1150
m/z

Compound ***1e***

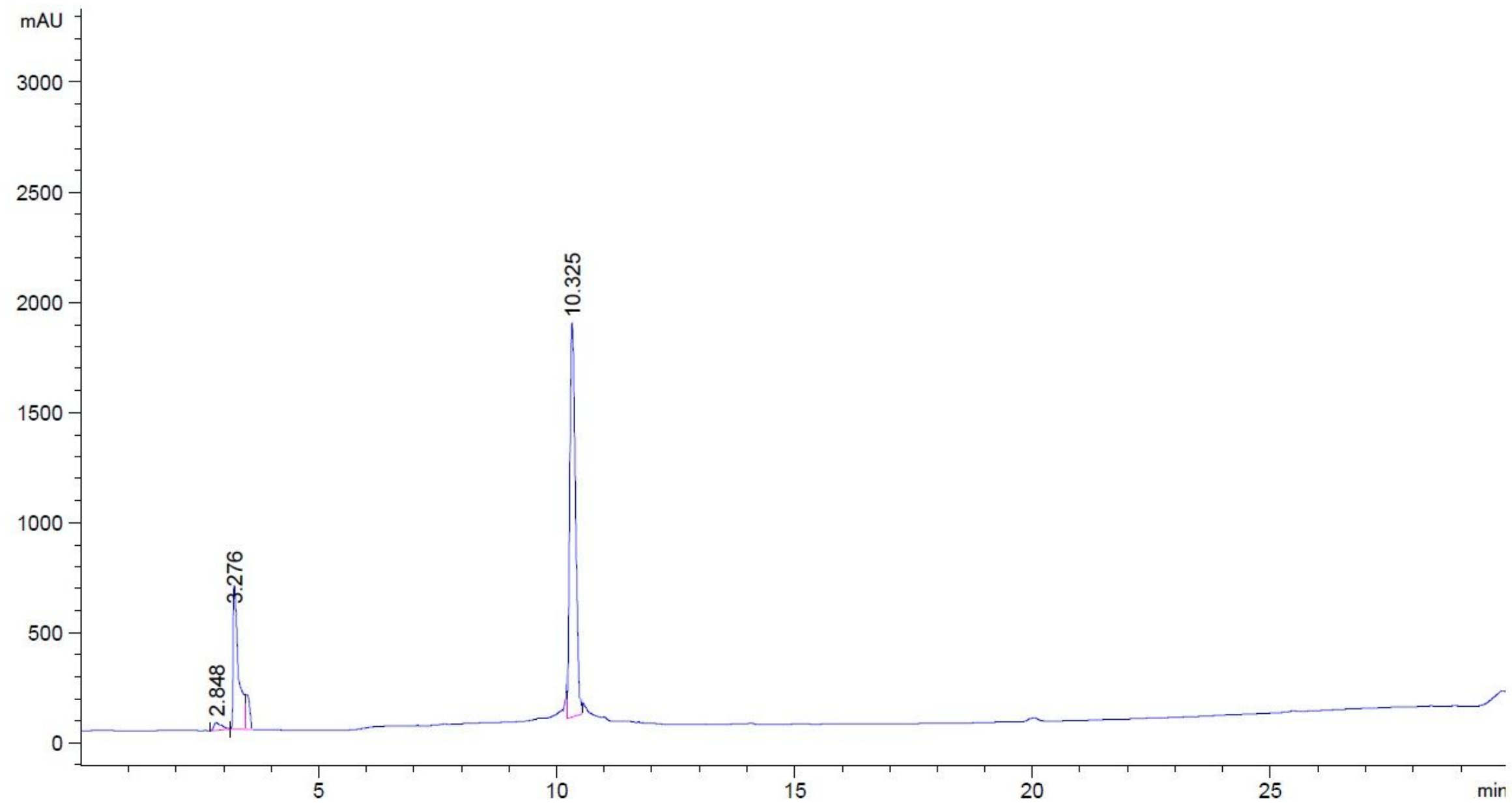

mAU
3000
2500
2000
1500
1000
500
0
10.325
3.276
2.848
5
10
15
20
25
min

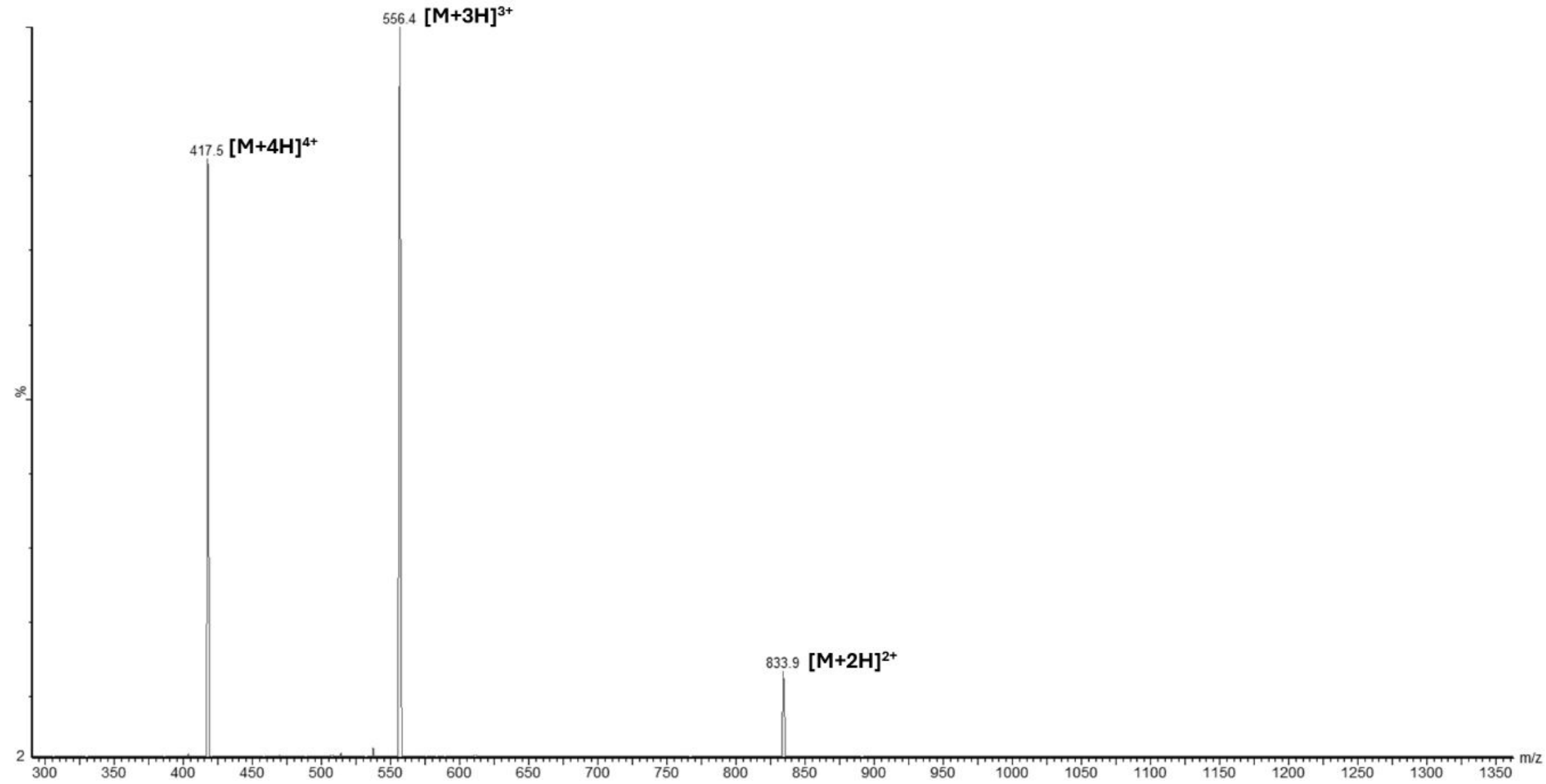

556.4 [M+3H]3+
417.5 [M+4H]4+
833.9 [M+2H]2+
%
2
300
350
400
450
500
550
600
650
700
750
800
850
900
950
1000
1050
1100
1150
1200
1250
1300
1350
m/z

*Compound **1f***

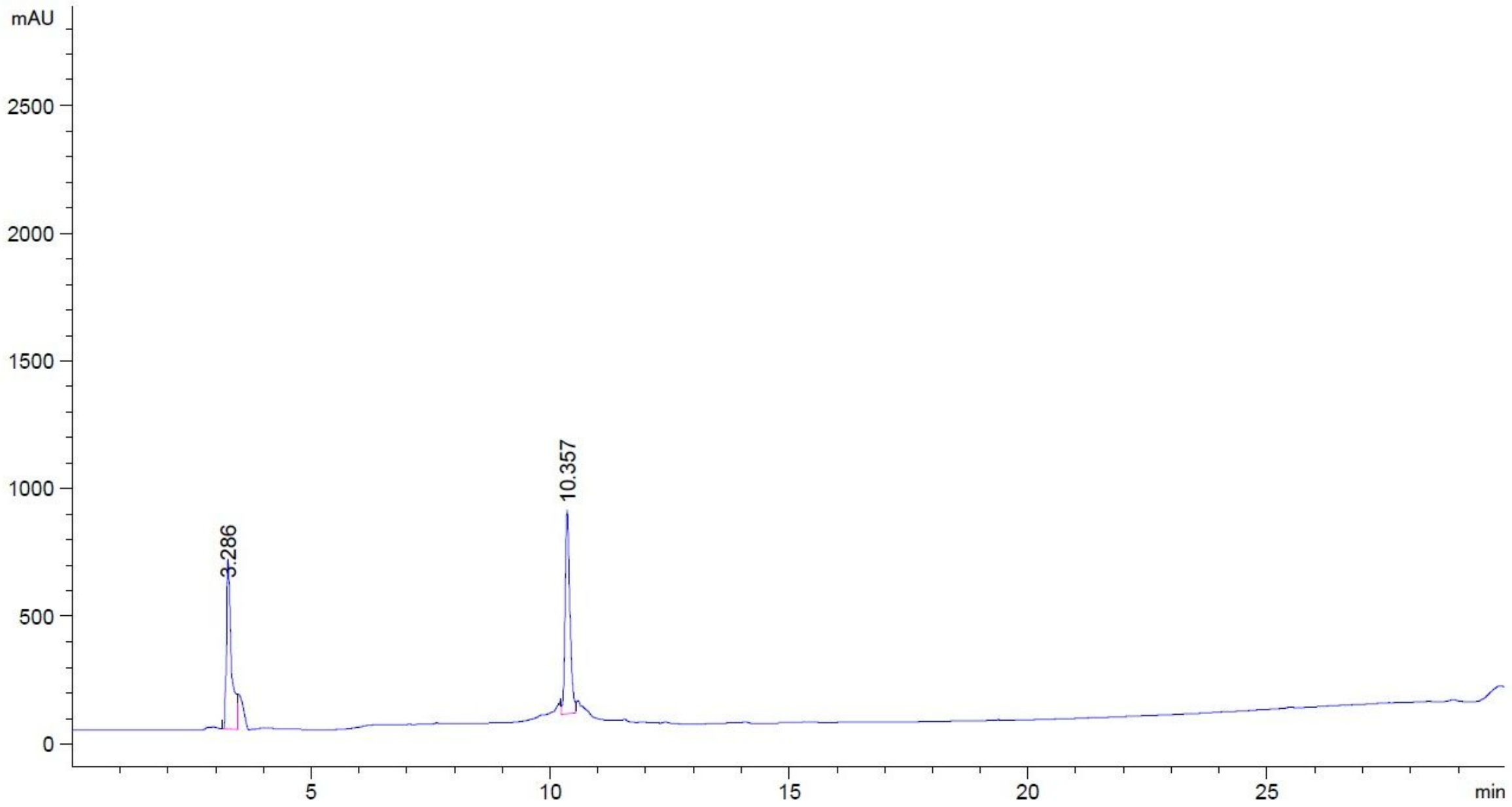
mAU
2500
2000
1500
1000
500
0
3.286
10.357
5
10
15
20
25
min

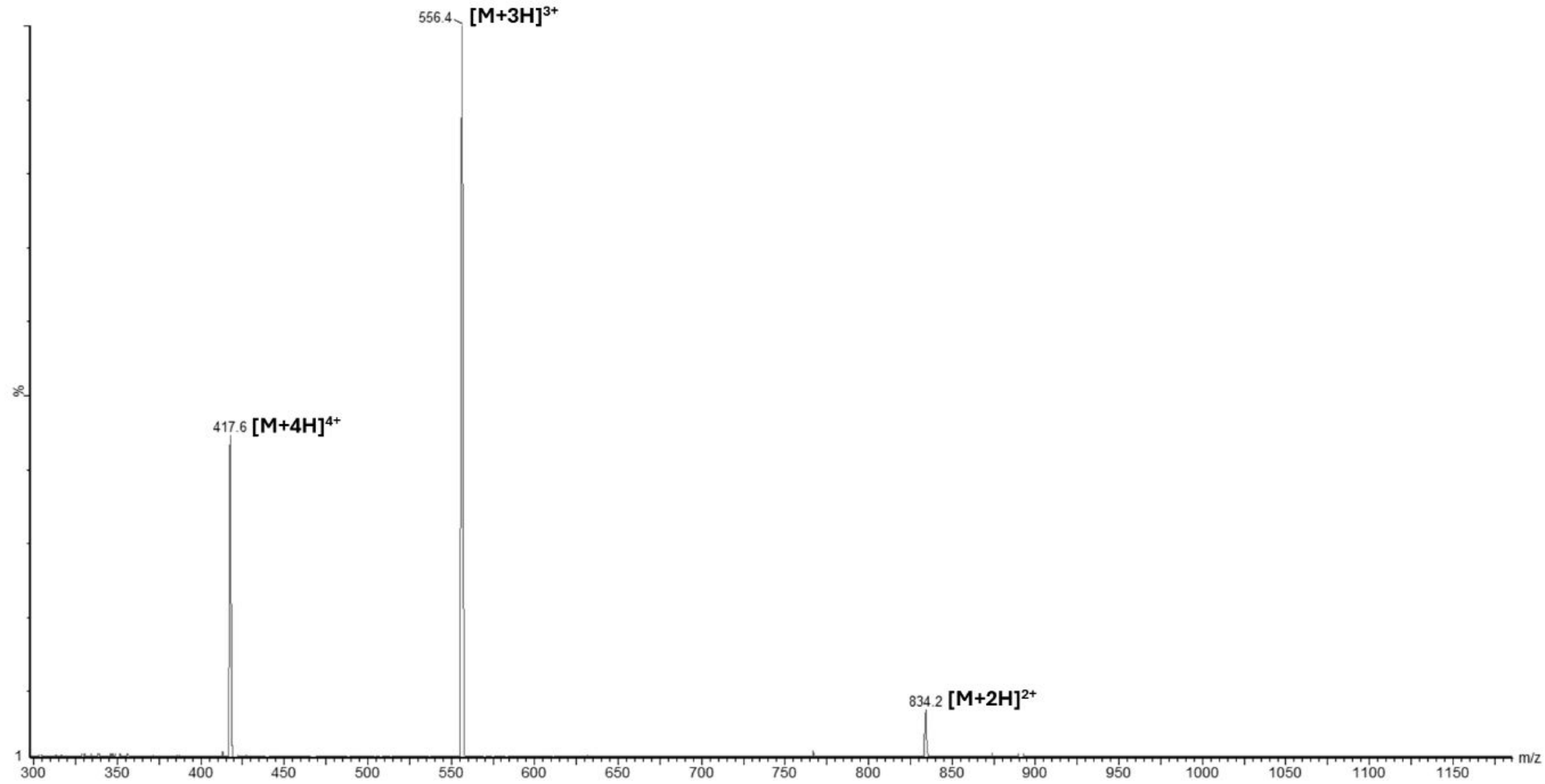
556.4 [M+3H]3+
417.6 [M+4H]4+
834.2 [M+2H]2+
%
1
300
350
400
450
500
550
600
650
700
750
800
850
900
950
1000
1050
1100
1150
m/z

*Compound **1g***

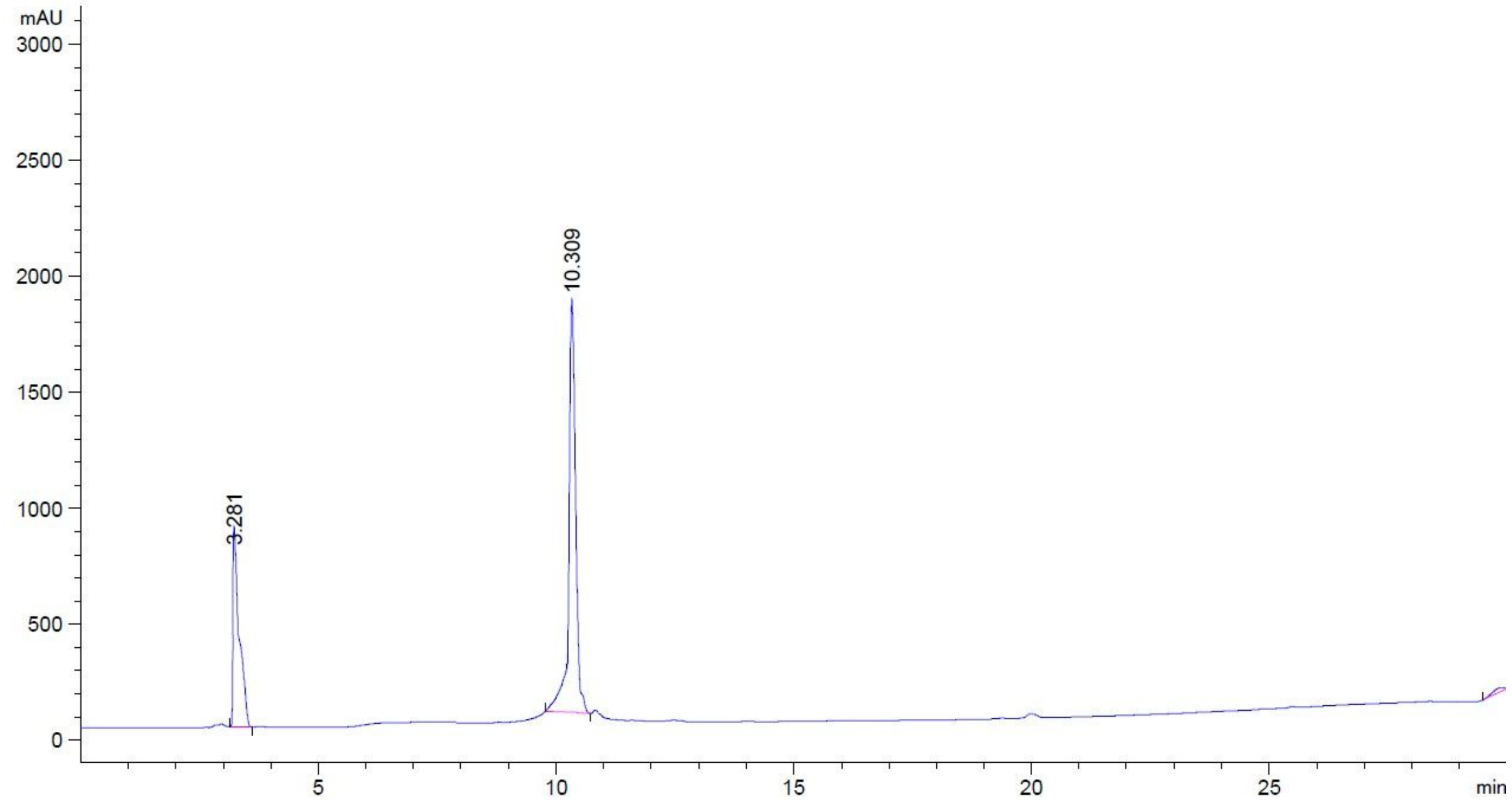
mAU
3000
2500
2000
1500
1000
500
0
3.281
10.309
5
10
15
20
25
min

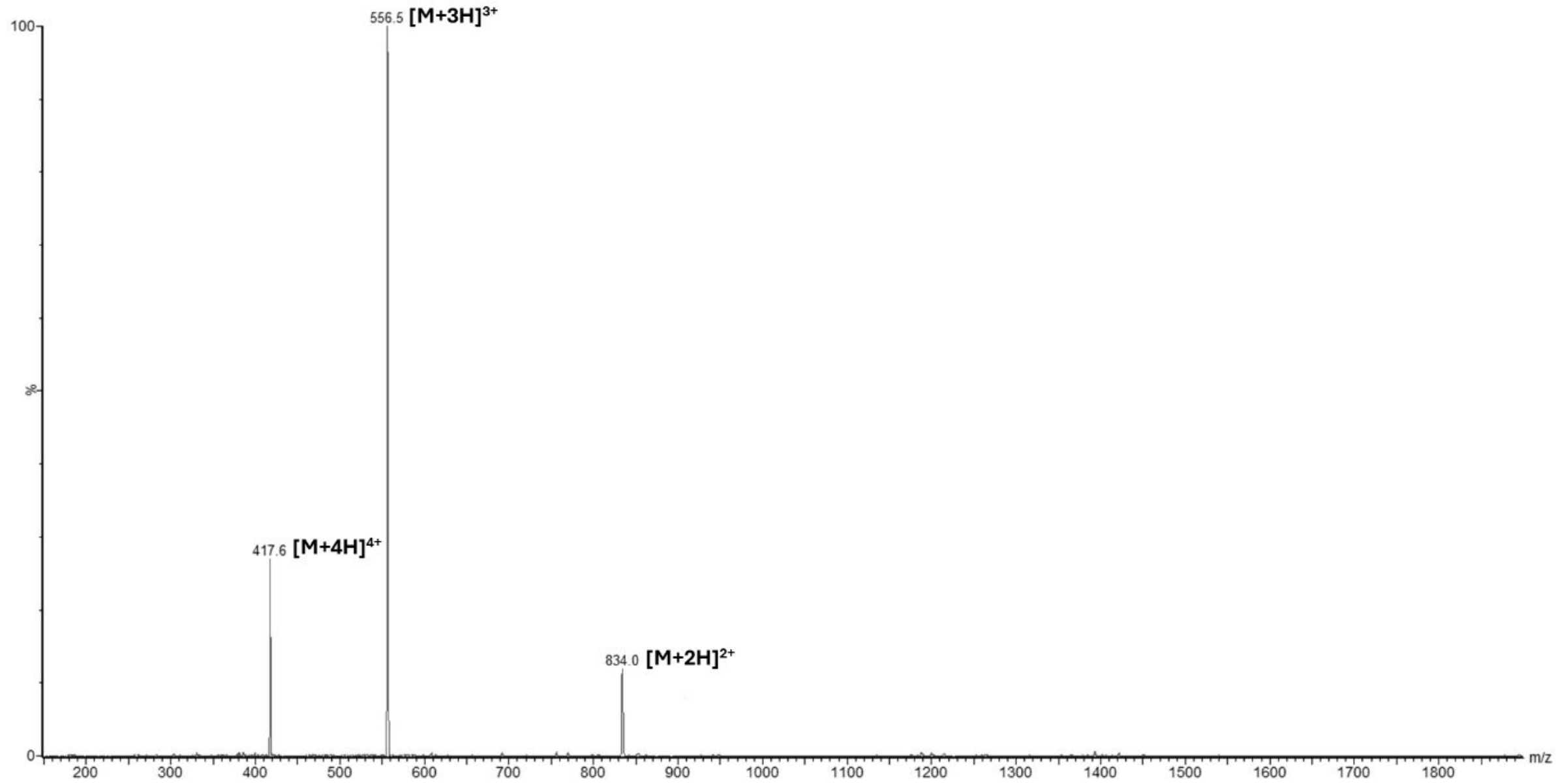
100
%
0
556.5 [M+3H]3+
417.6 [M+4H]4+
834.0 [M+2H]2+
200
300
400
500
600
700
800
900
1000
1100
1200
1300
1400
1500
1600
1700
1800
m/z

*Compound **1h***

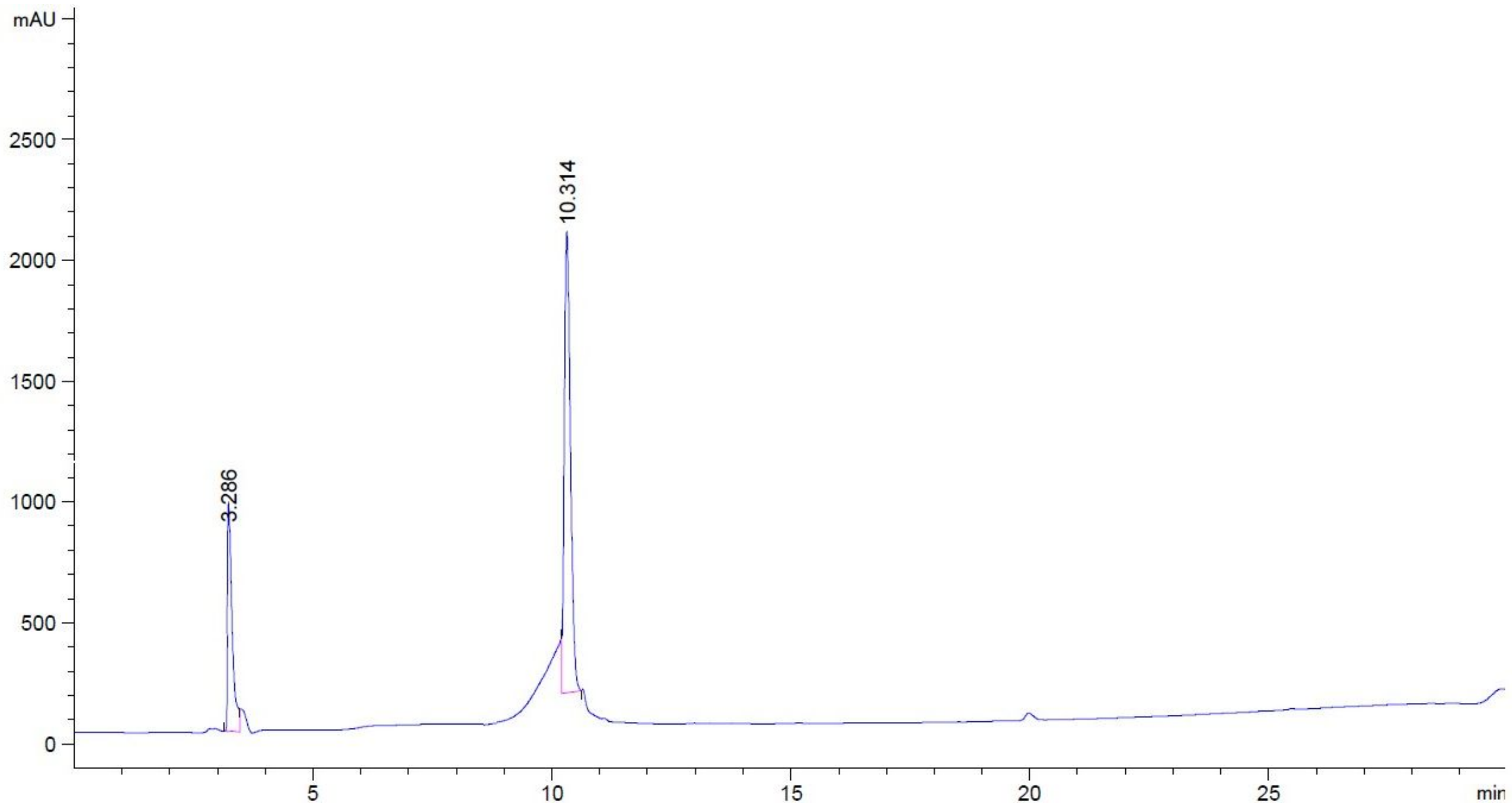
mAU
2500
2000
1500
1000
500
0
10.314
3.286
5
10
15
20
25
min

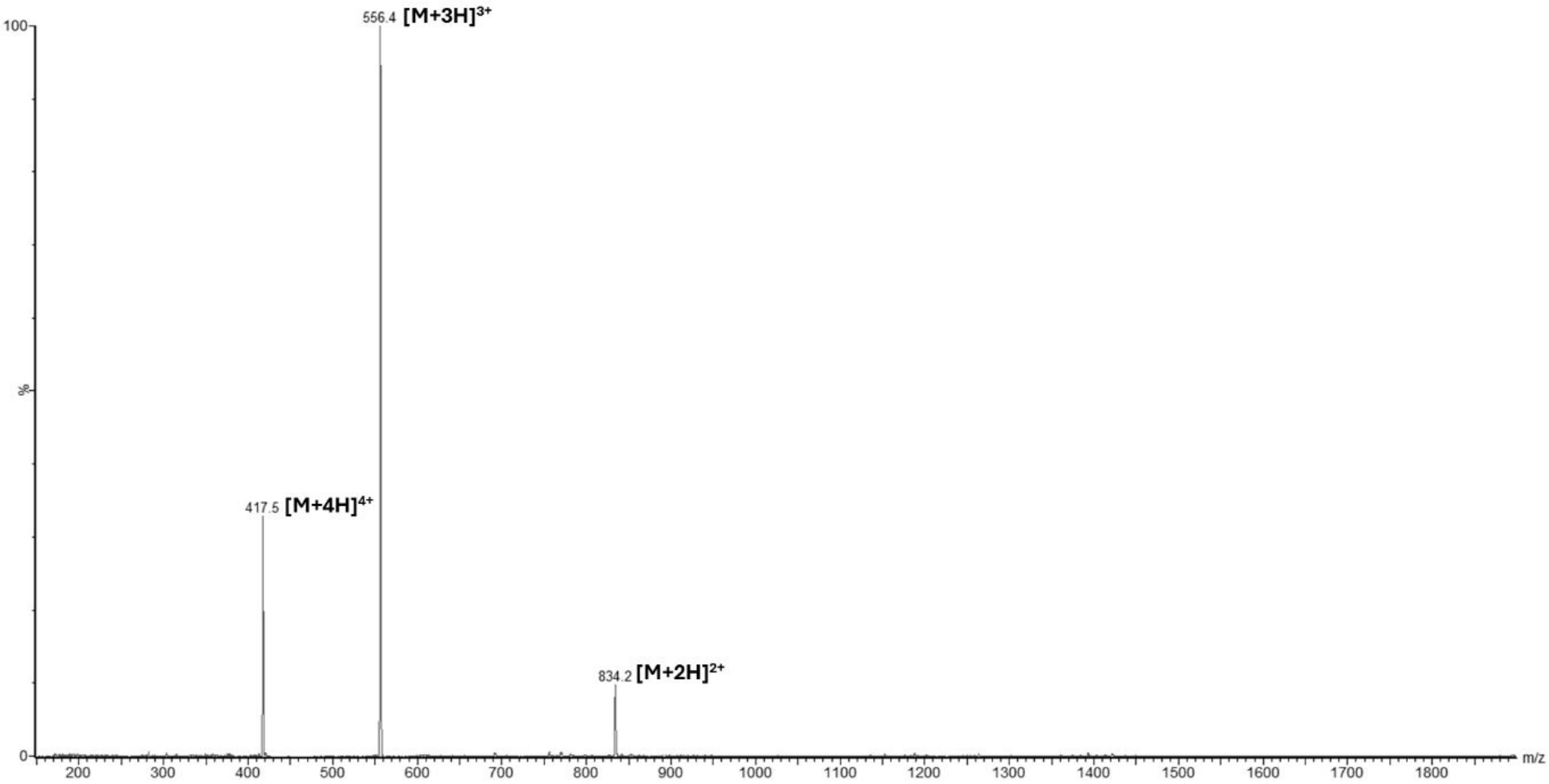
556.4 [M+3H]3+
417.5 [M+4H]4+
834.2 [M+2H]2+
100
%
0
200
300
400
500
600
700
800
900
1000
1100
1200
1300
1400
1500
1600
1700
1800
m/z

*Compound **1i***

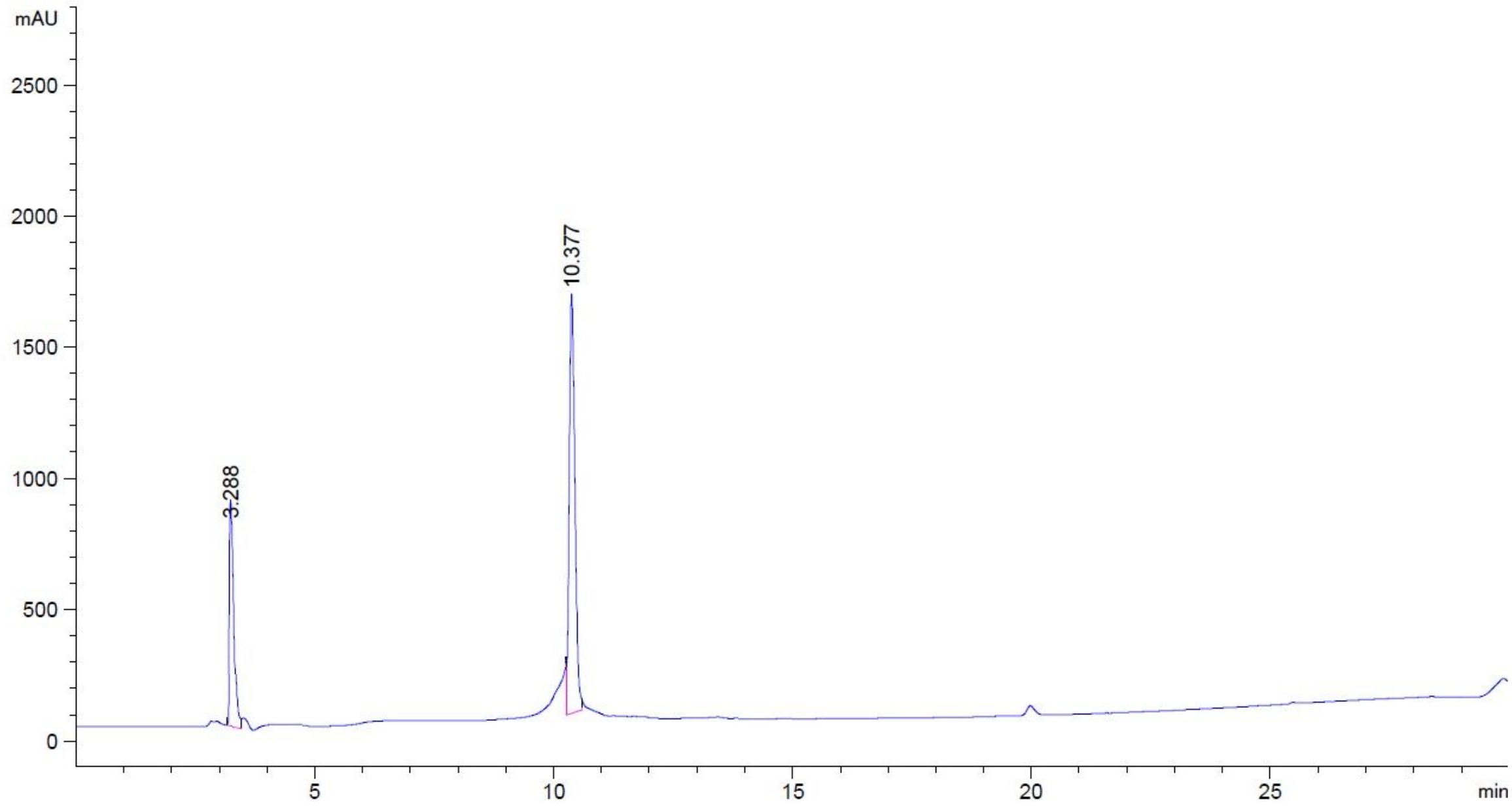

mAU
2500
2000
1500
1000
500
0
10.377
3.288
5
10
15
20
25
min

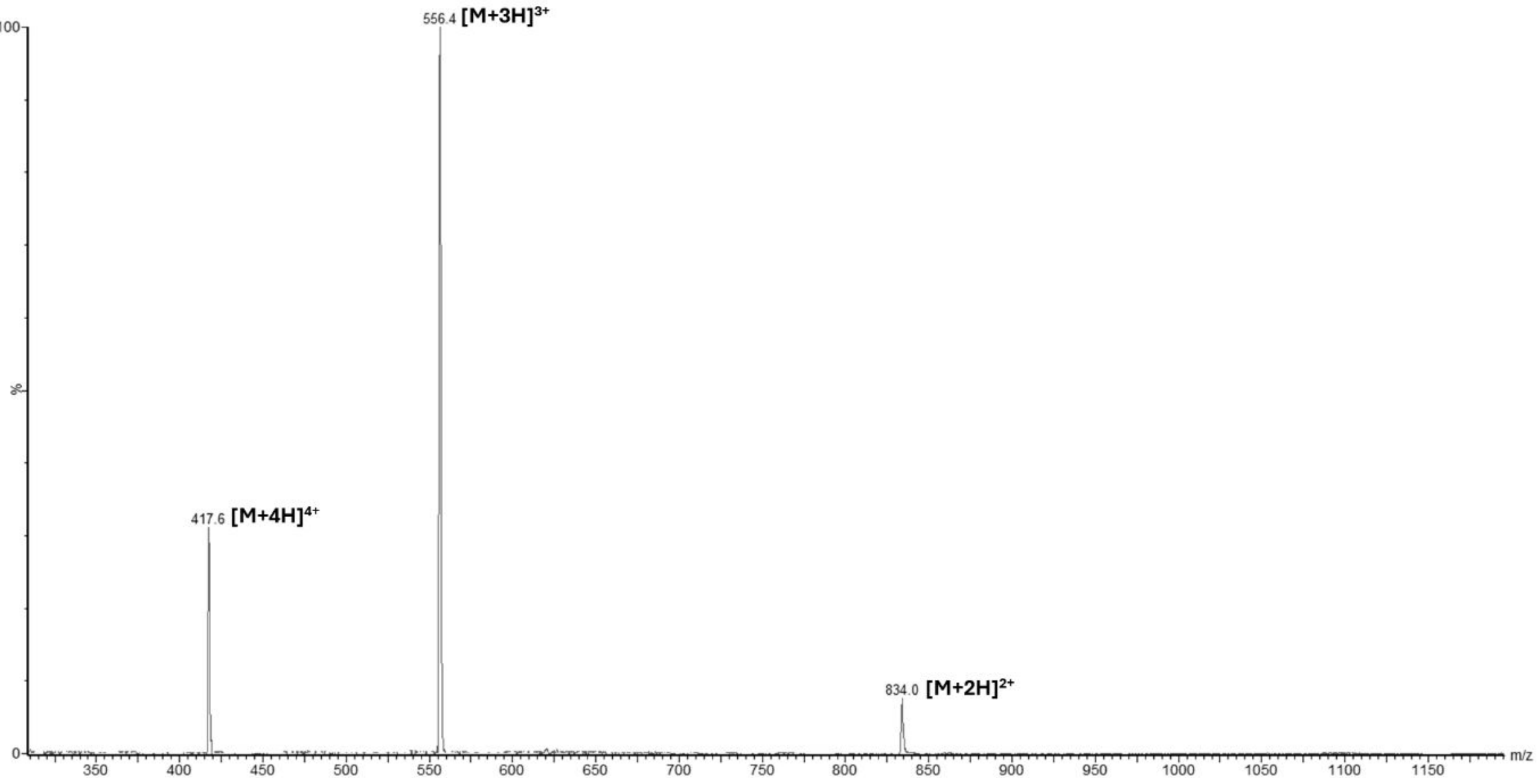

100
%
0
556.4 [M+3H]3+
417.6 [M+4H]4+
834.0 [M+2H]2+
350
400
450
500
550
600
650
700
750
800
850
900
950
1000
1050
1100
1150
m/z

*Compound **2a***

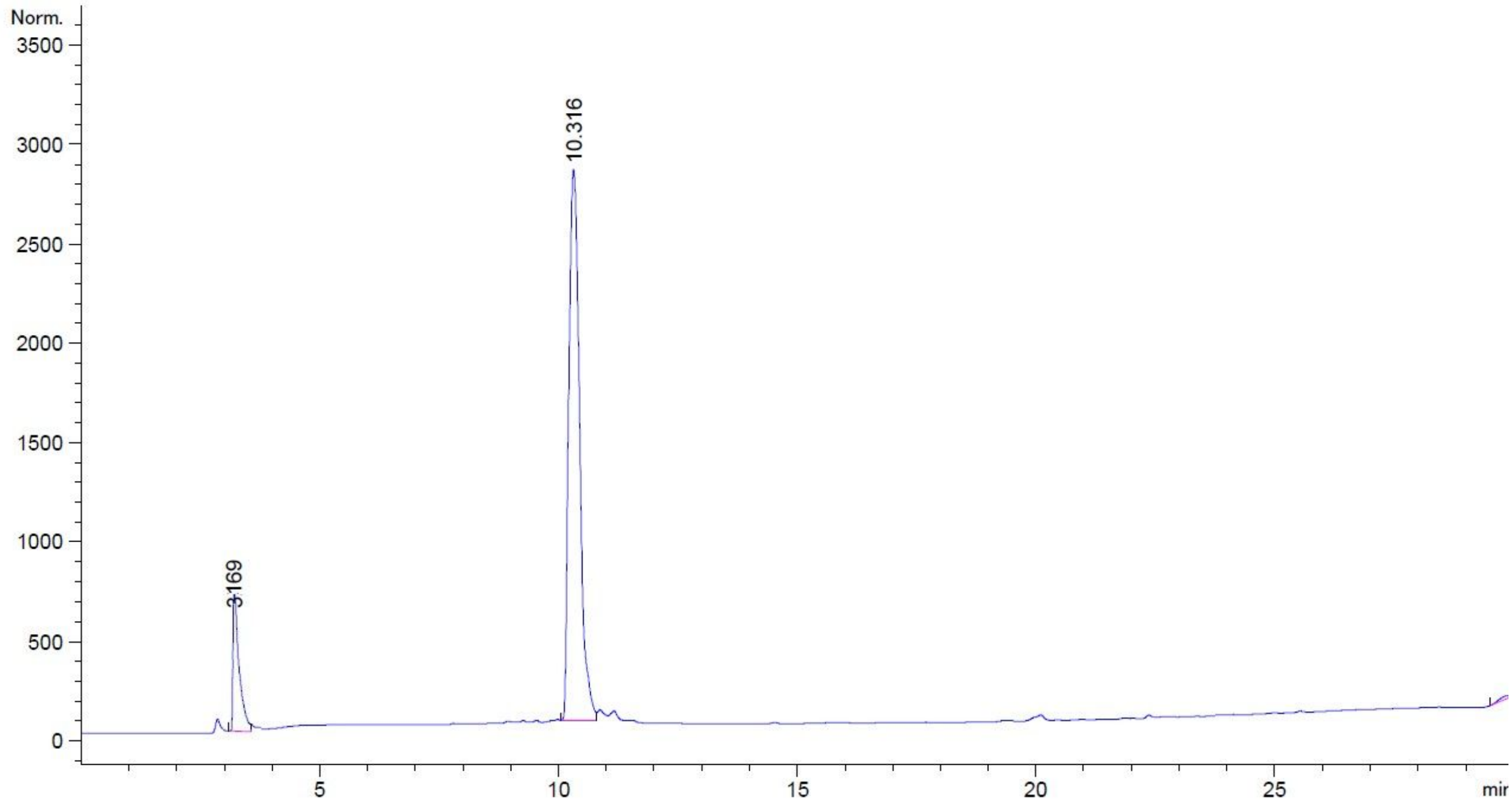

Norm.
3500
3000
2500
2000
1500
1000
500
0
10.316
3.169
5
10
15
20
25
min

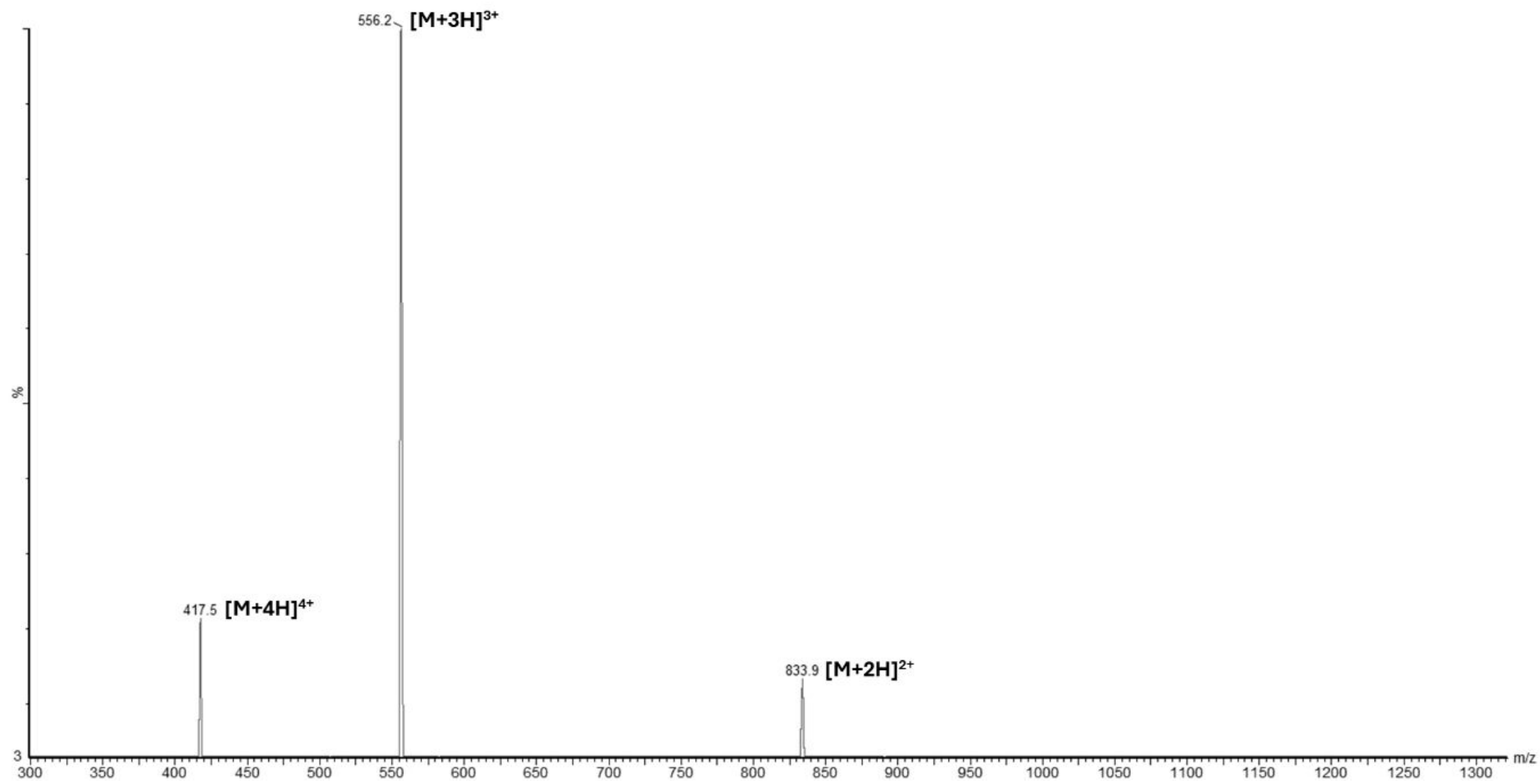

556.2 [M+3H]3+
417.5 [M+4H]4+
833.9 [M+2H]2+
%
m/z

*Compound **2b***

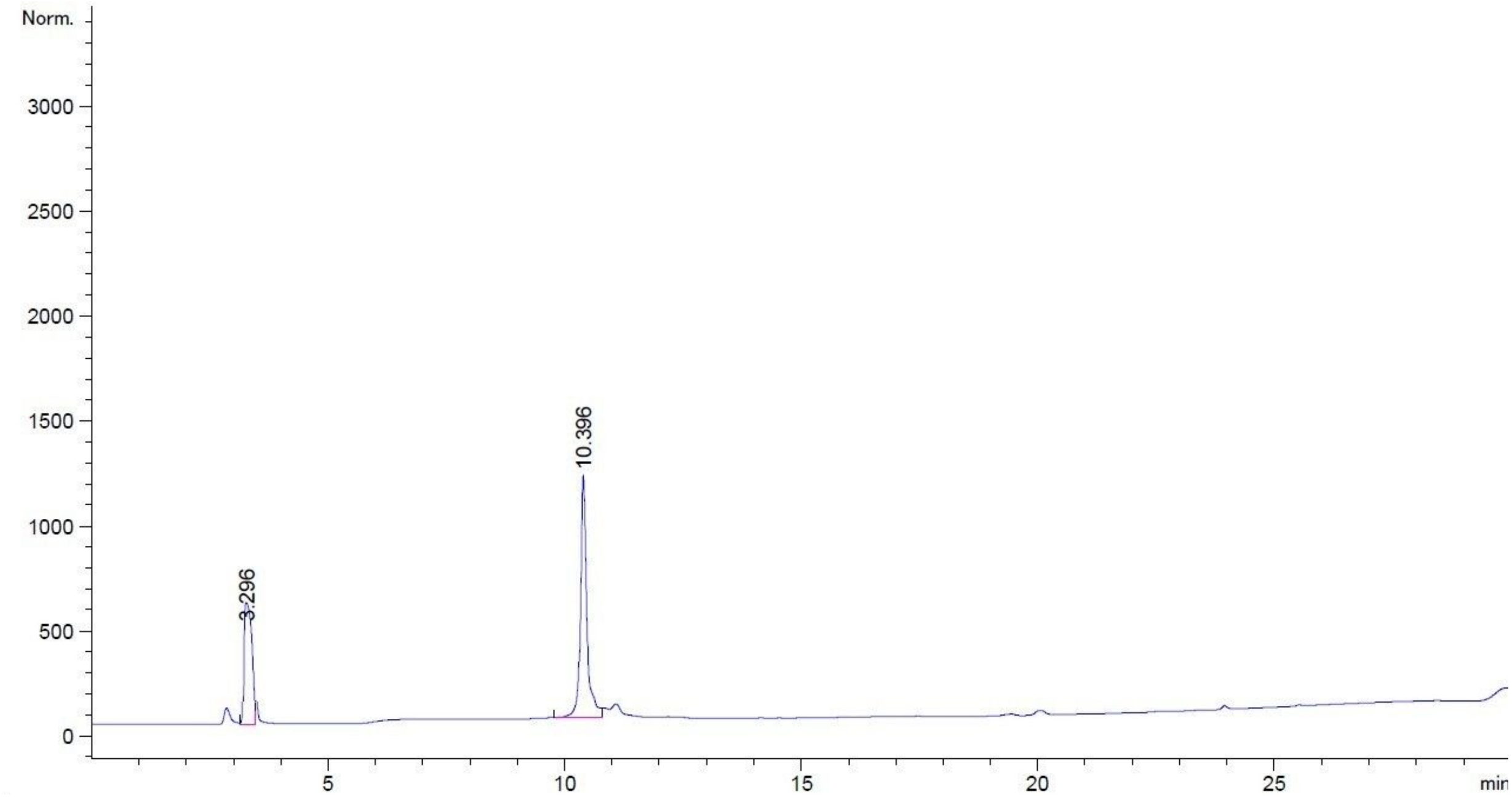

Norm.
3000
2500
2000
1500
1000
500
0
10.396
3.296
5
10
15
20
25
min

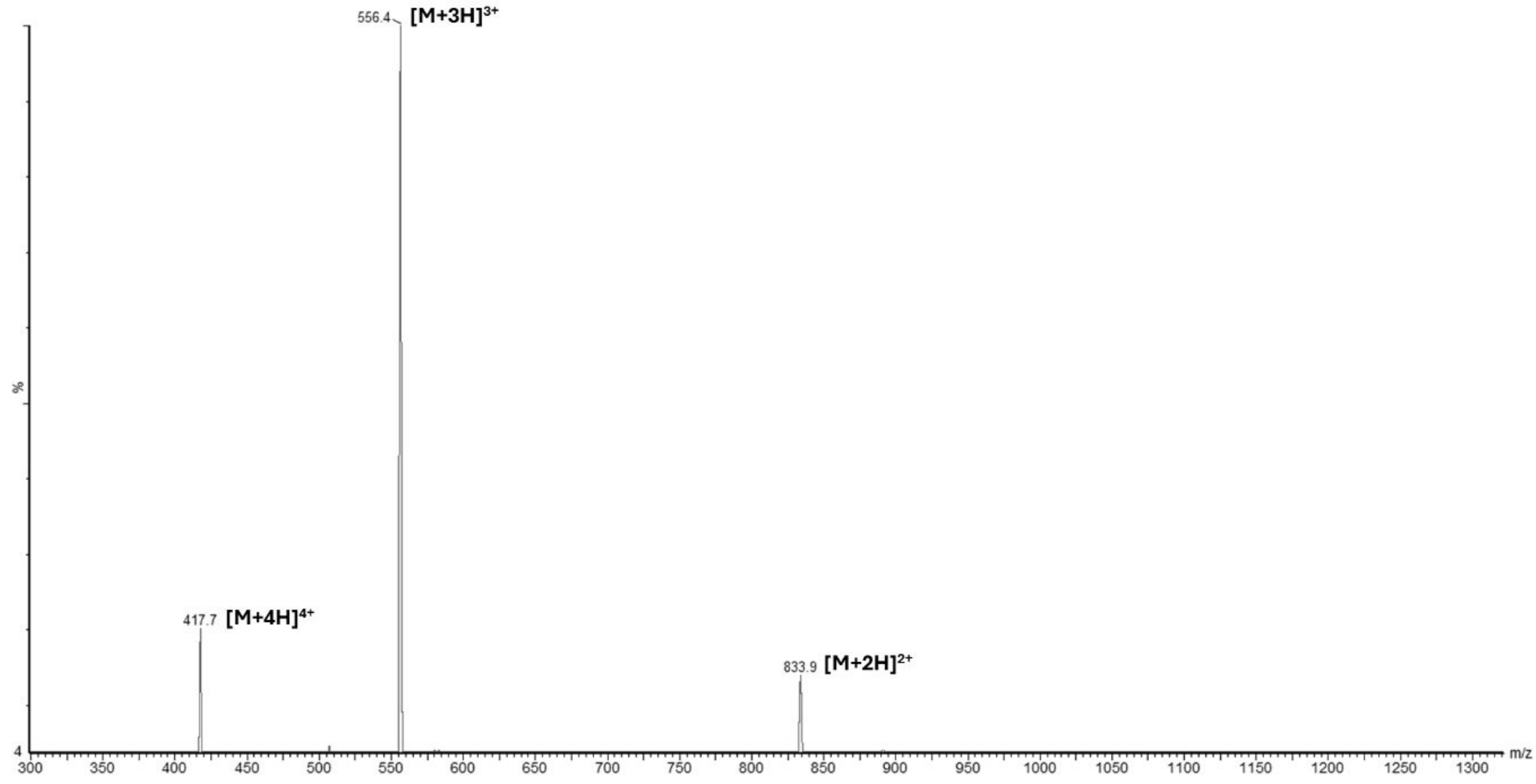

556.4 [M+3H]3+
417.7 [M+4H]4+
833.9 [M+2H]2+
%
m/z

*Compound **2c***

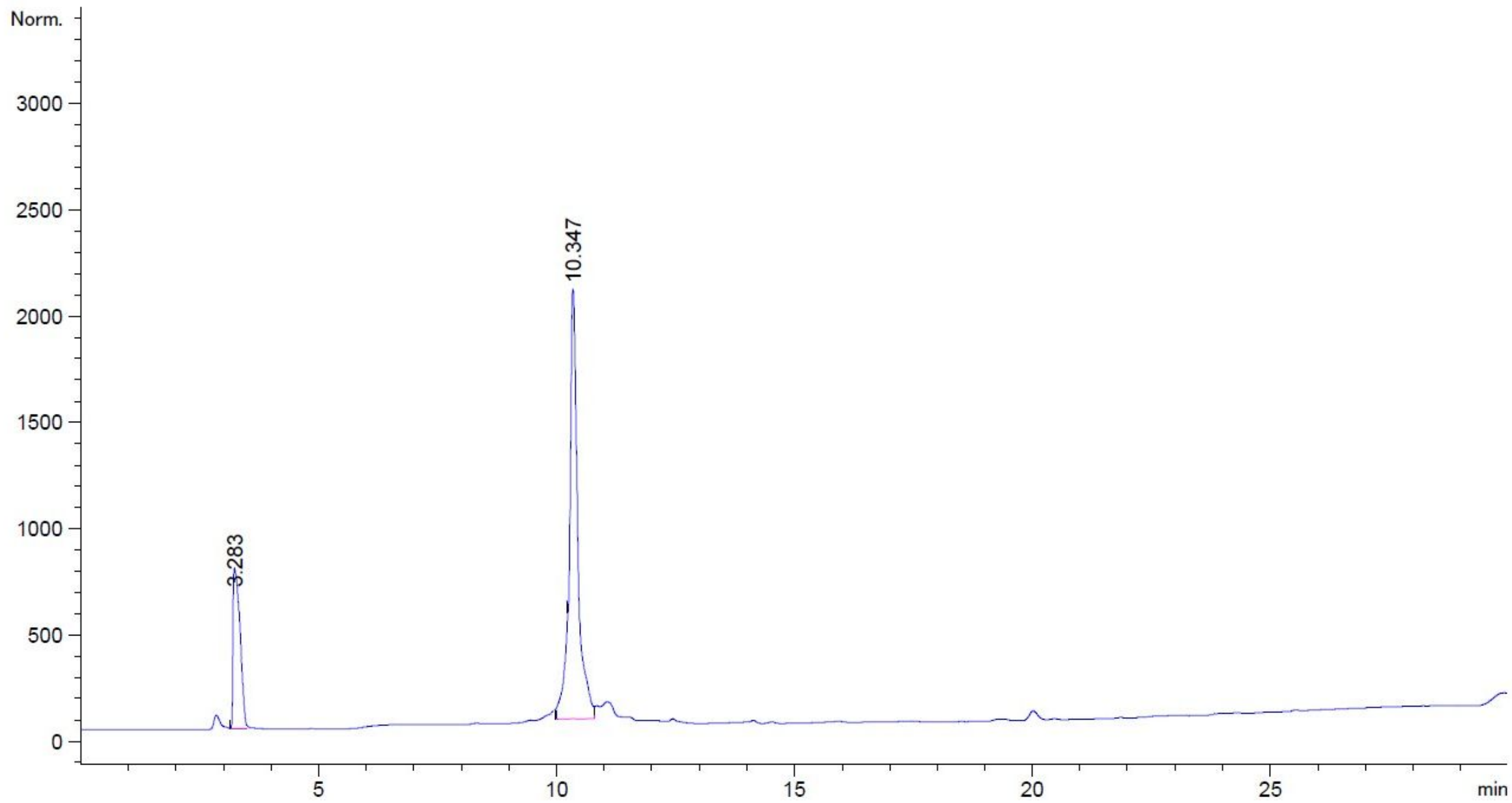

Norm.
3000
2500
2000
1500
1000
500
0
10.347
3.283
5
10
15
20
25
min

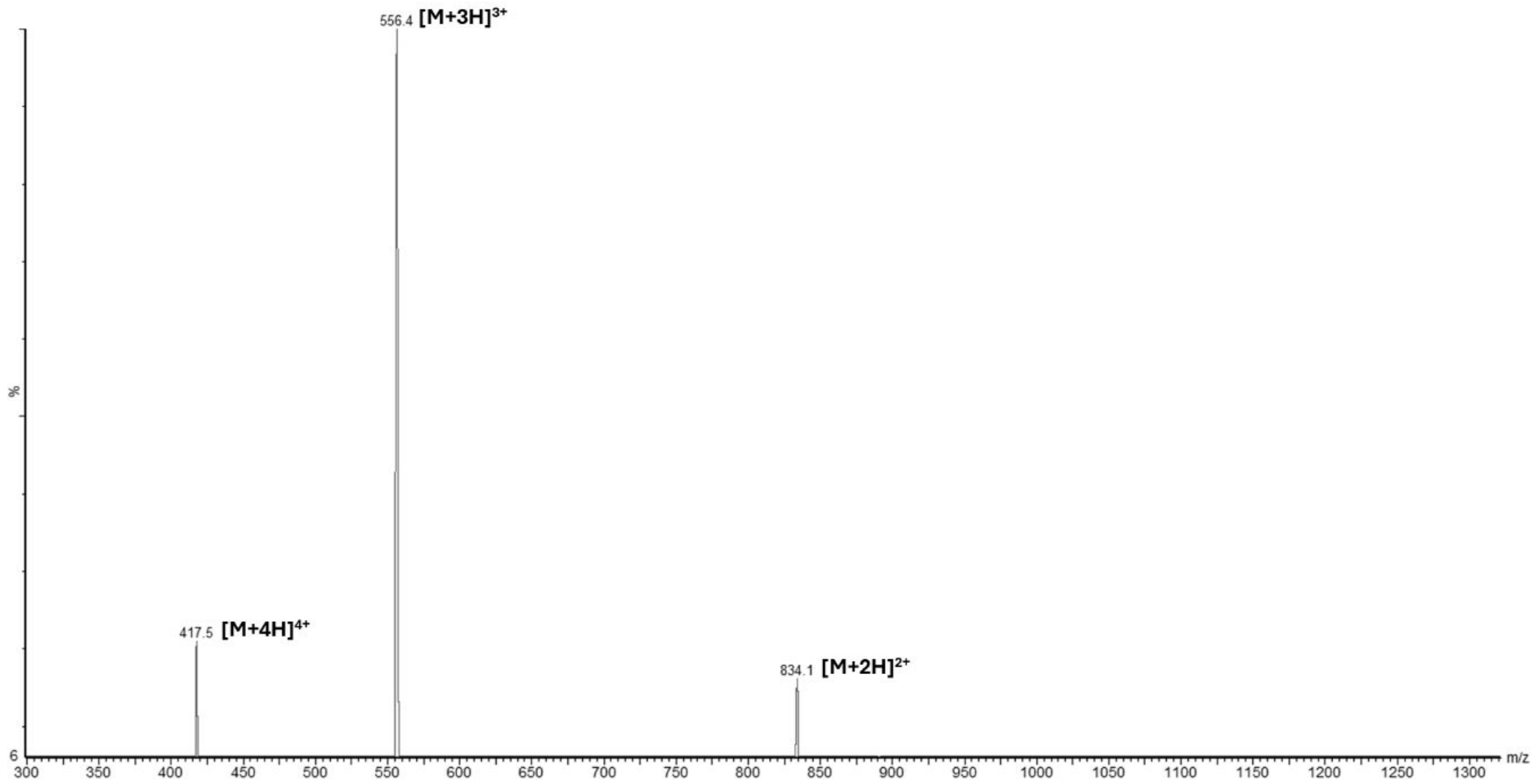

556.4 [M+3H]3+
417.5 [M+4H]4+
834.1 [M+2H]2+
%
6
300
350
400
450
500
550
600
650
700
750
800
850
900
950
1000
1050
1100
1150
1200
1250
1300
m/z

*Compound **2d***

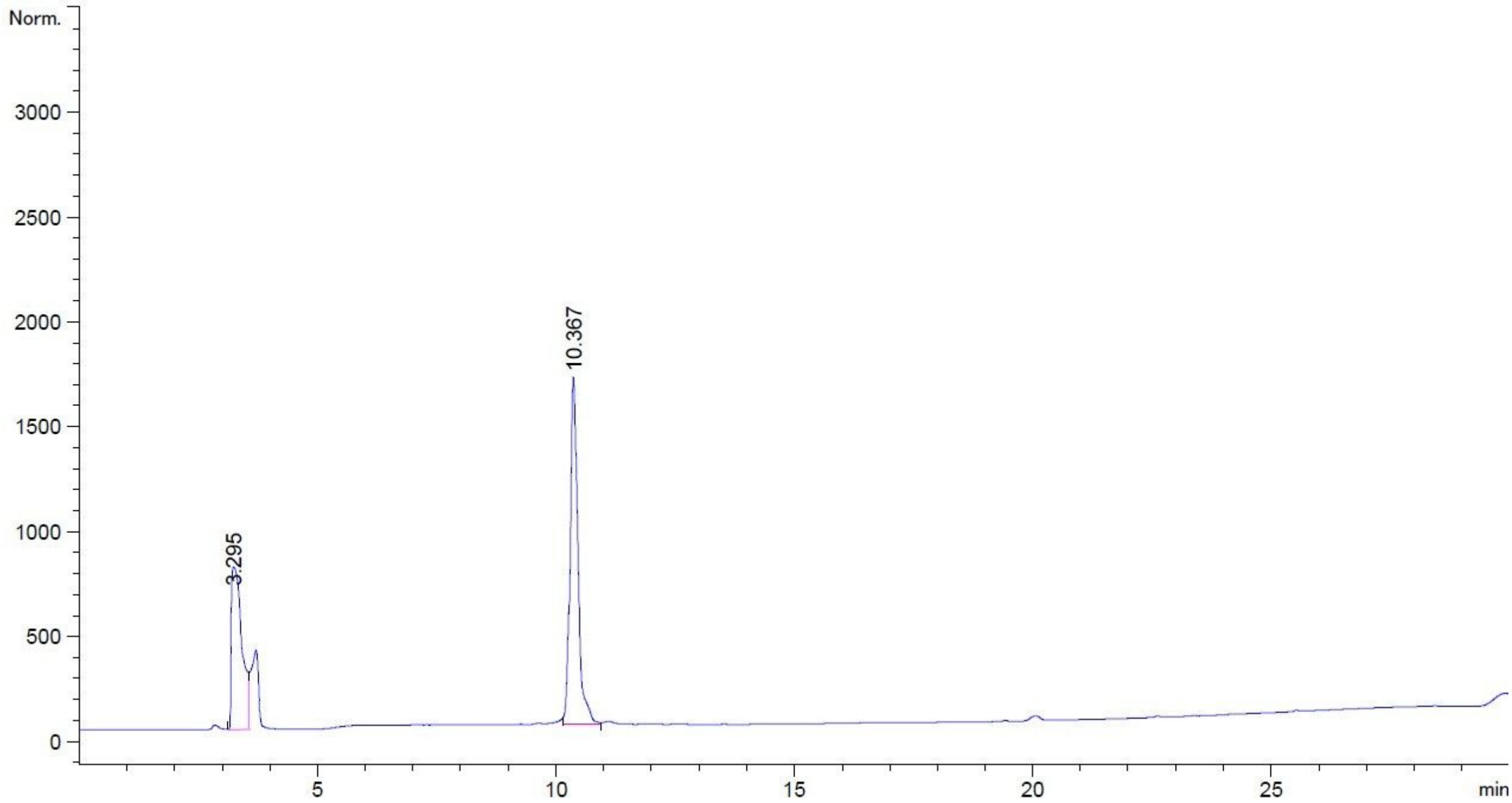

Norm.
3000
2500
2000
1500
1000
500
0
10.367
3.295
5
10
15
20
25
min

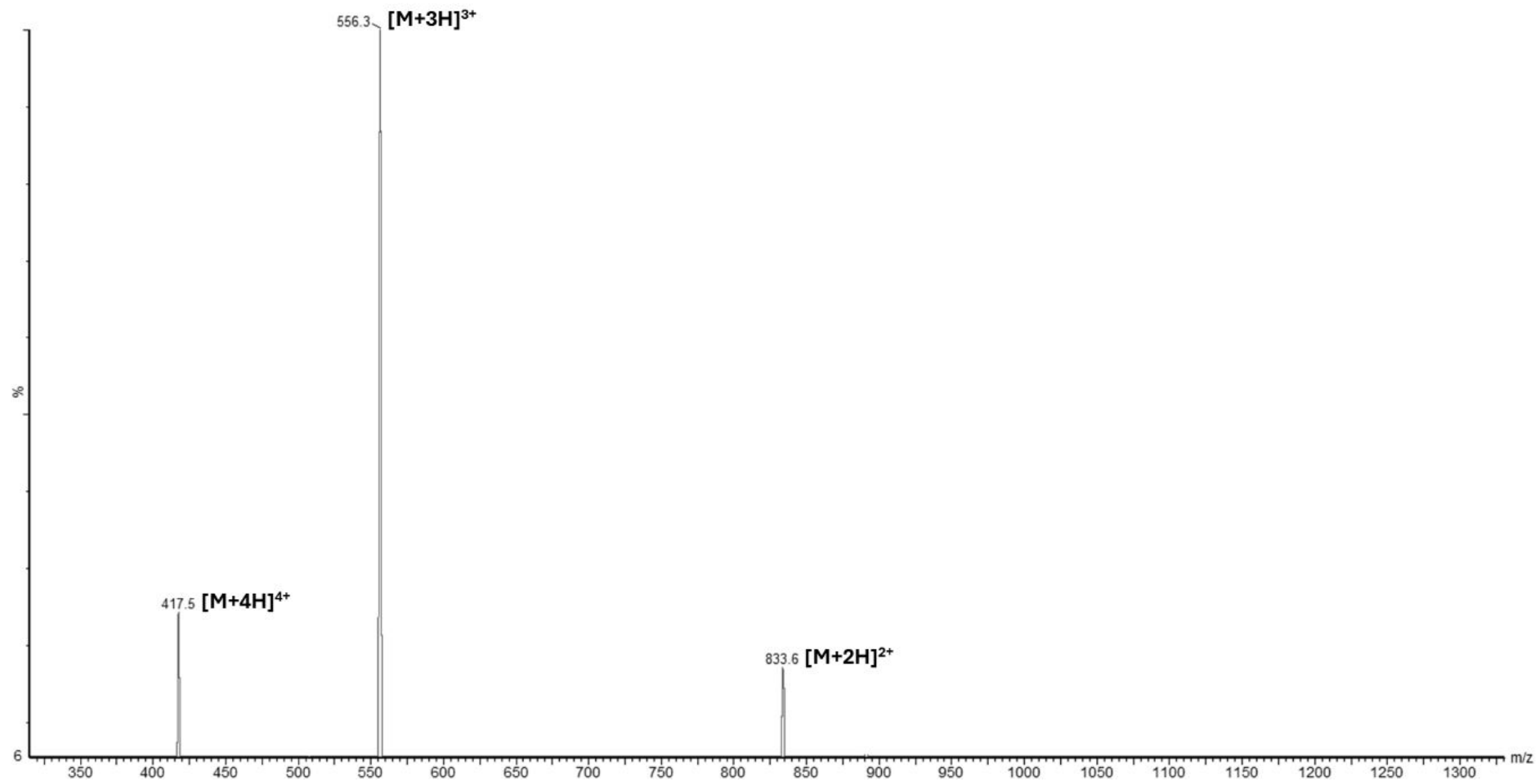

556.3 [M+3H]3+
417.5 [M+4H]4+
833.6 [M+2H]2+
%
m/z

Compound ***2e***

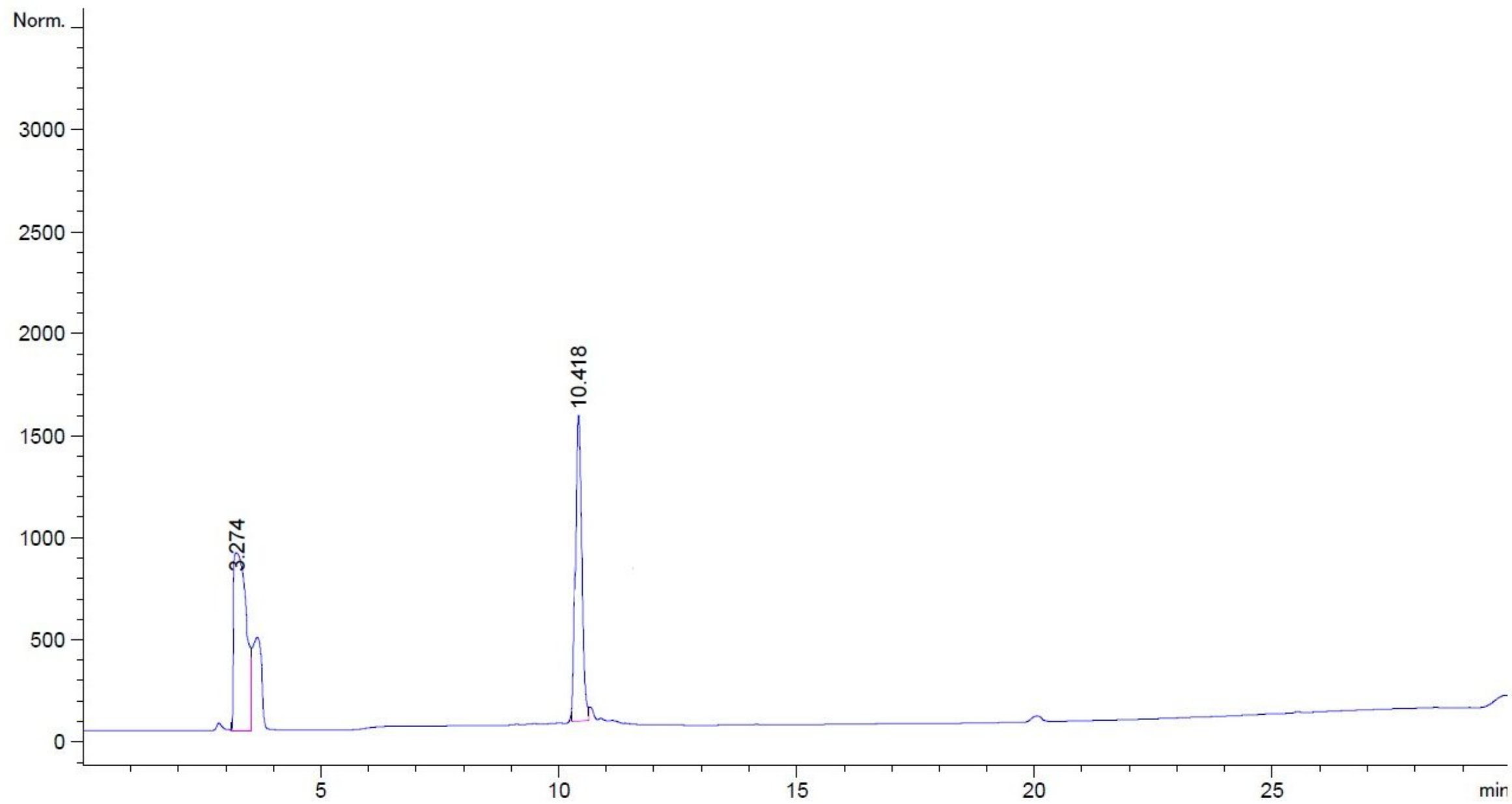

Norm.
3000
2500
2000
1500
1000
500
0
10.418
3.274
5
10
15
20
25
min

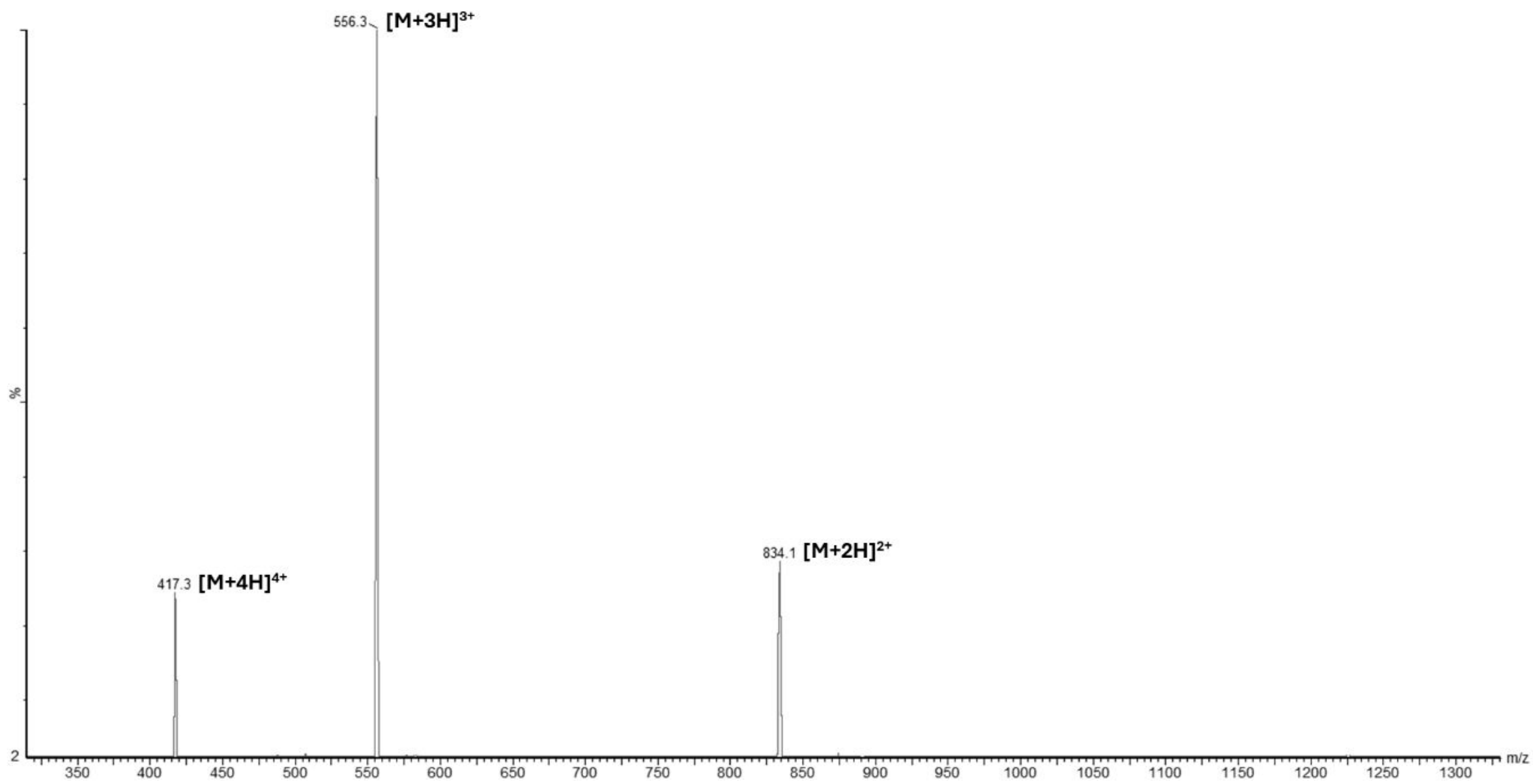

556.3 [M+3H]3+
834.1 [M+2H]2+
417.3 [M+4H]4+
%
2
350
400
450
500
550
600
650
700
750
800
850
900
950
1000
1050
1100
1150
1200
1250
1300
m/z

*Compound **2f***

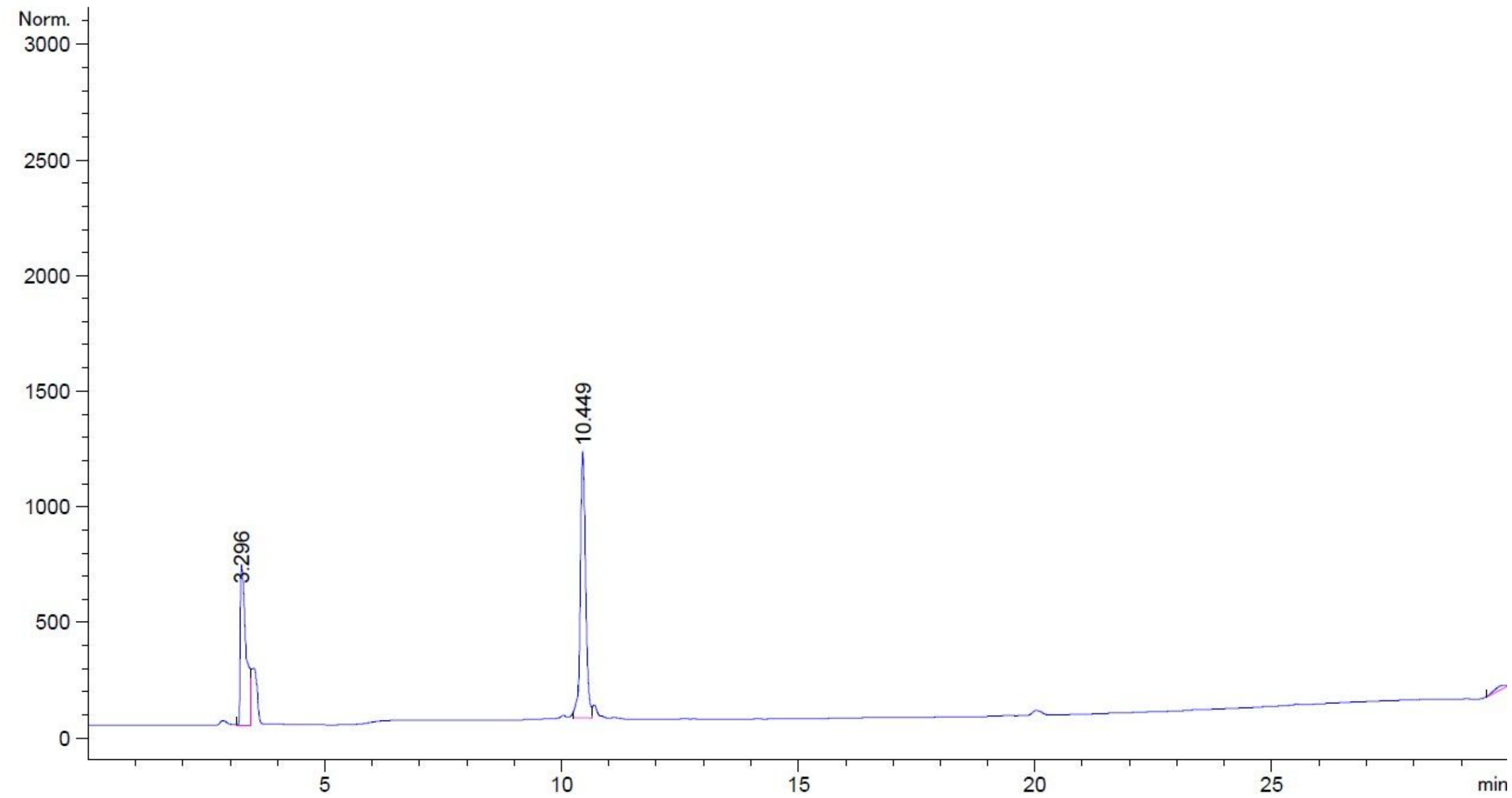

Norm.
3000
2500
2000
1500
1000
500
0
3.296
10.449
5
10
15
20
25
min

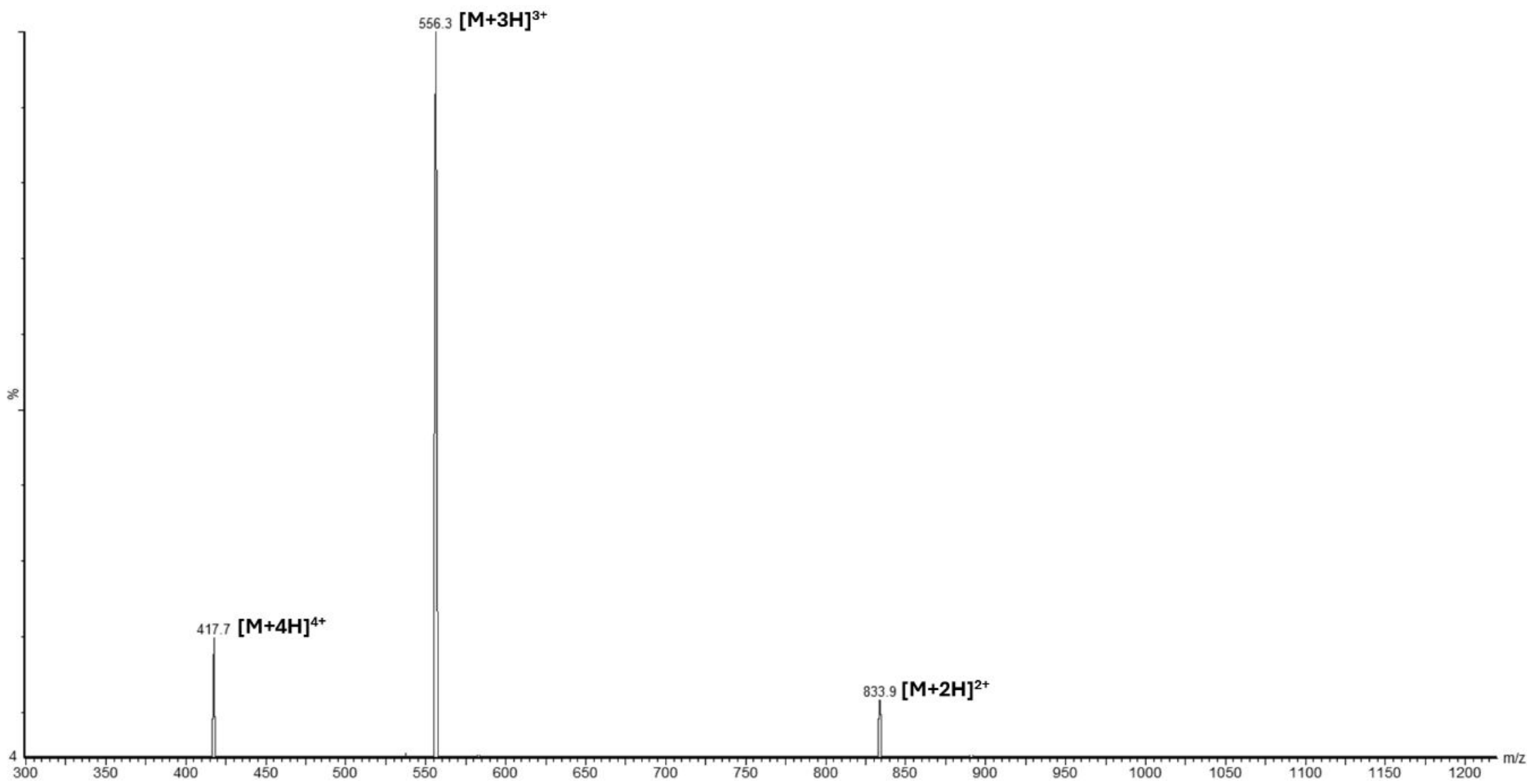

556.3 [M+3H]3+
417.7 [M+4H]4+
833.9 [M+2H]2+
%
4
300
350
400
450
500
550
600
650
700
750
800
850
900
950
1000
1050
1100
1150
1200
m/z

*Compound **2g***

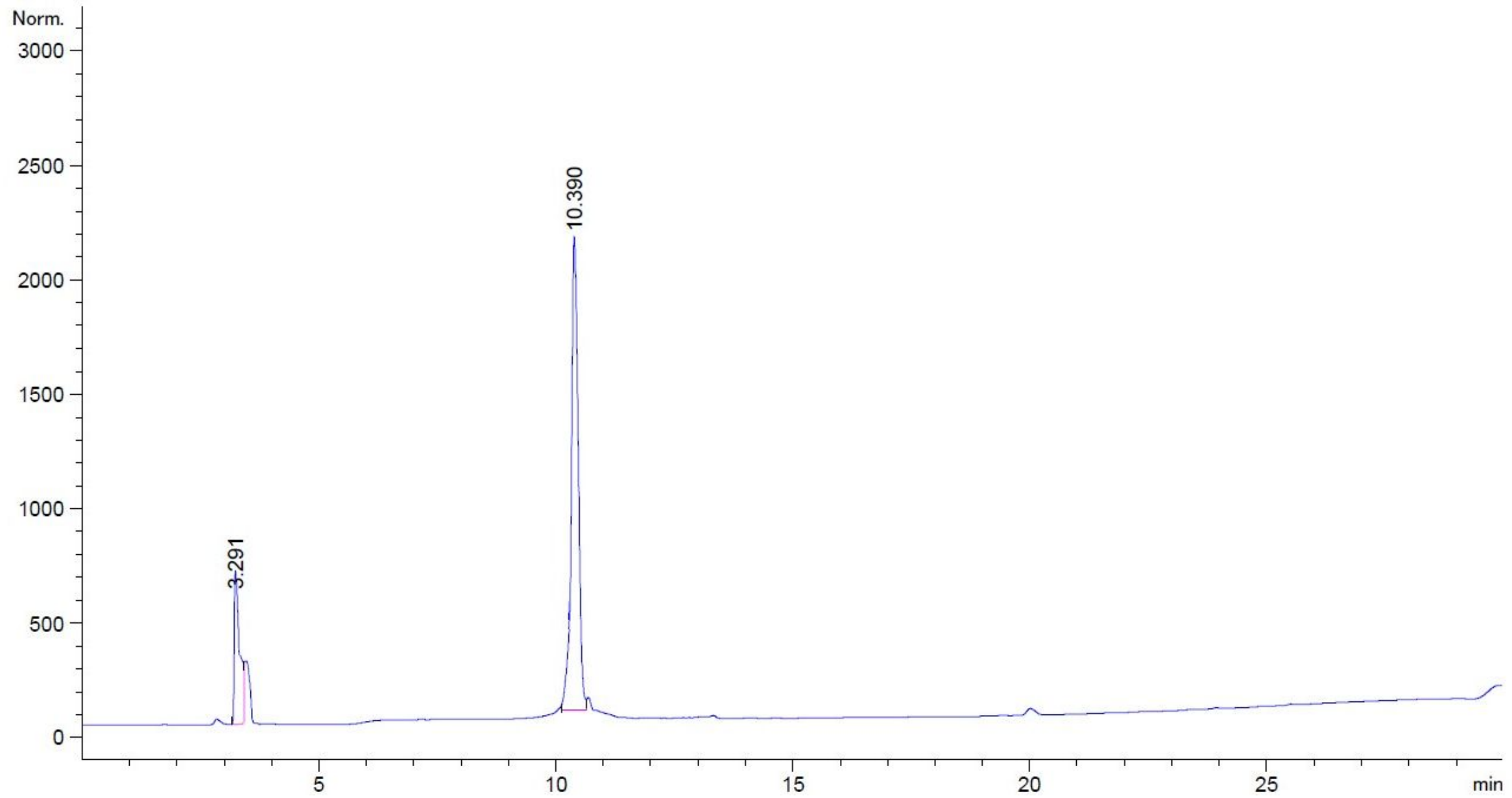

Norm.
3000
2500
2000
1500
1000
500
0
10.390
3.291
5
10
15
20
25
min

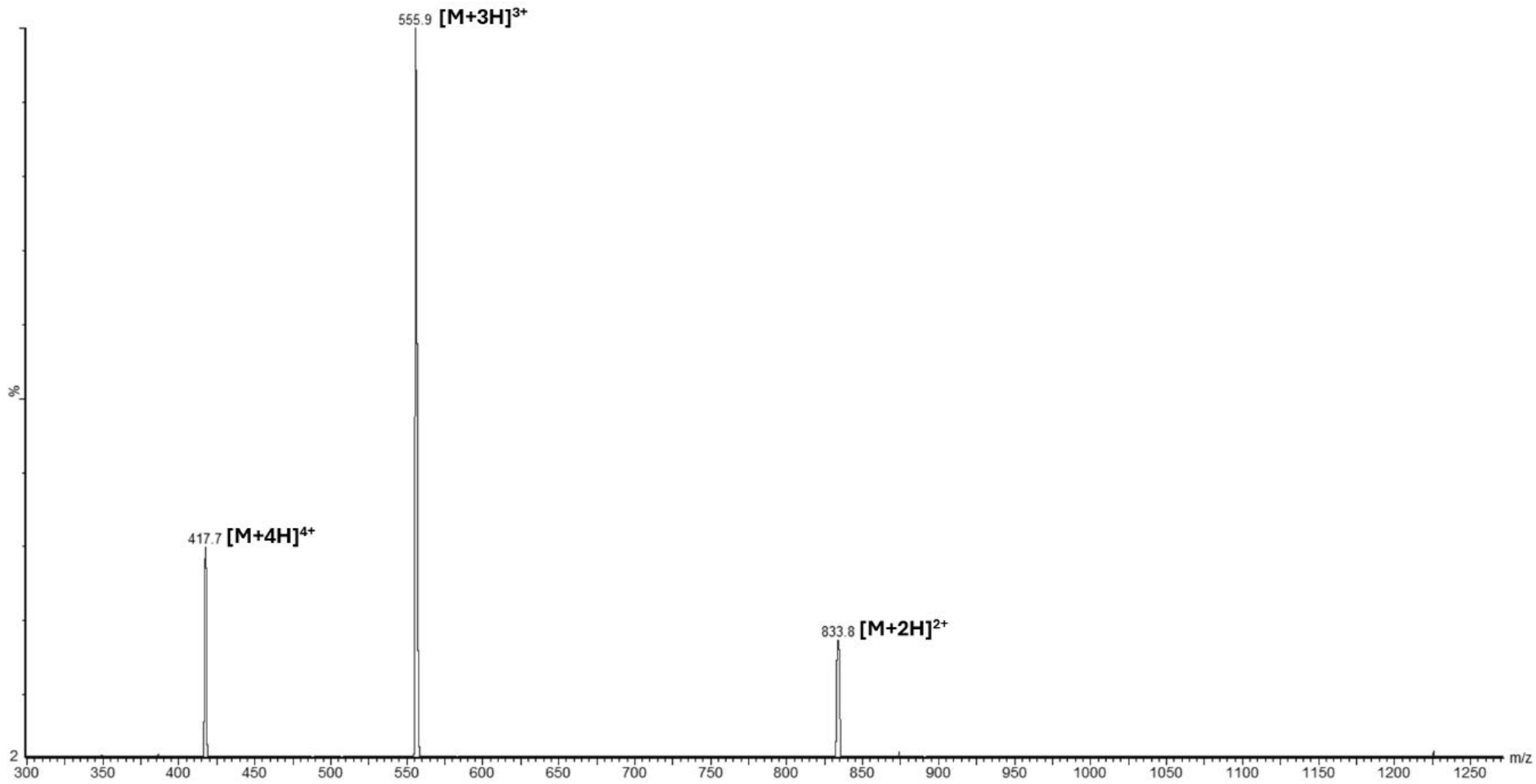

555.9 [M+3H]3+
417.7 [M+4H]4+
833.8 [M+2H]2+
%
m/z

*Compound **2h***

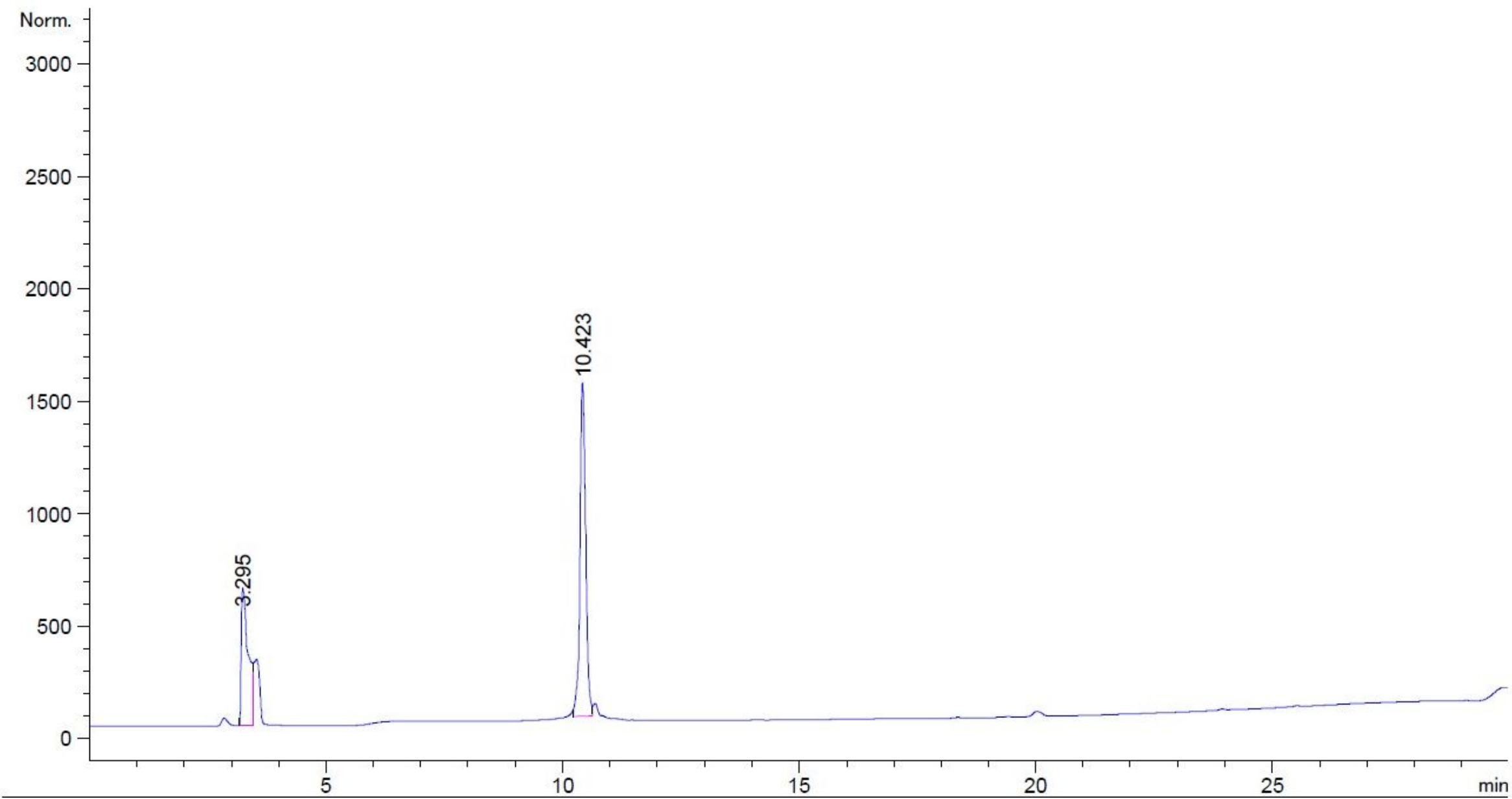

Norm.
3000
2500
2000
1500
1000
500
0
10.423
3.295
5
10
15
20
25
min

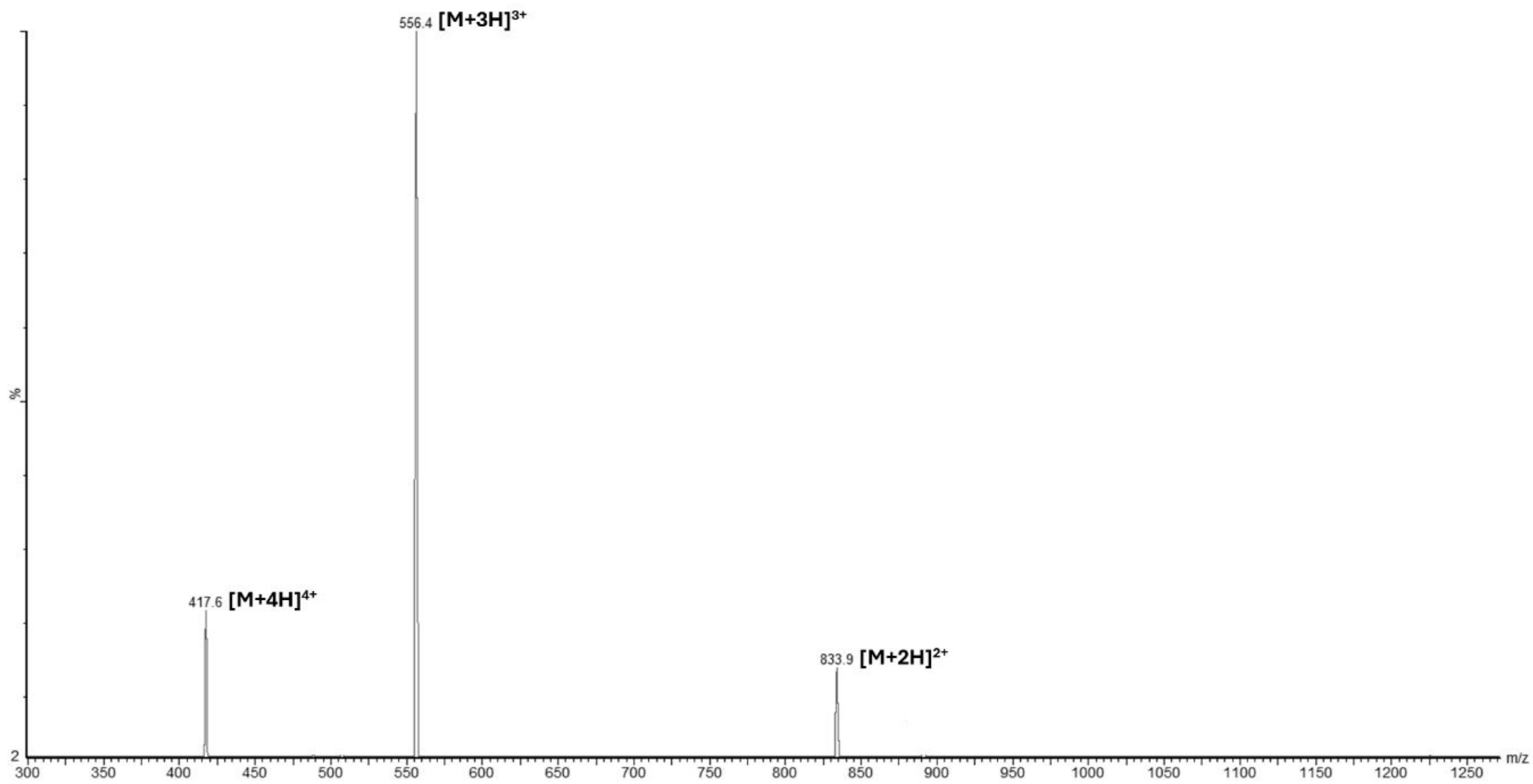

556.4 [M+3H]3+
417.6 [M+4H]4+
833.9 [M+2H]2+
%
m/z

*Compound **3a***

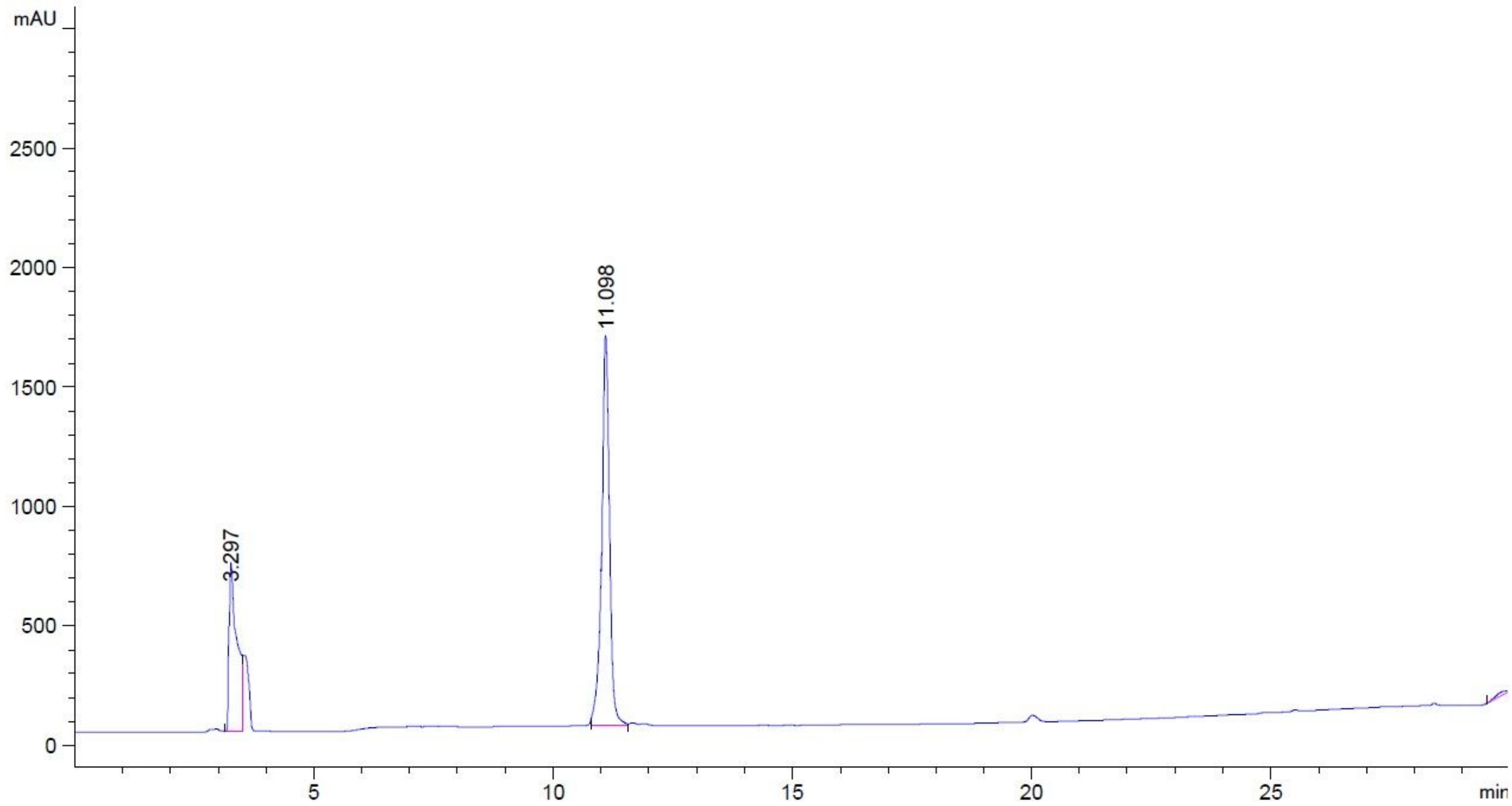
mAU
2500
2000
1500
1000
500
0
11.098
3.297
5
10
15
20
25
min

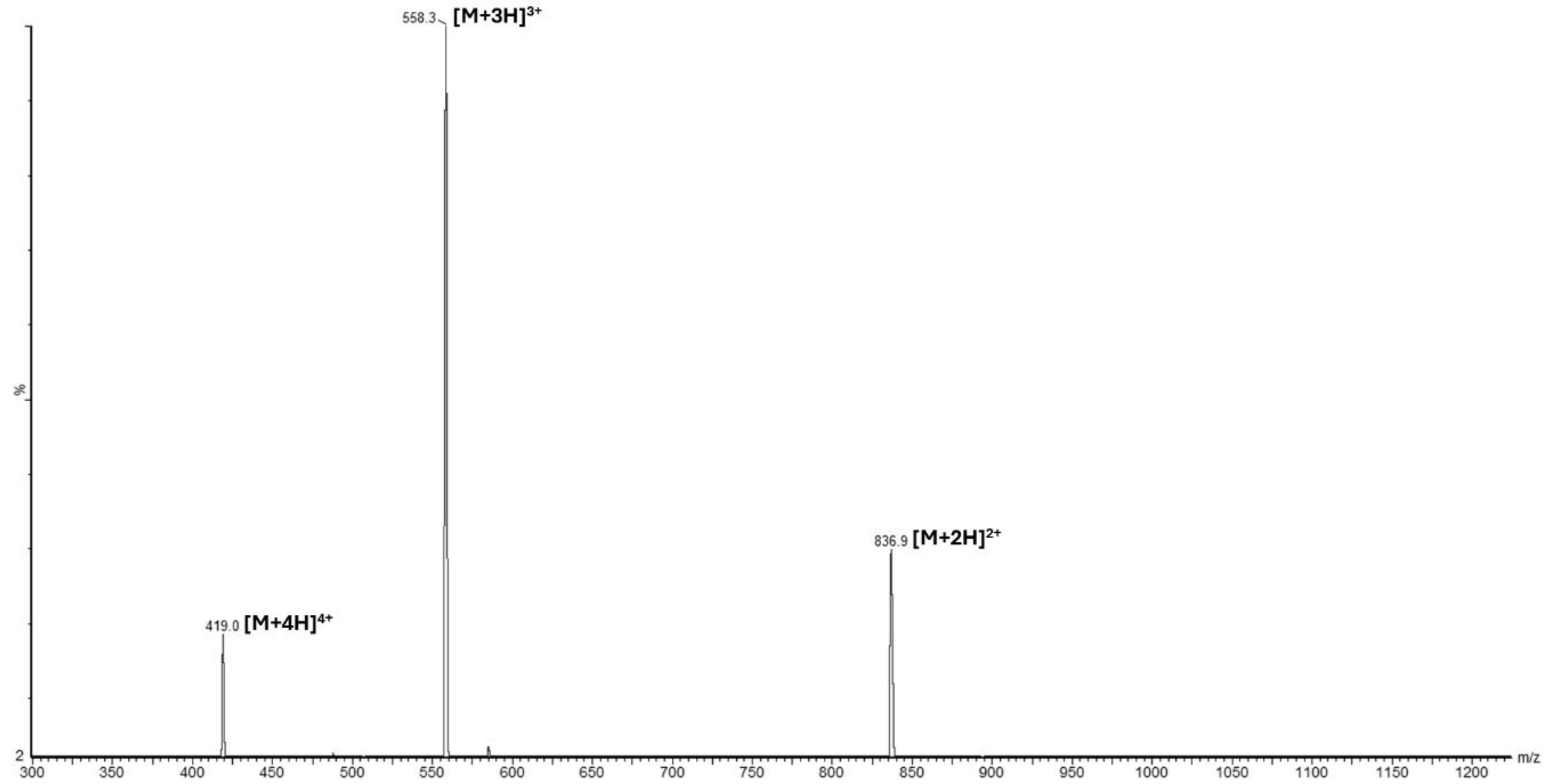
558.3 [M+3H]3+
836.9 [M+2H]2+
419.0 [M+4H]4+
%
2
300
350
400
450
500
550
600
650
700
750
800
850
900
950
1000
1050
1100
1150
1200
m/z

*Compound **3b***

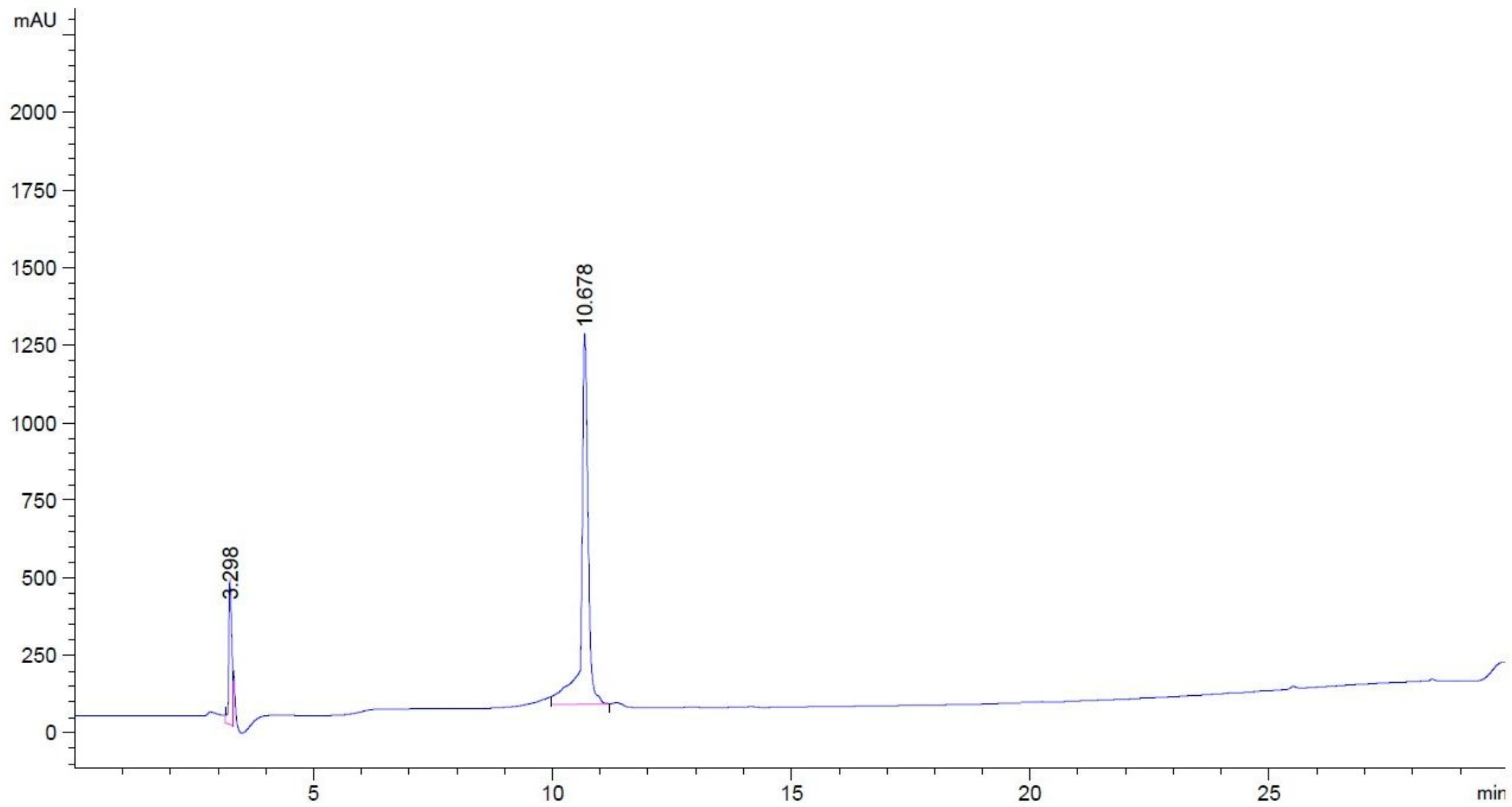
mAU
2000
1750
1500
1250
1000
750
500
250
0
10.678
3.298
5
10
15
20
25
min

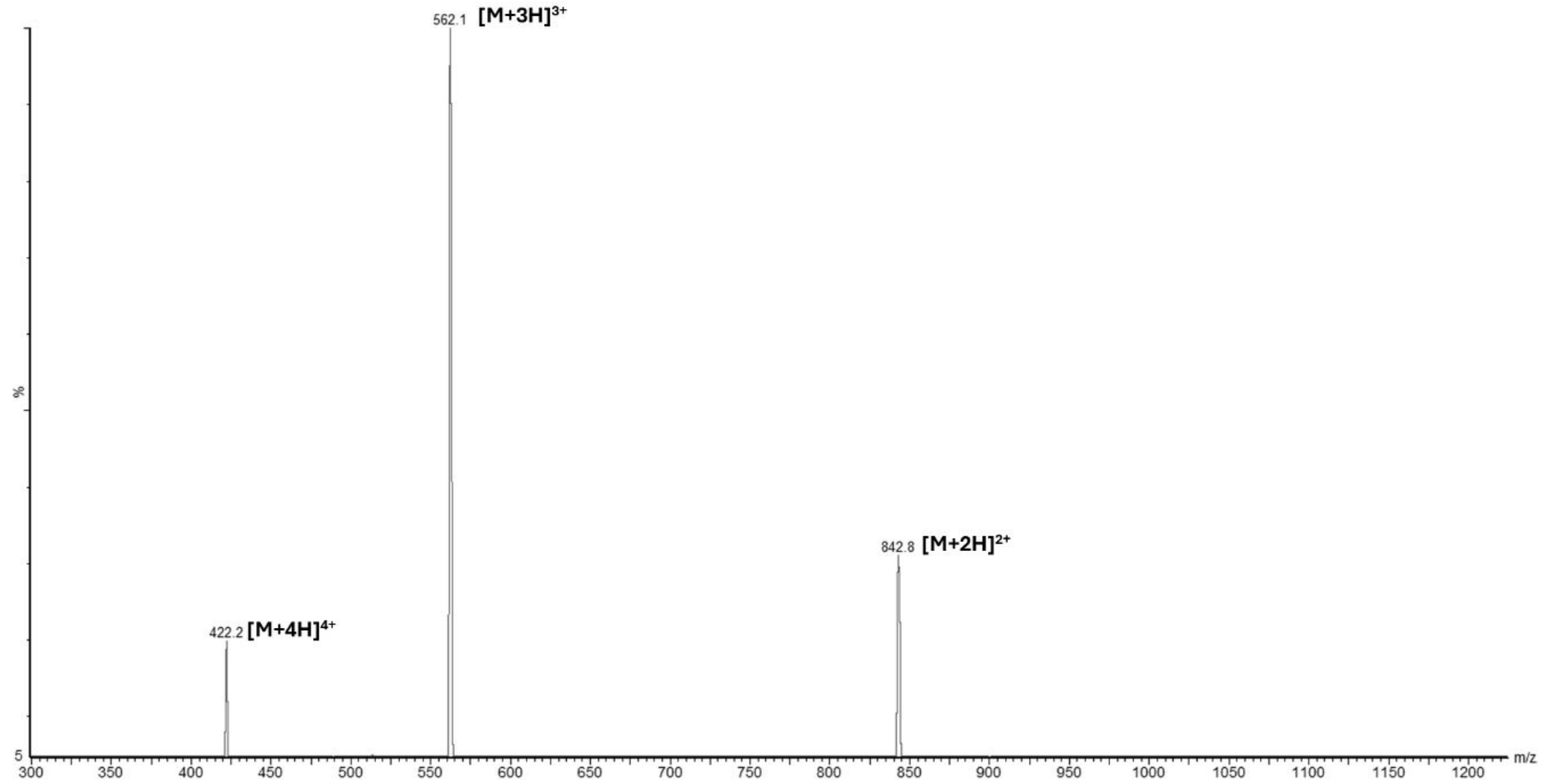
562.1 [M+3H]3+
842.8 [M+2H]2+
422.2 [M+4H]4+
%
m/z

*Compound **3c***

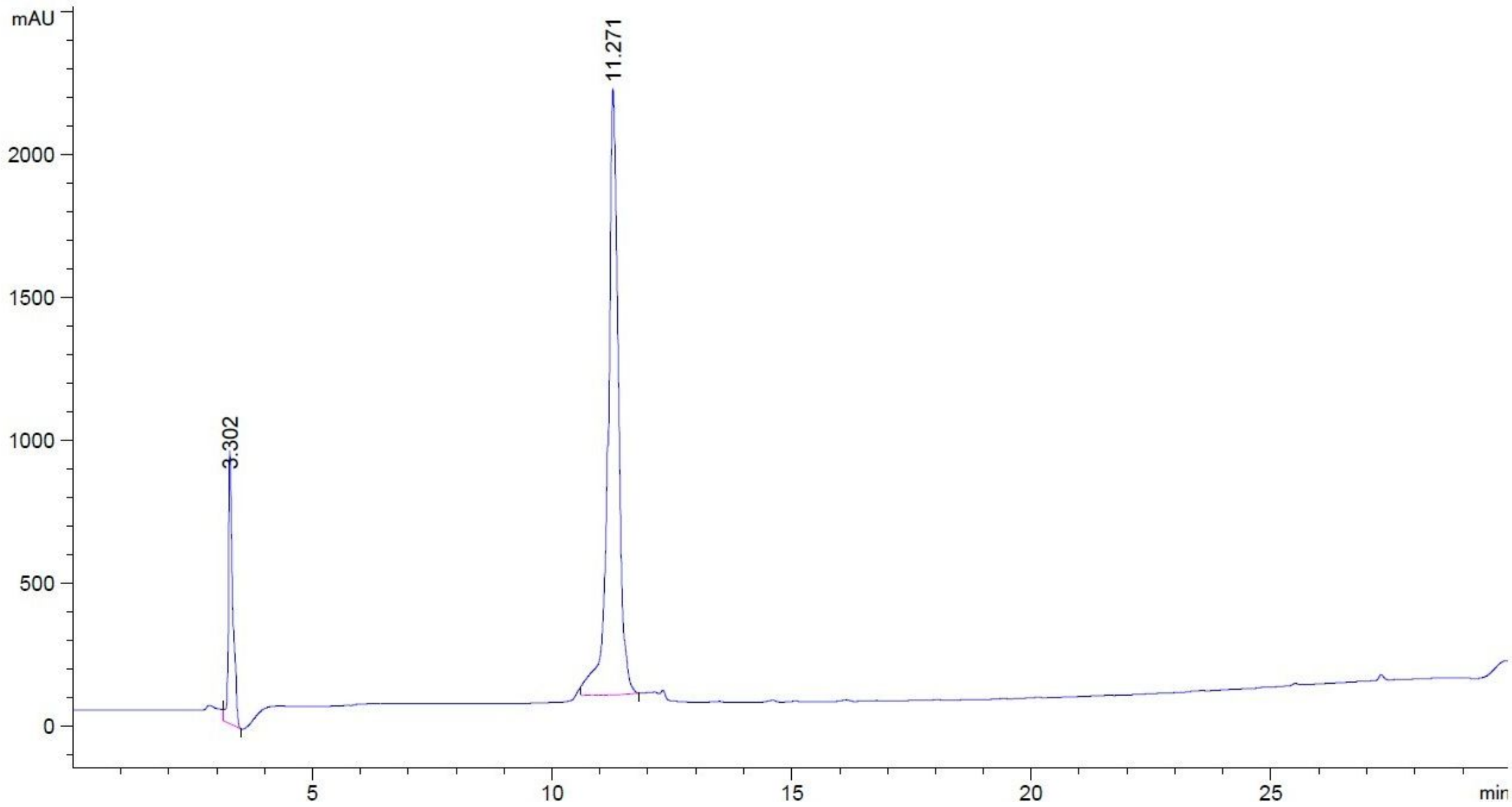
mAU
11.271
3.302
2000
1500
1000
500
0
5
10
15
20
25
min

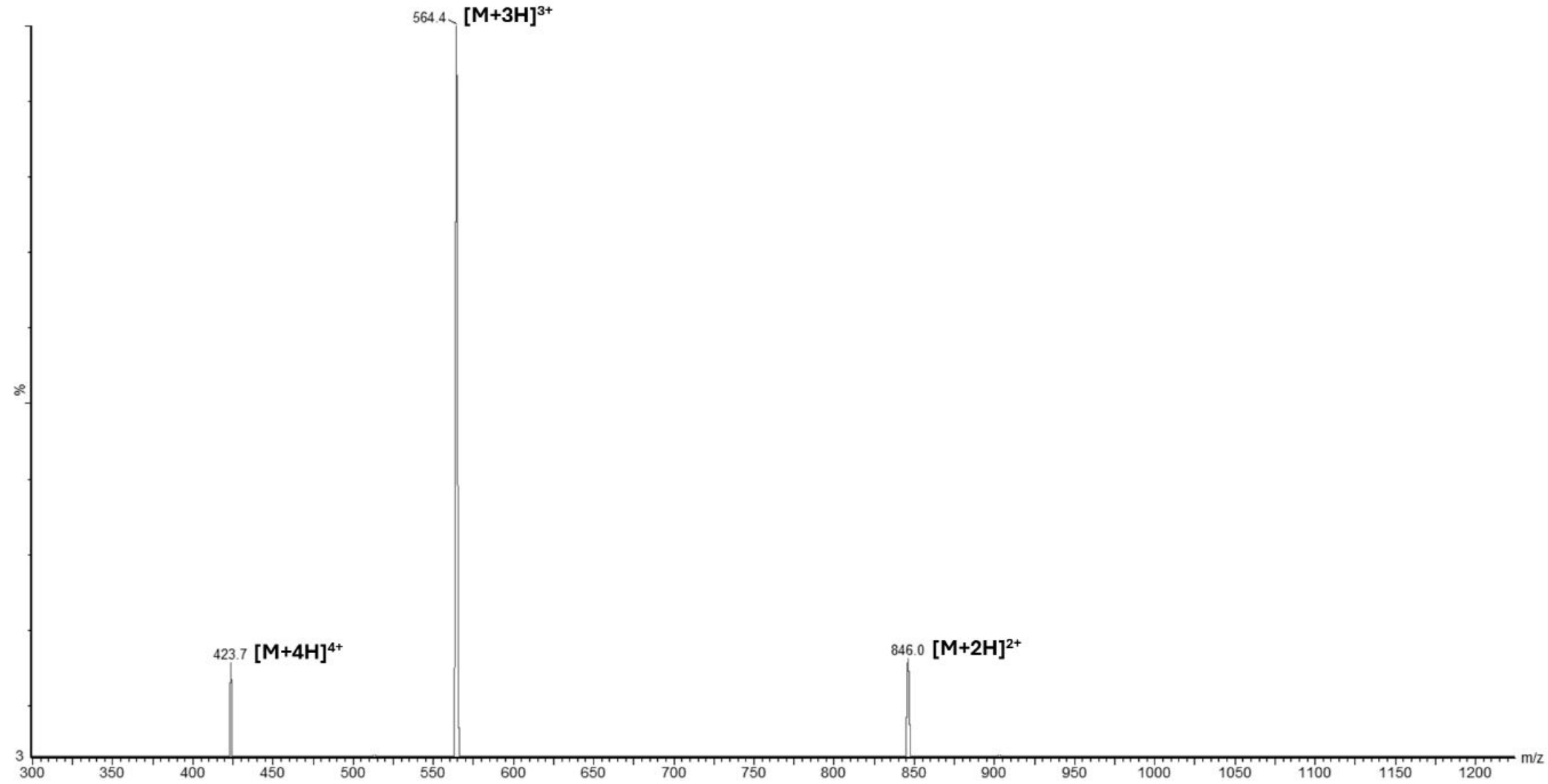
564.4 [M+3H]3+
423.7 [M+4H]4+
846.0 [M+2H]2+
%
3
300
350
400
450
500
550
600
650
700
750
800
850
900
950
1000
1050
1100
1150
1200
m/z

*Compound **3d***

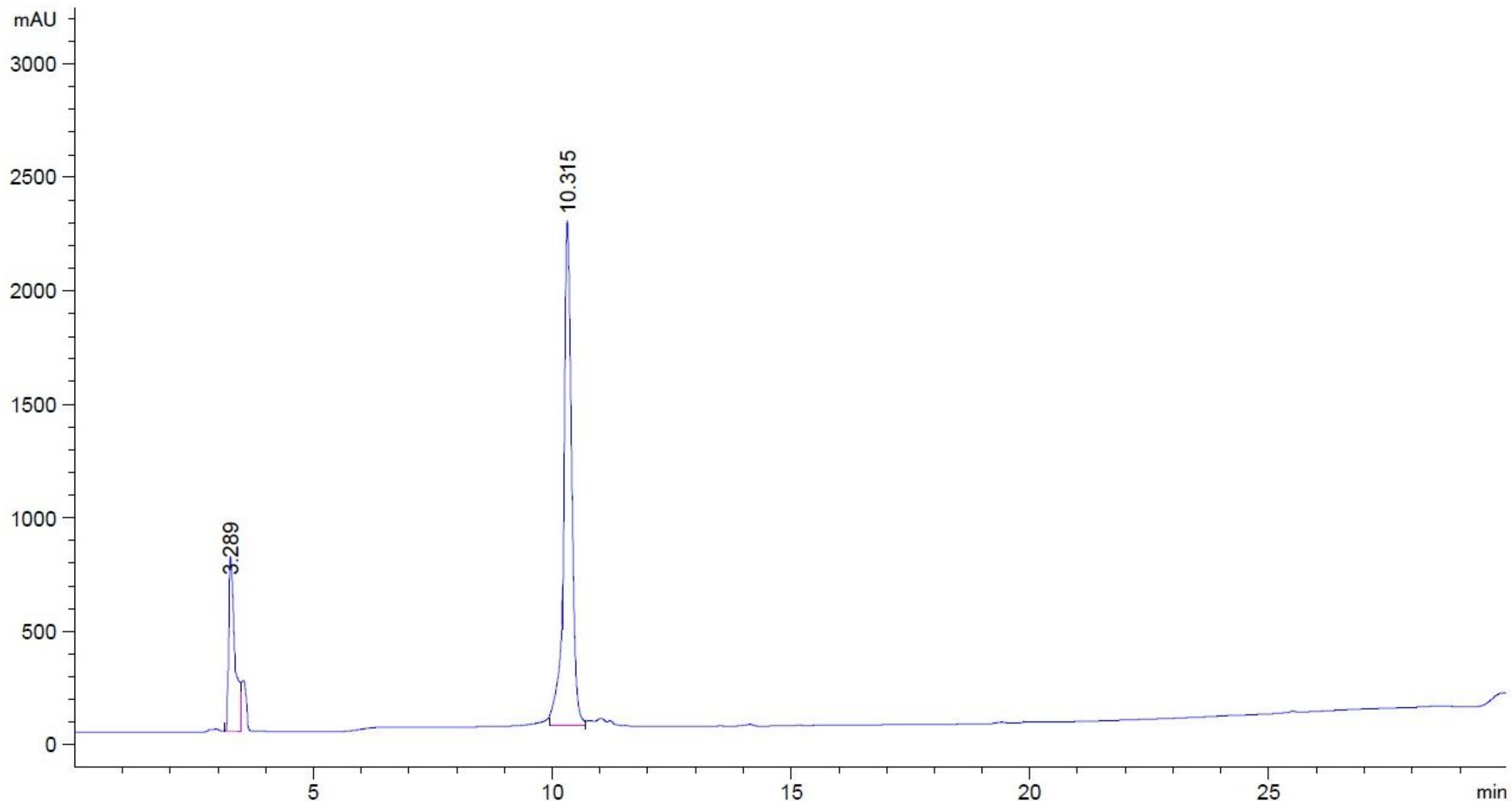

mAU
3000
2500
2000
1500
1000
500
0
10.315
3.289
5
10
15
20
25
min

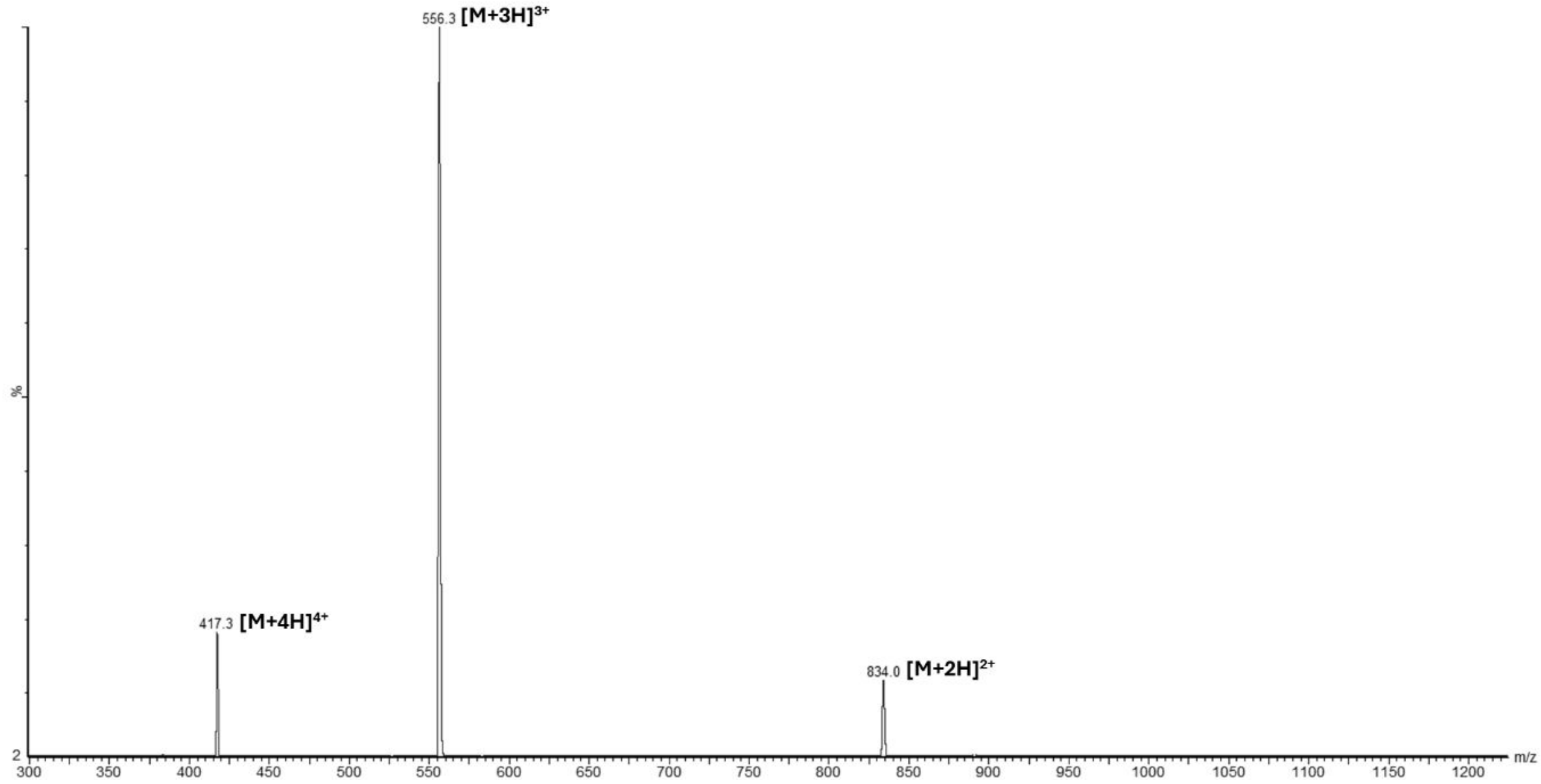

556.3 [M+3H]3+
417.3 [M+4H]4+
834.0 [M+2H]2+
%
2
300
350
400
450
500
550
600
650
700
750
800
850
900
950
1000
1050
1100
1150
1200
m/z
